# The 2026 guided acoustic waves roadmap

**DOI:** 10.1088/1361-6463/ae258d

**Published:** 2026-03-02

**Authors:** Hubert J Krenner, Paulo V Santos, Christoph Westerhausen, Gustav Andersson, Andrew N Cleland, Hermann Sellier, Shintaro Takada, Christopher Bäuerle, Daniel Wigger, Tilmann Kuhn, Paweł Machnikowski, Matthias Weiß, Galan Moody, Alberto Hernández-Mínguez, Snežana Lazić, Alexander S Kuznetsov, Matthias Küß, Manfred Albrecht, Mathias Weiler, Jorge Puebla, Yunyoung Hwang, Yoshichika Otani, Krishna C Balram, I-Tung Chen, Keji Lai, Mo Li, Geoff R Nash, Emeline D S Nysten, Paromita Bhattacharjee, Himakshi Mishra, Parameswar K Iyer, Harshal B Nemade, Abdelkrim Khelif, Sarah Benchabane, Gao Feng, Yabin Jin, Ausrine Bartasyte, Samuel Margueron, Massimiliano Marangolo, Laura Thevenard, Pauline Rovillain, Catherine Gourdon, Sami Hage-Ali, Omar Elmazria, Hagen Schmidt, Leslie Y Yeo, Lizebona A Ambattu, Jessie S Jeon, Daesik Kwak, Joseph Rufo, Shujie Yang, Tony Jun Huang

**Affiliations:** 1 Physikalisches Institut, Universität Münster, Wilhelm-Klemm-Straße 10, Münster 48149, Germany; 2 Paul-Drude-Institut für Festkörperelektronik, Leibniz-Institut im Forschungsverbund Berlin e.V., Hausvogteiplatz 5-7, Berlin 10117, Germany; 3 Institute of Theoretical Medicine, Physiology, University of Augsburg, Augsburg 86159, Germany; 4 Pritzker School of Molecular Engineering, University of Chicago, Chicago, IL 60637, United States of America; 5 Argonne National Laboratory, Lemont, IL 60439, United States of America; 6 Université Grenoble Alpes, CNRS, Grenoble INP, Institut Néel, Grenoble 38000, France; 7 Department of Physics, Graduate School of Science, Osaka University, Toyonaka 560-0043, Japan; 8 Institute for Open and Transdisciplinary Research Initiatives, Osaka University, Suits 560-8531, Japan; 9 Center for Quantum Information and Quantum Biology (QIQB), Osaka University, Osaka 565-0871, Japan; 10 Department of Physics, Universität Münster, Wilhelm-Klemm-Str. 9, 48149 Münster, Germany; 11 Institute of Solid State Theory, Universität Münster, Wilhelm-Klemm-Str. 10, Münster 48149, Germany; 12 Institute of Theoretical Physics, Wrocław University of Science and Technology, Wybrzeże Stanisława Wyspiańskiego 27, 50-370 Wrocław, Poland; 13 Electrical and Computer Engineering Department, University of California, Santa Barbara, CA 93106, United States of America; 14 Departamento de Física de Materiales, Instituto Universitario de Ciencia de Materiales ‘Nicolás Cabrera’ (INC) and Condensed Matter Physics Center (IFIMAC), Universidad Autónoma de Madrid (UAM), 28049 Madrid, Spain; 15 Institute of Physics, University of Augsburg, 86135 Augsburg, Germany; 16 Fachbereich Physik and Landesforschungszentrum OPTIMAS, Rheinland-Pfälzische Technische Universität Kaiserslautern-Landau, 67663 Kaiserslautern, Germany; 17 Center for Emergent Matter Science, RIKEN, Wako 351-0198, Japan; 18 Department of Electronic Science and Engineering, Kyoto University, Kyoto 615-8510, Japan; 19 Institute for Solid State Physics, University of Tokyo, Kashiwa 277-8581, Japan; 20 Quantum Engineering Technology Labs and Department of Electrical and Electronic Engineering, University of Bristol, Bristol BS8 1UB, United Kingdom; 21 Department of Electrical and Computer Engineering, University of Washington, Seattle, WA 98195, United States of America; 22 Department of Physics, The University of Texas at Austin, Austin, TX 78712, United States of America; 23 Department of Physics, University of Washington, Seattle, WA 98195, United States of America; 24 Natural Sciences, The University of Exeter, Exeter, United Kingdom; 25 Department of Electronics and Communication Engineering, Manipal Institute of Technology, MAHE, 560064 Bangalore, India; 26 Department of Chemistry, Indian Institute of Technology Guwahati, 781039 Amingaon, India; 27 Department of Electronics and Electrical Engineering, Indian Institute of Technology Guwahati, 781039 Amingaon, India; 28 Institut FEMTO-ST, UMR6174 CNRS, Université de Franche-Comté, Besançon, France; 29 College of Science and Engineering, Hamad Bin Khalifa University, Doha, Qatar; 30 ZJU-HangZhou Global Scientific and Technological Innovation Center, Zhejiang University, Hangzhou, People’s Republic of China; 31 Institute of Computational Mechanics × AI & College of Intelligent Robotics and Advanced Manufacturing, Fudan University, Shanghai, 200433, People’s Republic of China; 32 Université Marie et Louis Pasteur, ENSMM, FEMTO-ST Institute, 26 rue de l’Epitaphe, 25030 Besançon, France; 33 Université Paris-Saclay, CNRS, Centre de Nanosciences et de Nanotechnologies, 10 Bd Thomas Gobert, 91120 Palaiseau, France; 34 Institut Universitaire de France, 103 boulevard Saint Michel, 75005 Paris, France; 35 Sorbonne Université, CNRS, Institut des NanoSciences de Paris, INSP, 4 place Jussieu, F-75005 Paris, France; 36 Université de Lorraine, CNRS, IJL, F-54000 Nancy, France; 37 Leibniz Institute for Solid State and Materials Research, Helmholtzstr. 20, 01069 Dresden, Germany; 38 Micro/Nanophysics Research Laboratory, RMIT University, Melbourne, VIC 3001, Australia; 39 KAIST, Daejeon, Republic of Korea; 40 Department of Mechanical Engineering and Materials Science, Duke University, Durham, NC 27708, United States of America

**Keywords:** guided acoustic waves, surface acoustic waves, phononics

## Abstract

Guided elastic waves are a truly cross-disciplinary key enabling technology. For more than five decades, surface acoustic wave (SAW) and bulk acoustic wave devices find widespread applications. Nowadays, different types of guided elastic waves cover the wide spectrum of applications spanning from quantum technologies to the life sciences, from controlling single excitations to macroscopic collective states in condensed matter. Six years after the first 2019 SAW roadmap, we believe it is time to make a step back and take a fresh look at the status of the field and its future challenges. Since the first roadmap in 2019, the spectrum clearly expanded and this new edition presents a current snapshot of the status of this vibrant field and prospects for potential future developments.

## 1. Introduction

### Hubert J Krenner^1^, Paulo V Santos^2^ and Christoph Westerhausen^3^

^1^ Physikalisches Institut, Universität Münster, Wilhelm-Klemm-Straße 10, 48149 Münster, Germany

^2^ Paul-Drude-Institut für Festkörperelektronik, Leibniz-Institut im Forschungsverbund Berlin e.V., Hausvogteiplatz 5–7, 10117 Berlin, Germany

^3^ Institut für Theoretische Medizin, Physiologie, Universität Augsburg, Universitätstraße 2, 86159 Augsburg, Germany

E-mail: krenner@uni-muenster.de, santos@pdi-berlin.de and christoph.westerhausen@uni-a.de

Guided elastic waves are a truly cross-disciplinary key enabling technology. For more than five decades, surface acoustic wave (SAW) and bulk acoustic wave (BAW) devices find widespread applications. Unarguably one of the most prominent examples is information technology: here mass-produced and inexpensive micro- and nanoacoustic devices form the backbone for wireless signal processing at radio and microwave (MW) frequencies [1, 2]. These devices deliberately exploit the—compared to electromagnetic waves—very low phase velocity of elastic waves, shrinking the wavelength of gigahertz frequencies from the centimetre to the (sub-)micrometre scale. Piezoelectric substrates or thin films enable efficient transduction between the mechanical and electrical domains.

Due to their pervasive nature, mechanical waves couple to literally any system embedded in or connected to the propagation medium, thus making them universal transducers. In piezoelectrics, the mechanical coupling is complemented by large electric fields driving or detecting charge dynamics. Prominent examples are the acoustic control and spectroscopy of optoelectronic properties or collective excitations in solid-state systems [3,4,5]. In life sciences, SAW technology is used in an entire class of lab-on-chip devices. These thumbnail-sized labs manipulate or sense tiniest amounts of liquids, colloids, cells or molecules [6, 7]. Nowadays, different types of guided elastic waves cover the wide spectrum of applications spanning from quantum technologies to the life sciences, from controlling single excitations to macroscopic collective states in condensed matter.

In 2015, the innovative training network (ITN) ‘SAWtrain’ funded by the European Union’s Marie Sklodowska Curie Actions established a network of several leading groups covering the diverse spectrum of SAW research and technologies. In SAWtrain, we initiated the very successful ‘Special Issue on Surface Acoustic Waves in Semiconductor Nanosystems’ in *Journal of Physics D: Applied Physics* [8] comprising topical reviews and original research articles from groups working across the globe. When running this special issue, we realized that many groups were challenged by the breadth of the field. Thus, there was a strong need in the community for a forward-looking overview article on the status of the field and the challenges laying ahead. This sparked the idea of putting together a roadmap with contributions by leading groups. The roadmap manuscript entitled ‘2019 Surface Acoustic Waves Roadmap’ [9] appeared in the *Journal of Physics D: Applied Physics* in July 2019, concluding the above-mentioned special issue. This roadmap presented a snapshot of the status of this vibrant field at that time and drew a picture of potential future developments. Since then, many of these that-time perspectives have been flourishing and new routes have been opening.

Now, six years after the first roadmap, we believe it is time to make a step back and take a fresh look at the status of the field and its future challenges. Since the first roadmap in 2019, the spectrum clearly expanded. New directions appeared while other topics shifted out of focus. The most prominent change is the title itself: the ‘Surface Acoustic Waves Roadmap’ of 2019 changes to ‘Guided Elastic Waves Roadmap’. The new title reflects that SAWs are only one—yet a very prominent—type of guided elastic waves presently investigated and harnessed in the community. Moreover, the number of opinion pieces increased from 15 to 18 underpinning the growth and increasing diversity of the field.

**Figure 1.**
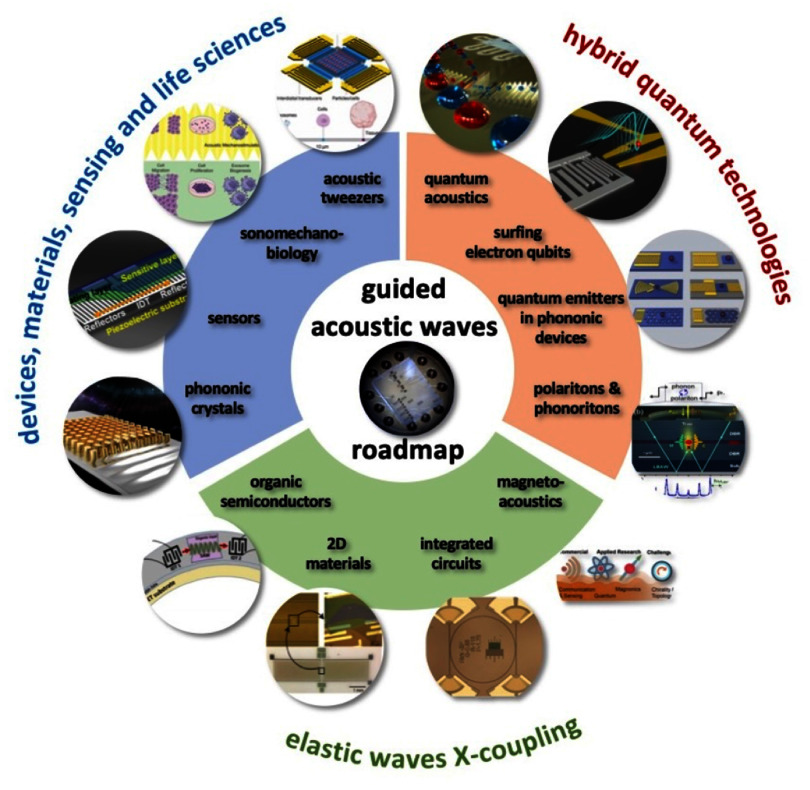
Areas of guided acoustic wave research covered in this roadmap.

The topics covered in these opinion pieces span again the full cross-disciplinary spectrum from quantum acoustics to the life sciences. Figure 1 gives a graphical overview of these topics covering three main areas each comprising six opinion pieces.

i. In the areas of ‘hybrid quantum technologies’ the universal nature of phonon interactions is deliberately exploited for quantum transduction between different degrees of freedom to single quantum systems and macroscopic quantum phases. Contributions span from quantum acoustics where single phonons are carriers of quantum information, interfacing single quantum emitters with phonons to phonotrions where strong coupling between light (photons), sound (phonons), and matter (excitons) is pioneered.
ii. In ‘elastic waves X-coupling’, the plethora of fundamental phonon couplings to collective excitations in emerging solid-state quantum nanosystems in novel materials are prospected and applied for dynamic control in integrated phononic and optomechanical (OM) circuitry. Contributions cover from magneto-acoustics, two-dimensional (2D) materials, organic semiconductors (OSs) and integrated circuit harnessing phonons.
iii. The area ‘devices, materials, sensing and life sciences’ bridges from phononic crystals, novel piezoelectric materials to novel sensors and for actuation. Contributions natively cover a diverse spectrum for instance new ultra-strong piezoelectric materials, new paradigms of acoustic wave sensors, and actuation and manipulation in mechanobiology and acoustofluidic tweezers.

This roadmap concludes the special issue on guided elastic waves for hybrid nano- and quantum technologies [10]. This special issue is a collection of timely topical reviews and original research articles. Three topical reviews cover recent advances in SAW modulated 2D materials and van der Waals systems [11], acousto-opto-electronic hybrids comprising OSs and SAWs [12], and electron qubits surfing on acoustic waves [13]. Original research articles report on important work covering the entire spectrum from quantum technologies [14, 15], devices concepts based on novel material systems [16, 17], acoustofluidics and streaming [8, 18, 19] to acoustically stimulated wound healing [20].

During preparation of the final manuscript for submission, we learned that Sarah Benchabane, an initiator of this roadmap, lost her battle against cancer. Deeply saddened by this untimely passing, we dedicate this roadmap to Sarah’s memory.

## 2. Surface acoustic waves in the quantum limit

### G Andersson^1^ and A N Cleland^1,2^

^1^ Pritzker School of Molecular Engineering, University of Chicago, Chicago, IL 60637, United States of America

^2^ Argonne National Laboratory, Lemont, IL 60439, United States of America

E-mail: gandersson@uchicago.edu and anc@uchicago.edu

### Status

The rapid and recent development of quantum acoustics, studying the interactions between mechanical fields and matter at the quantum level, has mostly been advanced by integrating superconducting qubits with mechanical devices [24]. Superconducting quantum circuits typically exhibit large zero-point electric field fluctuations and can be engineered to interface with the transducer structures and piezoelectric materials used for SAWs and other acoustic devices [25]. Early implementations were limited by the reduced performance of superconducting circuits fabricated on the piezoelectric substrates that support SAW transduction. Nonetheless, these devices enabled reproducing a variety of phenomena associated with quantum optics and cavity quantum electrodynamics with phonons instead of photons. Examples include strong coupling of a qubit to propagating SAWs [26] as well as vacuum Rabi splitting in a SAW resonator [27, 28].

**Figure 2.**
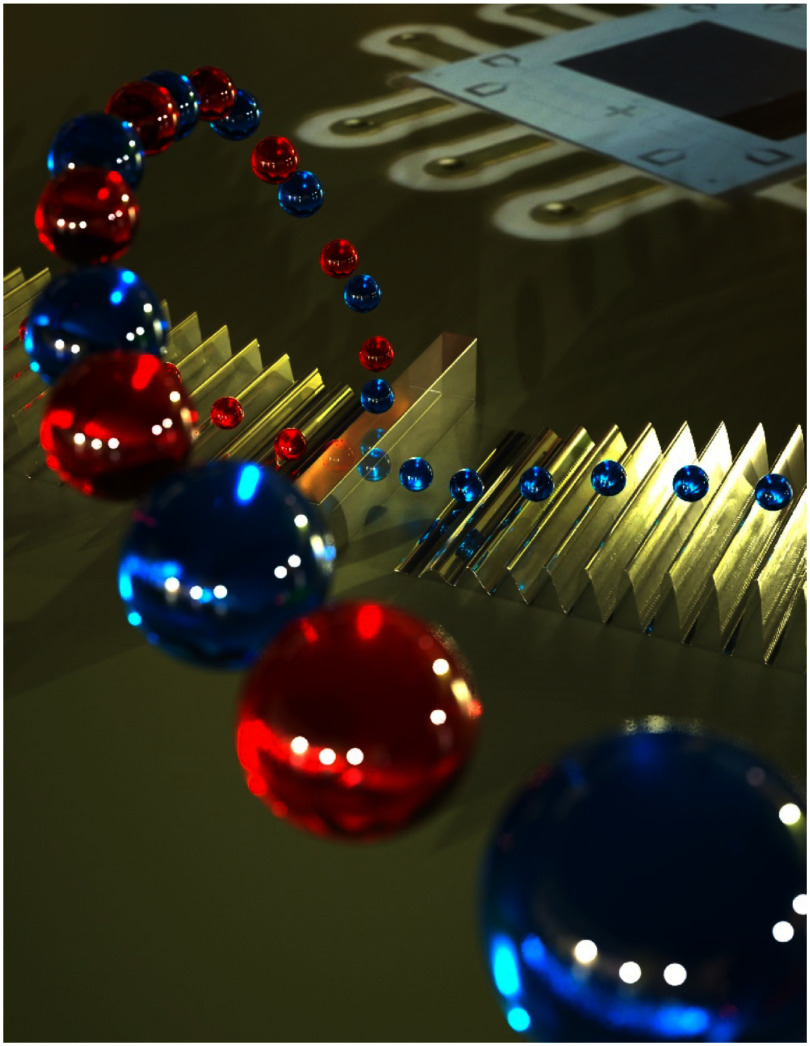
Artistic rendering of a phononic beamsplitter with incident single-phonon wavepackets. Credit: Peter Allen, Second Bay Studios.

More recently, flip-chip assemblies combining standard dielectrics supporting the qubit circuit, assembled in a flip-chip geometry with bulk and SAW devices including piezoelectric materials, have largely mitigated the qubit performance issue and allowed a wide range of qubit operations as well as dynamically tunable SAW-qubit interactions [29]. Now, qubit gates and quantum state readout can be applied with the coupling to the acoustics turned off, ensuring high fidelity operations. These capabilities have been leveraged for more advanced experiments, such as using SAWs to generate entanglement between qubits [30]. In addition an acoustic beamsplitter has been used to both demonstrate the generation of entangled single-phonon states, whose entanglement can be transferred to a pair of qubits, as well as for exploring the Hong–Ou–Mandel effect with phonons [31]. The latter includes measuring the interference of two single phonons at the beamsplitter, where their simultaneous arrival generates an entangled two-phonon output state. An artistic illustration of this phenomenon is shown in figure 2.

**Figure 3.**
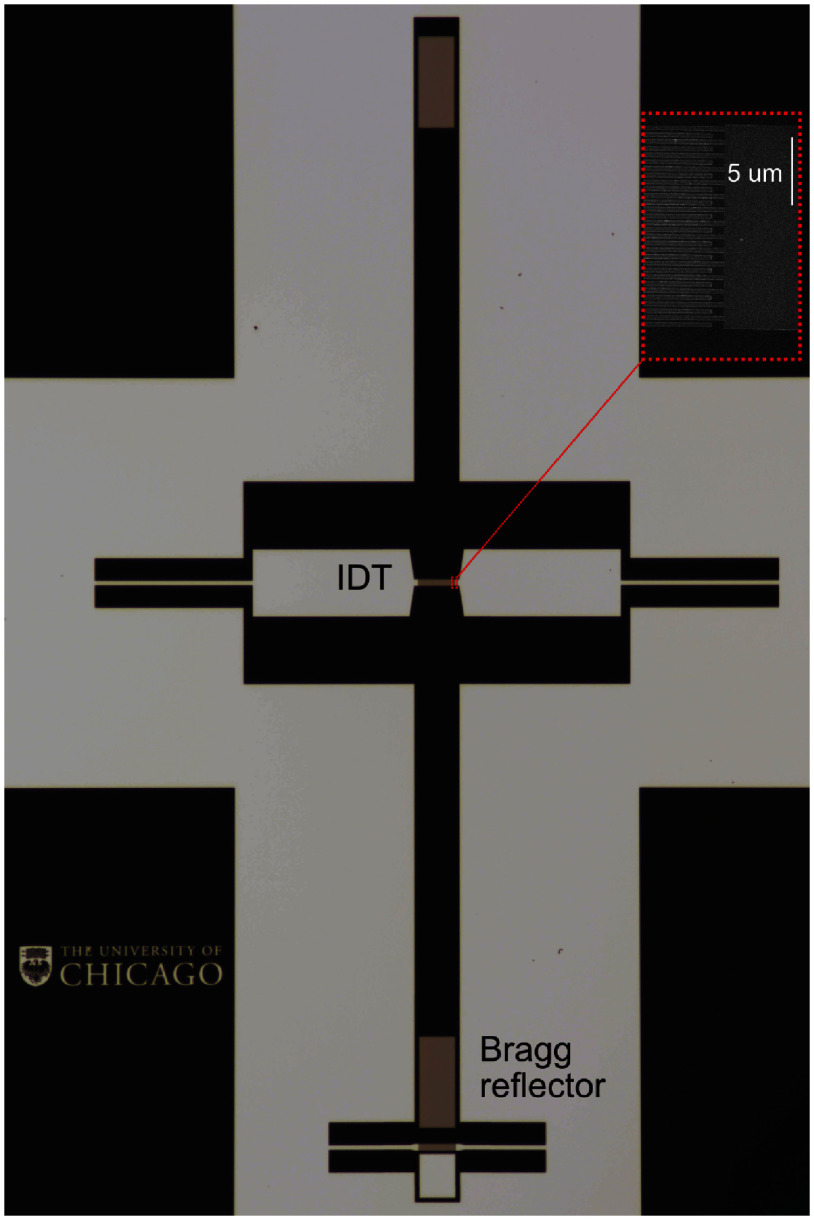
SAW resonator on lithium niobate with an interdigitated transducer at the centre and mirrors on either side. Similar to a device shown in [30]. Inset shows SEM image of IDT fingers.

The progress that has been made in controlling phonons with superconducting qubits is encouraging the sizable step from demonstrations of quantum physics to applications in quantum information processing. The short wavelength and slow (acoustic) propagation velocity of SAWs may provide opportunities for time and frequency multiplexing that are more challenging to realize with purely superconducting MW hardware, where one must instead control interactions at the 10^5^ times higher speed of light, changing the interaction time scale from tens of nanoseconds to fractions of a picosecond. Figure 3 shows an acoustic channel where the round-trip propagation time of a SAW pulse is an order of magnitude longer than the timescale of its emission from a coupled qubit. SAWs also interface in a natural way with other solid-state quantum systems, such as semiconductor and insulator colour centres [32], as well as OM devices, facilitating integration into quantum networks.

### Current and future challenges

#### Coherent control

A remarkable degree of quantum control of SAW phonons has recently been demonstrated, providing the building blocks for quantum optics, computing, and sensing with propagating phonons. Realising the full potential of this technology will require integrating single phonon sources, beamsplitters, phase shifters, as well as detectors on the same device. The quantum toolbox for propagating phonons is promising for implementing circuits originally envisioned for photonic quantum computing, with the additional resource of strong coupling to superconducting qubits.

#### Entangling SAW resonators

SAW resonators have a compact footprint, couple efficiently to superconducting qubits and can be engineered to support single or multimode operation. This makes them an intriguing candidate for quantum information processing applications. Multimode devices are suitable for implementing quantum RAM [33], as well as quantum computing with bosonic encodings of qubits [34]. Deterministically preparing entangled states between modes in separate SAW resonators would be an important step towards more complex protocols [35].

#### Coupling to solid-state defects

Recent progress with highly strain-susceptible colour centres [32] in diamond has opened new avenues for interfacing superconducting qubits with other solid-state quantum systems via SAWs. Qubit state transitions in these defects in some cases are likely more addressable using strain than MW fields [36], making SAW-coupled superconducting qubits a promising approach to quantum control. Furthermore, optical transitions of colour centres could be exploited for quantum MW-to-optical transduction [37].

### Advances in science and technology to meet challenges

#### Improving coherence times.

While highly coherent mechanical devices have been demonstrated in the quantum regime [38], long lifetimes have proven challenging to combine with strong qubit-phonon coupling; SAW propagation loss also remains a critical limiting factor for future development of SAW-qubit systems. Much like the progress made in recent years in improving the coherence of superconducting qubits and electromagnetic resonators, reducing the loss in SAW devices will require both improved materials and fabrication processes [39]. Phononic bandgap designs could play an important role in the development of highly coherent quantum acoustics. A potential avenue is the use of non-piezoelectric materials known to support high-quality mechanical modes such as Si and SiN [38, 40], where piezoelectric thin films could be integrated locally to enable transduction.

#### Directional emission.

Quantum control of phonons on a chip would be greatly facilitated by transducers with directional control [34]. Unidirectional transducers have been used with qubits by combining a standard interdigitated transducer with a Bragg reflector [41]. More complex phononic circuits would benefit from a higher degree of control, enabling directional emission and transmission of SAW phonons.

#### Frequency response engineering.

SAW transducers are distributed structures that span many wavelengths, which gives rise to a frequency-dependent coupling strength. Tailoring the frequency response to the level structure of the superconducting qubits could offer new functionality, much as it has done for the processing of signals in classical SAW devices (chirping, frequency selectivity, etc).

### Concluding remarks

Coupling superconducting qubits to SAWs has enabled demonstrations of an impressive range of quantum effects with phonons. It is an open question what role SAWs will play in future quantum information technologies, but developments to date suggest it is not unlikely that quantum acoustics could have a significant impact in quantum information as well as sensing and communication.

## Acknowledgements

We acknowledge support from the Air Force Office of Scientific Research (AFOSR Grant FA9550-20-1-0364); the Army Research Office (ARO Grant W911NF2310077). Results are in part based on work supported by the U.S. Department of Energy Office of Science National Quantum Information Science Research Center Q-NEXT. This work was partially supported by UChicago’s MRSEC (NSF Award DMR-2011854) and by the NSF QLCI for HQAN (NSF Award 2016136), as well as the Pritzker Nanofabrication Facility, which receives support from SHyNE, a node of the National Science Foundation’s National Nanotechnology Coordinated Infrastructure (NSF Grant No. NNCI ECCS-2025633).

## 3. Surfing electrons for quantum technology

### Hermann Sellier^1^, Shintaro Takada^2,3,4^ and Christopher Bäuerle^1^

^1^ Université Grenoble Alpes, CNRS, Grenoble INP, Institut Néel, 38000 Grenoble, France

^2^ Department of Physics, Graduate School of Science, Osaka University, Toyonaka 560-0043, Japan

^3^ Institute for Open and Transdisciplinary Research Initiatives, Osaka University, Suits, 560-8531, Japan

^4^ Center for Quantum Information and Quantum Biology, Osaka University, Osaka 565-0871, Japan

E-mail: hermann.sellier@neel.cnrs.fr, takada@phys.sci.osaka-u.ac.jp and christopher.bauerle@neel.cnrs.fr

### Status

Over the past decade, significant progress has been made in the field of single electron transport, in particular utilising SAWs with the goal of realising flying qubits with electrons [42]. A notable challenge has been improving the efficiency of transferring electrons between distant quantum dots (QDs) in GaAs/AlGaAs heterostructures. Initial efforts, as described in pioneering studies [43, 44], achieved transfer efficiencies of around 90%. However, recent developments have pushed this figure well above 99% [45], over distances exceeding 60 microns [46].

**Figure 4.**
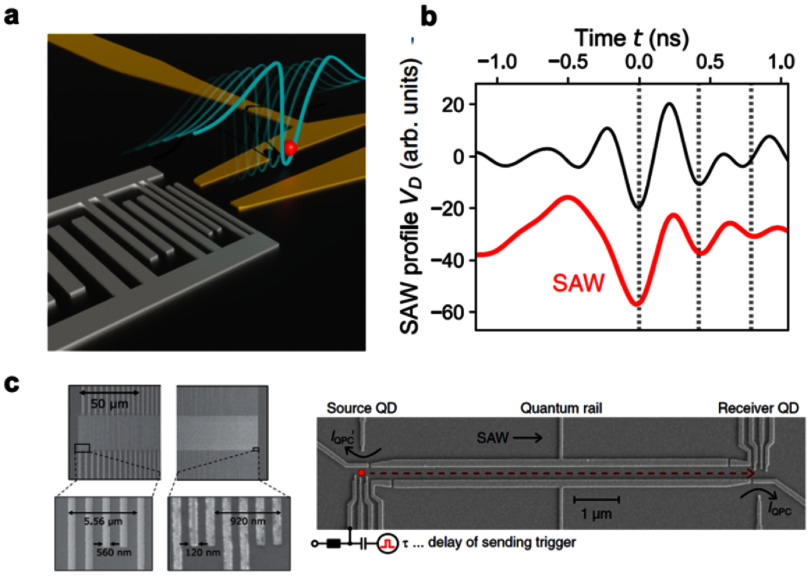
A sound pulse transports a single electron. (a). Artistic image of single electron transfer driven by a single-cycle SAW pulse emitted by a chirp transducer. (b). Shape of the SAW pulse generated by the chirp IDT shown in (c) and measured with a second high-bandwidth detector IDT (black line). The actual SAW pulse shape is calculated taking into account the finite bandwidth of the detector (red line). (c). Scanning electron micrograph of the chirp IDT (left) and the device for single electron transfer (right). The bandwidth of the chirp transducer extends from 0.5 GHz to 3 GHz and a single electron transfer efficiency of 99.4% has been obtained with this single-cycle pulse technique. Reproduced from [47]. CC BY 4.0.

Synchronising two individual electrons represented a second key challenge in the perspective of realising a quantum gate. SAW trains typically consist of several tens of periods, making the loading of an electron, from a static QD into one of the moving potential wells, a probabilistic process. Nevertheless, using ultrafast gate pulsing, researchers can now deterministically load a single electron into a desired SAW minimum [45]. Recently, a more refined approach has been introduced, using chirp transducers with broad bandwidths, to engineer SAWs with only a single minimum—a single-cycle acoustic pulse—as highlighted in figure 4 [47]. Its advantage lies in the straightforward synchronisation of multiple SAW-based single electron sources with minimal hardware additions (no need for ultrafast gate pulsing). The synchronisation capability between two individual electrons has enabled collision experiments at the single electron level. These single-shot experiments revealed strong antibunching of electron pairs impinging on a beam splitter, with a key role played by Coulomb interactions [46]. Such a setup was also used to demonstrate the appearance of a Coulomb liquid phase in droplets with as few as three electrons [48].

Another significant challenge has been demonstrating coherent single spin transport and entanglement using SAWs. This can be realised by preparing a two-electron singlet state in a double QD, then separating the electrons with precise timing and assessing coherence afterwards [49]. The concept revolves around sequentially loading two entangled electron spins into separate SAW minima and varying their separation distance. Measurement of the singlet state probability upon their arrival at the receiver double QD, along with observation of coherent oscillations of this probability as a function of the separation time and applied external magnetic field, demonstrates distant spin entanglement. This achievement marks a pivotal step towards achieving rapid, on-chip deterministic interconnection of distant qubits within semiconductor-based quantum circuits.

By leveraging all these technological advancements from the past decade, it becomes feasible to implement real-time manipulation of the quantum state of a SAW-transported electron, paving the way for the development of the first flying electron qubit. Note that the SAW is here a classical object (only the electron holds the quantum information), but there are other quantum technological studies in which the SAW is the qubit (therefore also a flying qubit). This approach is detailed in section 02 on quantum acoustics where quantized SAWs are interfaced with superconducting qubits.

### Current and future challenges

One objective in the field of SAW-assisted single-electron transport is to realise flying charge qubits where the quantum information is coded in the quantum delocalisation over two transport paths. In this type of qubit, the SAW is employed to transport the electron at constant velocity along two quasi-parallel channels, locally coupled to split the probability over the two qubit states, and locally separated to control their phase difference using electric or magnetic fields. To achieve such a flying qubit, the transported electron should occupy a well-defined quantum state and remain coherent all along the fly. This condition is probably the biggest challenge of this field since the SAW carries the electron across a depleted region without any screening by conducting electrons, which makes such an isolated charge qubit very sensitive to electrostatic background noise and disorder. Local potential variations of a few tens of meV along the transport path are responsible for transitions of the transported electron from its ground state to excited states, even if the typical speed of the SAW is much smaller than Fermi velocities in conducting systems. Such excitations may arise during the injection of the single electron by the SAW into the channel, or later, at channel discontinuities. Solutions to this issue involve increasing the level spacing of the moving QD and designing devices with flatter potential profiles. In a recent experiment on a tunable beam-splitter, a signature of charge coherence has been observed, however with very limited visibility, using a continuous flow of single electrons [50].

**Figure 5.**
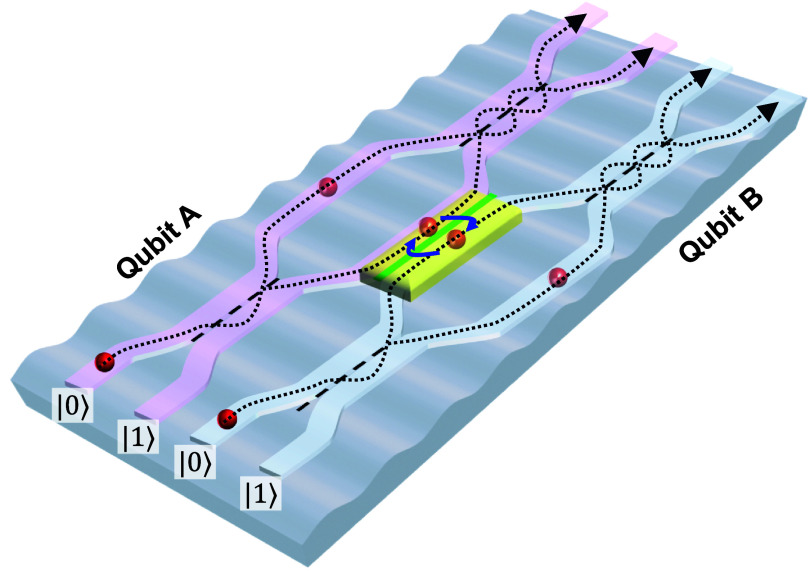
Two-qubit gate for flying charges. Two flying qubits are coupled via Coulomb interaction to implement a controlled phase gate for SAW flying electrons. The dashed lines correspond to beam splitters and the green line to an energy barrier that forbids tunnelling from one qubit to the other, but allows for Coulomb interaction. Two electrons are injected simultaneously into qubit A (target qubit) and qubit B (control qubit) transported by a SAW. In the coupling region (highlighted in yellow), a phase shift is induced by the control qubit to the target qubit, flipping the state of the target qubit from |0> to |1> (and vice versa).

The last main step in the development of SAW-based flying qubits will be the creation of a two-qubit gate by implementing a controlled phase gate, which performs a conditional phase rotation on one qubit depending on the quantum state of the second qubit. This operation can be achieved thanks to the Coulomb interaction between the two electrons of the two qubits as depicted in figure 5. Previous collision experiments have demonstrated the relevance of this coupling protocol by estimating the strength of the Coulomb repulsion between two electrons flying in coupled channels [46]. Since this interaction is rather strong, a precise control of the interaction time in the picosecond regime will be necessary. With such a two-qubit gate, it will be possible to perform the first test of Bell’s inequalities with electrons in a semiconductor device. The experiment will consist in preparing an entangled state with the Coulomb coupler and measuring the correlations at the output for specific directions of the qubits’ isospins [51].

### Advances in science and technology to meet challenges

In order to obtain a phase coherent flying qubit in a controlled quantum state, the moving QD should be more robust against potential variations during the flight, which requires a larger spacing between the quantum levels. Recent experiments [52] have shown that a SAW amplitude above 20 mV (24 dB applied SAW power) maintains the electron inside the moving SAW QD without tunnelling to a neighbouring SAW minimum. However, to preserve quantum coherence, the longitudinal confinement should be increased further to reach a level spacing similar to the level spacing of the transverse confinement inside the transport channel where 1D subbands are separated by typically 4 meV for 300 nm-spaced surface gates.

One possible direction to increase the SAW amplitude is to improve the coupling efficiency of the interdigital transducer (IDT) by overcoming impedance mismatch of the transducers, testing new electrode geometries and materials. Another direction is to focus the energy of the mechanical wave in a single direction and in a reduced sample volume using focussing IDTs [53]. A third direction is to enhance the pulse compression technique to concentrate into a single potential minimum the energy of all the monochromatic waves contained in a chirp pulse [47]. With all these technical developments and optimisations, one should be able to strongly improve the confinement of the moving QD and reach a level spacing of several meV that will be immune to unwanted Landau–Zener transitions to excited states during the SAW transport.

In parallel, the electrostatic potential should be made as uniform as possible along the transport channel by (i) optimising the connections between the different sections of the device and (ii) by suppressing the disorder potential induced by the random distribution of ionised dopants in usual GaAs heterostructures. For the second point, undoped structures should be used, at the expense of a more complicated gating architecture to accumulate a finite electron density where reservoirs are needed. Let us mention that undoped structures have already been implemented successfully in SAW experiments to create tunable p–n junctions for single-electron to single-photon conversion [54] opening prospects for single-spin detection by conversion into a single polarized photon.

Finally, SAW-assisted single electron transfer could be applied to the field of spin qubits in group IV semiconductors such as Si, Ge, and their compounds, since using SAWs instead of QD arrays could speed up the spin transfer between distant localised qubits. Since these materials are however not piezoelectric, the challenge will be to develop hybrid structures with a thin piezoelectric layer atop the qubit layer.

### Concluding remarks

While significant challenges lie ahead in terms of scalability, SAWs are very relevant in establishing rapid quantum links between electron spins stored within QDs integrated into complex quantum networks. They also hold promise in offering the first electronic flying qubit, with the next challenging task being to achieve uniform potential throughout the flight, in order to keep the electron in a well-defined quantum state.

At a more fundamental level, SAW-assisted transport of few-electron droplets offers the possibility to explore the partitioning statistics of multi-electron interacting systems. The single-shot measurement capability inherent in such experiments allows for the recording of a complete set of partitioning probabilities, which can be analysed using the full-counting-statistics formalism.

It is evident that the next decade will bring forth numerous intriguing findings and applications in single electron transport facilitated by SAWs.

## Acknowledgements

We acknowledge funding from the European Union H2020 research and innovation program under Grant Agreement No. 862683, ‘UltraFastNano’. C B acknowledges funding from the French Agence Nationale de la Recherche (ANR), Projects ANR QCONTROL ANR- 18-JSTQ-0001 and QUABS ANR-21-CE47-0013-01. C B and H S acknowledge funding from the Agence Nationale de la Recherche under the France 2030 programme, Reference ANR-22-PETQ-0012. S.T. acknowledges financial support from JSPS KAKENHI Grant Nos. 20H02559, 23H00257 and JST Moonshot R&D Grant No. JPMJMS226B.

## 4. Phononic quantum technologies with quantum emitters

### Daniel Wigger^1^, Tilmann Kuhn^2^ and Paweł Machnikowski^3^

^1^ Department of Physics, Wilhelm-Klemm-Str. 9, 48149 Münster, Germany

^2^ Institute of Solid State Theory, Wilhelm-Klemm-Str. 10, 48149 Münster, Germany

^3^ Institute of Theoretical Physics, Wrocław University of Science and Technology, Wybrzeże Stanisława Wyspiańskiego 27, 50–370 Wrocław, Poland

E-mail: d.wigger@uni-muenster.de, tilmann.kuhn@uni-muenster.de and Pawel.Machnikowski@pwr.edu.pl

### Status

**Figure 6.**
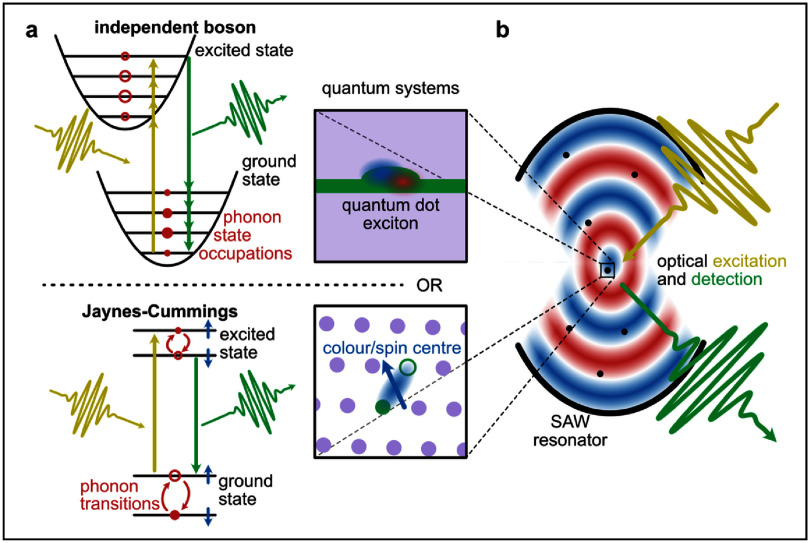
Schematic picture of the hybrid quantum platform integrating optically driven quantum emitters and quantum acoustics. (a) Schematics of the different theoretical models: quantum dots (top) require the independent boson model leading to the description of phonon sidebands, transitions between spin states (bottom) are described by the Jaynes–Cumming model. (b) Sketch of a SAW resonator and the optical driving and readout. The indicated emitters can be of quantum dot (top) or colour/spin centre type (bottom).

Quantum emitters acting as single photon sources are characterized by electronic states with an energy splitting in the visible or near-visible range. Due to the spin degree of freedom, these electronic states may be further split into spin multiplets with energy splittings in the *µ*eV–meV range. While the electronic splittings are much larger than typical phonon energies, thus prohibiting phonon-induced transitions, spin splittings are in the range of phonon energies and, depending on the selection rules, phonon-induced transitions may occur. Therefore, there are two conceptionally different coupling types between phonons and quantum emitters, depending on the considered transitions, as sketched in figure 6(a). On the one hand, the independent boson model (top) is applicable for any type of emitter as it describes how the crystal lattice reacts to local changes of the charge carrier density when switching between ground and excited states. As a consequence, the energy and lattice configuration are renormalised and phonon sidebands appear in the spectrum. The new hybrid eigenstate of excited state and deformed lattice is called polaron. The structure of the Hamiltonian ${H_{{\mathrm{IB}}}} = \left( {{b^\dagger } + b} \right)|\left. x \right\rangle \left\langle x \right.|$ is of pure-dephasing type, which means that it does not describe phonon-induced transitions between the electronic states.

Resonant phonon transitions are energetically possible between spin states (figure 6(a, bottom)), which appear in a particular well-resolved way in colour centres. Here the adequate description has the structure of the Jaynes–Cummings model ${H_{{\mathrm{JC}}}} = {b^\dagger }\left| { \downarrow \rangle \langle \uparrow \left| { + b} \right| \uparrow \rangle \langle \downarrow } \right|{\text{ }}$ with the eigenstates resembling exciton–polaritons from quantum optics which might be called spin-phoniton in this context [55]. In this situation, phonons can be used to coherently control the quantum state constituted by the spin states.

Analytic solutions to both models’ dynamics are only known in some special cases of optical driving, e.g. for continuous wave (cw) excitation, pulse excitations in the ultrashort pulse limit, or if we make approximations, like a classical description of the phonons. Therefore, applying and extending the models to simulate the performance of quantum technological devices remains an important part of modern research.

**Figure 7.**
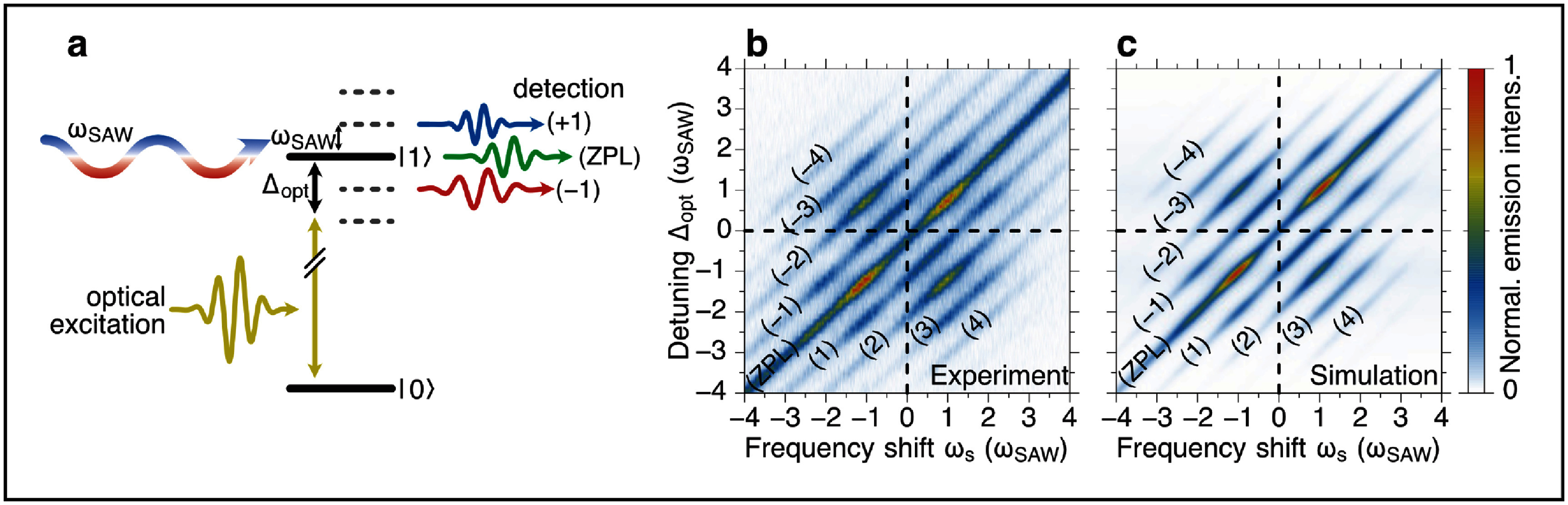
(a) Schematic of the experiment: frequency tuneable light is scattered from a two-level system (quantum dot exciton) which is modulated with a SAW. (b), (c) The resonance fluorescence spectra (*x*-axis) are plotted for each applied detuning ${{{\Delta }}_{{\mathrm{opt}}}}$ between exciton transition and driving cw laser (*y*-axis). (b) Experiment, (c) simulation. (b), (c) Reproduced from [56]. CC BY 4.0.

It was recently shown that the semiclassical version of the independent boson model, where the quantised phonon modes are replaced by monochromatic mean fields, modulating the transition energy of quantum emitters, are well suited to describe light scattering from single QDs, as schematically sketched in figure 7(a). This is exemplarily demonstrated by the cw laser detuning scan of the resonance fluorescence spectrum depicted in figures 7(b) and (c). The complex pattern of phonon sideband intensities in (b) is almost perfectly reproduced by the model in (c) [56]. In its full quantum form the generation of phonon quantum states and the dynamical formation of the polaron in a single QD during pulsed optical excitation was successfully explained by the independent boson model [57].

In the resonant phonon-coupling case, pulsed acoustic transitions between two spin states are routinely described in the semi-classical limit of the Jaynes–Cumming model, where the driving field operators are replaced by coherent amplitudes $b \to \beta \left( t \right)$ while the transition operators remain quantum. This Rabi-model-like treatment is usually sufficient to describe coherent control of spin states in colour centres by MW fields [58] which were recently moved to the acoustic domain [59]. Therefore, the same semiclassical description works for acoustic coherent control.

### Current and future challenges

As described in the following chapters, current and future challenges dealing with the interplay between quantum emitters and acoustics focus on the development of fully-fledged hybrid quantum platforms. From a theoretical perspective, this means that the mean field, i.e. the semi-classical approximations of the original full quantum models, cannot be applied anymore. These approximations consider interaction with coherent states including many phonons and have been successfully applied to traveling SAWs with large strain amplitudes. In contrast, using the full quantum model opens several further opportunities, e.g. by expanding the considered Hilbert space to hybrid quantum states taking the electronic/spin and phononic degrees of freedom into account.

One obvious challenge is the quantum state transduction between the optical and the acoustic domain, i.e. between photons and phonons. Dynamical control of both, the independent boson and the Jaynes–Cummings model offer a tremendous range of opportunities to imprint quantum features into the involved boson fields. Schrödinger cat states and squeezed states are prominent examples that are considered in quantum computing or sensing, respectively [60]. To provide useful guidance for future experimental realisations in this context, rigorous simulations of devices need to take the fundamental coupling mechanisms between photons, quantum emitters, and in particular phonons accurately into account. Here, the dynamics of the involved strain fields connected with the phonon excitations strongly depend on the considered material architecture. Not only the used material itself but the realisation of infrastructures like cavities and waveguides strongly influence the phonon mode structure and consequently the emitter-phonon coupling mechanisms.

Combining optical excitation and photon emission in the visible with acoustic phonons shows that we need to cover several orders of magnitude in time. The same holds for spatial dimensions, reaching from atomic scale colour centres to *µ*m-scaled acoustic metamaterials. This renders computational challenges in particular when full quantum models, that require descriptions in multi-dimensional Hilbert spaces, need to be linked with continuum simulations of the SAWs.

### Advances in science and technology to meet challenges

As mentioned, the successful semi-classical models applied to the interplay between optically driven quantum emitters and phonon fields need to be treated in their full quantum form to rigorously describe effects like quantum state transduction between the two domains. In this regard, initial steps have been made by simulating photon scattering spectra with the independent boson model [61] and even the readout of phonon statistics via the light scattering spectrum of a single quantum emitter has been demonstrated theoretically [62].

The sketch of a potential infrastructure that allows to interface the existing light-driven quantum emitters (QD-like and colour-centre-like) via SAW resonator phonons is shown in figure 6(b). There, we distinguish between QD-like emitters, that are described by the independent boson model (figure 6(a, top)) and colour-centre-like emitters with resonant spin textures, described by the Jaynes–Cummings model (figure 6(a, bottom)). Such a hybrid photon-emitter-phonon approach provides a large flexibility to form new eigenstates between parts of the entire systems depending on the coupling mechanisms and strengths between different parts of the system.

In the long run, the development of a quantum transducer between visible single photon quantum states and phonons in the GHz spectral range will require far reaching theoretical predictions of coupling efficiencies, structure designs, and conversion efficiencies to name a few.

In case of the description of phonon-driven transitions between spin states in colour centres, quantum optics has already paved the way towards single emitter and phonon operation. However, the details of the coupling mechanisms between colour centre spins and phonon fields requires dedication shown by the rather complex structure of the Hamiltonian describing the relevance of different types of strain [63], e.g. volumetric or shear strain.

Based on the Jaynes–Cummings-like coupling, first theoretical works have suggested phonon networks between remote spin centres in waveguides [64]. Here, optimised phononic waveguide and cavity designs require further effort in simulating these structures and characterising their mode structures for example by finite element methods [65]. One might even envision combining multiple quantum emitters of the same (QD-like or colour-centre-like) or of different type (QD-like and colour-centre-like) in a single phonon cavity and thereby create novel functionalities governed by the different coupling mechanisms between the emitters mediated by the common acoustic quantum mode.

### Concluding remarks

The vision of hybrid phonon-based quantum technologies is based on the combination of quantum optics for long-distant and quantum acoustics-based on-chip communication. Promising links between the two realms are quantum emitters, that interact strongly with photons and provide different coupling mechanisms to phonons. The latter are based on their internal energy structure. If a resonant coupling between spin states is not possible for phonons, only energy and lattice renormalisations are possible, described by the independent boson model. This is typically the case for emitters like QDs. If resonant spin transitions are possible for phonons, the Jaynes–Cummings model is applicable.

The growing possibilities to produce phononic infrastructures like waveguides and cavities and combine them with quantum emitters opens a wide range of opportunities to form hybrid functionalities including optical, electronic, spin, and acoustic excitations. To find efficient solutions for future problems posed by quantum technology, appropriate theoretical descriptions are imperative. These can be based on the combination of the two described quantum models and large-scale classical treating the continuum mechanics of phononic crystals architectures.

## Acknowledgements

Financial support by the Alexander von Humboldt foundation within a research group linkage grant between the groups in Münster and in Wrocław is gratefully acknowledged. P M acknowledges support from the National Science Centre (Poland) under Grant No. 2023/50/A/ST3/00511.

## 5. Quantum dot phononic devices

### Matthias Weiß^1^ and Galan Moody^2^

^1^ Physikalisches Institut, Universität Münster, Wilhelm-Klemm-Str. 10, 48149 Münster, Germany

^2^ Electrical and Computer Engineering Department, University of California, Santa Barbara, CA 93106, United States of America

E-mail: matthias.weiss@uni-muenster.de and moody@ucsb.edu

### Status

Single semiconductor quantum dots (QDs) represent the best-known source of on-demand single photons regarding the simultaneous optimisation of purity, indistinguishability, and brightness and thus constitute an important component of photonic quantum technologies. For such implementations, it is essential to have precise control of the QD’s spectral properties. This control can be achieved by utilising the dynamic strain field of a SAW, which periodically modulates the transition energies of semiconductor QDs. The use of mechanical waves has the advantage that they interact with virtually any solid-state system and can thus be utilized as a universal quantum link once the single-phonon level is achieved. Furthermore, SAWs can be efficiently coupled piezoelectrically to external MW circuits using IDTs, making this system ideal for the implementation of MW-to-optical transduction schemes in the gigahertz range.

**Figure 8.**
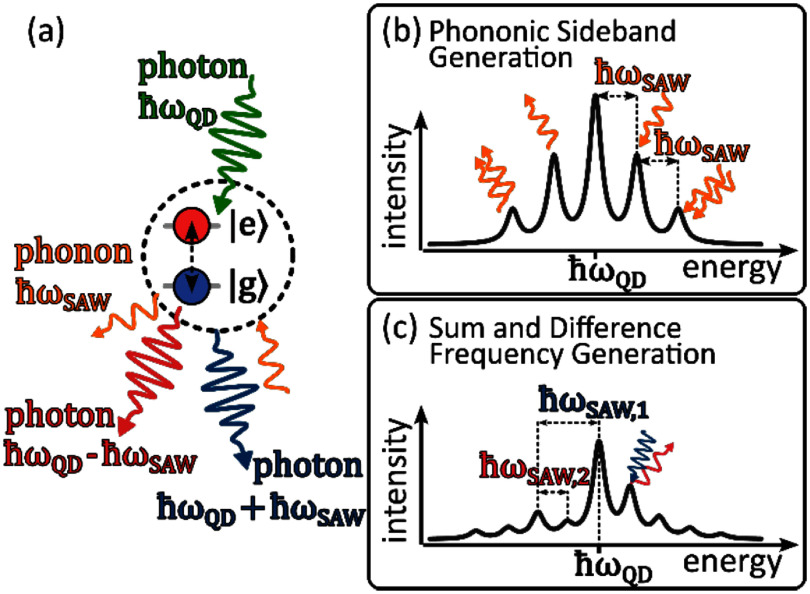
Resonance fluorescence on an acoustically modulated quantum dot (two level system). (a) Upon resonant scattering at a periodically modulated QD, photons can absorb phonons from the acoustic field or emit phonons into the acoustic field, resulting in a blue or red shift of the scattered photons. (b) Absorption and emission of a discrete number of phonons leads to the formation of equidistant phonon sidebands in the fluorescence spectrum. (c) When a QD is simultaneously modulated by two acoustic fields, the generation of sum and difference frequencies can be observed.

Recent experiments on the dynamic modulation of single QDs via acoustic fields were conducted in the resolved sideband regime, where the mechanical frequencies exceed the optical linewidth of the QD. This can be achieved by selecting sufficiently high acoustic frequencies or by optically driving the QD resonantly in the limit of small Rabi frequencies [66–68]. The mechanism of this scattering process is depicted in figure 8(a). Upon scattering on the two-level system (QD), photons can either absorb phonons from the acoustic field or emit phonons into the acoustic field, resulting in the formation of phonon sidebands (see figure 8(b)). For fully resonant optical excitation and modulation by a single acoustic wave phonon absorption and emission probabilities are balanced and the sidebands are symmetric with respect to $\hbar {\omega _{{\mathrm{QD}}}}$. These processes are well described within the independent boson model, as presented in section 4.

In addition to the observation of phononic sidebands, a series of pivotal experiments were conducted. For instance, parametric excitation was demonstrated, whereby the system was optically resonant and driven via one of the sidebands. In this scheme, the absorption or emission of phonons can be selectively favoured, enabling bi-directional energy conversion between the acoustic and optical domains (see section 4) [56, 67]. In addition, it was shown that by simultaneously modulating a QD with two acoustic fields of different frequencies, sum and difference frequency generation processes can be observed (see figure 8(c)). Because these processes depend on phase-matching conditions between the two fields, they can be used to deliberately enhance or suppress certain sidebands [66]. The robustness of these acoustic control schemes was theoretically confirmed, showing high fidelity scattering remains achievable under realistic noise conditions with coherent driving [69].

Furthermore, it was shown that using detuned optical pulses and coherent phonon driving, the QD can be prepared in an excited state preferentially through phonon-assisted processes, a mechanism useful for enhancing fidelity in MW-to-optical transduction [70].

In addition to implementing these new measurement and excitation techniques, considerable effort has been invested in integrating QDs into more complex mechanical systems with the objective of enhancing the coupling to mechanical fields. These systems include SAW resonators [67, 71], suspended QD membranes [68, 72], and QD membranes heterogeneously integrated into superconducting SAW resonators on strongly piezoelectric LiNbO_3_ [14], as well as nanowire QDs transferred onto thin-film LiNbO_₃_ and integrated into hybrid photonic waveguides [73].

### Current and future challenges

The primary objective of coupling individual QDs to mechanical fields is to reach high cooperativity and strong coupling in the single-phonon regime, which enables the transduction of quantum information from a single photon to a single phonon and vice versa [74]. To achieve this goal, several challenges must be addressed.

Firstly, the OM coupling strength ${g_0}$ between QDs and phonons must be significantly enhanced in order to overcome the intrinsic dissipation of both the QD ($\gamma $) and the mechanical system ($\kappa $). To achieve this, it is necessary to enhance the density of phononic states in the vicinity of the QD by localising the acoustic field within a mechanical resonator on length scales comparable to the acoustic wavelength (a few microns). Such an approach was recently demonstrated with focusing SAW cavities, resulting in >1 MHz single-phonon coupling rate ${g_0}$ [75]. While this is coupling is competitive with state-of-the-art approaches based on OM nanobeams and membranes [76], the large QD natural linewidth (∼100–500 MHz) and acoustic cavity mode linewidths on the order of 100 kHz make reaching the large single-phonon cooperativity and strong coupling regimes challenging.

Secondly, the resonance frequencies of these resonators should be as high as possible in order to reach the resolved sideband regime, and to ensure that the mean thermal phonon occupation is well below unity at temperatures accessible by state-of-the-art cryogenic systems. However, the resulting small mode volumes necessitates precise control of the QD position to ensure a maximum overlap between a the QD and the mechanical mode profile. This can be achieved by fabricating phononic structures with respect to predetermined QDs, or in the case of hetero-integrated devices, by a precise alignment of transferred QD membranes to prefabricated resonators. It has been demonstrated that such resonators can significantly enhance the OM coupling strength. However, when going to smaller mode volumes, scattering of phonons into the bulk crystal increases, limiting the quality factor of the resonator.

Lastly, to achieve MW-to-optical transduction, not only does the single-phonon OM coupling rate have to exceed the intrinsic loss rates of the QD and mechanical system at high frequencies, but the QD must be co-integrated into an acoustic and optical cavity to also enhance the optical efficiency. Co-integration with an optical cavity would enable efficient optical coherent control for pumping whilst enabling efficient photon collection to leverage the on-demand, deterministic nature of photon emission from QDs, which is a key distinguishing factor from OM resonators without emitters. Furthermore, nanofabrication processing creates etched surfaces and RF transmission lines near the QD that can result in undesirable charge noise. Controlling the QD charge state through passivation or integration in vertical diode structures is thus imperative for maintaining the desirable high-quality properties intrinsic to QDs.

### Advances in science and technology to meet challenges

**Figure 9.**
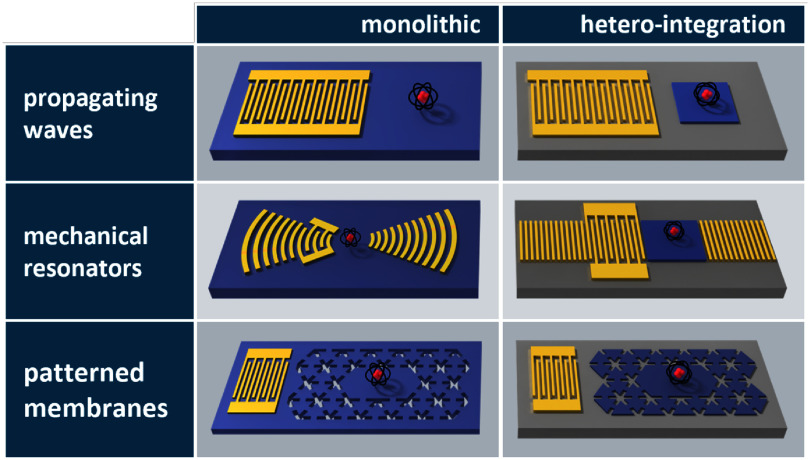
Possible implementations for dynamic modulation of QDs using acoustic phonon fields. These include dynamic modulation by a propagating SAW (top row), by the mechanical mode of a Bragg type resonator (centre row) and the modes of (suspended) phononic crystal resonators (bottom row). All these implementations can be done monolithically on the host substrate of the QDs (left column) or in hetero-integrated devices where QD membranes (blue) are transferred onto a different substrate (grey) (right column).

Many of the challenges discussed in the previous chapter can be resolved by designing phononic structures that are tailored to the specific scientific and technology requirements. Figure 9 provides an overview of the various possible structures for coupling QDs to acoustic fields, with each structure exhibiting its own advantages and disadvantages.

The most straightforward implementation of acoustic modulation of a QD is by the use of a propagating SAW. Here the amplitude, phase, and, when specifically adapted IDT designs are employed, the frequency of the acoustic wave can be fully controlled by external RF electronics. This makes this device an ideal test-bed for investigating the coupling of an acoustic field to a QD and for testing and implementing different excitation and detection schemes [56, 66]. However, due to relatively weak opto-mechanical coupling, propagating SAWs are not suited for fully fledged quantum applications. To confine the acoustic field to a smaller mode volume, a resonator can be defined by two distributed Bragg reflectors (DBRs). These reflectors can be realized by metal stripes or grooves etched into the substrate. Furthermore, the DBRs can be curved to focus the acoustic field and thus further reduce the mode volume. It has been demonstrated that such resonators can significantly enhance the OM coupling strength. However, reducing the mode volume typically leads to a concomitant increase in the scattering of phonons into the bulk modes, limiting the quality factor of the resonator. Bulk scattering can be mitigated by incorporating QDs into suspended membranes, which reduces or eliminates the bulk mode density of states entirely [56, 66, 72, 77]. These membranes can be further co-designed to obtain phononic and opto-mechanical crystals that allow for precise control and confinement of phonons and photons in such structures.

All the structures described here can be fabricated monolithically directly on the material system hosting the QDs, typically GaAs. Although GaAs is an excellent material for opto-mechanics, its weak piezoelectricity impedes the coupling of mechanical modes to external electrical circuits. This is of particular importance when superconducting qubits are to be coupled to mechanical modes, which is a prerequisite for MW-to-optical transduction. This can be circumvented through heterogeneous integration of QDs with, for example, other highly piezoelectric substrates such as lithium niobate (LN) [14, 78, 79]. Thin film LN on sapphire allows for both phononic and photonic waveguiding, and these acoustic and optical modes can be coupled to a QD in a transferred thin-film membrane as shown in figure 9. LN has a larger acoustic velocity compared to III–V materials, which results in stronger acoustic confinement in the transferred III–V membranes with slower sound velocity. In addition to the different approaches for OM coupling shown in figure 9, advances in fabrication are required to retain the excellent QD and OM resonator properties. When fabricating QD OM devices, surface passivation or co-integration with electrical tuning and control of QD charge and spin is important for ensuring radiatively limited linewidths, minimising spectral diffusion, and manipulation of the QD excitonic charge state and spin population.

### Concluding remarks

The coherent interaction between acoustic phonons and optically active semiconductor QDs has proven to be a highly promising platform for future quantum technologies. In such systems, the phonon, which can couple to virtually any solid-state system, serves as a universal link between solid-based qubits and QDs, and thus also to infrared photons.

Despite significant progress, challenges remain in achieving strong single-phonon cooperativity, minimising decoherence from charge noise, and precisely controlling QD-resonator alignment. Addressing these will require advances in nanofabrication, cryogenic integration, and materials engineering. In particular, the integration of QDs into phononically and photonically tailored environments will play a pivotal role in the transition of this system from the laboratory to practical applications. Furthermore, hetero-integration of QDs enables the combination of their advantageous optical properties with those of other material systems, such as the strong piezoelectricity of LN.

Looking ahead, acoustically driven QD systems offer a compelling route toward scalable quantum interfaces that connect MW, mechanical, and optical degrees of freedom. With continued development, such systems may play a central role in future quantum networks, transducers, and signal-processing platforms.

## Acknowledgements

G M acknowledges support via the UC Santa Barbara NSF Quantum Foundry funded via the Q-AMASE-i program under Award DMR-1906325. M W acknowledges funding by the Deutsche Forschungsgemeinschaft (DFG, German Research Foundation)—465136867.

## 6. Spin mechanics with defects

### Alberto Hernández-Mínguez^1^ and Snežana Lazić^2^

^1^ Paul-Drude-Institut für Festkörperelektronik, Leibniz-Institut im Forschungsverbund Berlin e.V., Hausvogteiplatz 5-7, 10117 Berlin, Germany

^2^ Departamento de Física de Materiales, Instituto Universitario de Ciencia de Materiales ‘Nicolás Cabrera’ (INC) and Condensed Matter Physics Center (IFIMAC), Universidad Autónoma de Madrid (UAM), 28049 Madrid, Spain

E-mail: hernandez-minguez@pdi-berlin.de and lazic.snezana@uam.es

### Status

Nanometre-scale structures consisting of charge carriers trapped within structural imperfections in the lattice of inorganic semiconductors have attracted great attention as versatile systems for quantum photonics and spintronics. These defect-bound quantum states behave as quasi-atoms capable of emitting non-classical light thereby providing qubits for the emerging photonic quantum technologies. Moreover, their localized electronic orbitals together with their electron spin magnitudes define a plenitude of energy levels which can be coupled not only via photons (e.g. optical and MW fields), but also via phonons, as already discussed in section 4. Since SAW-induced acousto-mechanical and acousto-electric modulation mechanisms typically extend over a thickness of approximately one acoustic wavelength (i.e. a few micrometres) both below and above the substrate surface, they provide a powerful method for ‘wireless’ and non-destructive dynamic reshaping of structural, optical, and electronic properties of the system.

**Figure 10.**
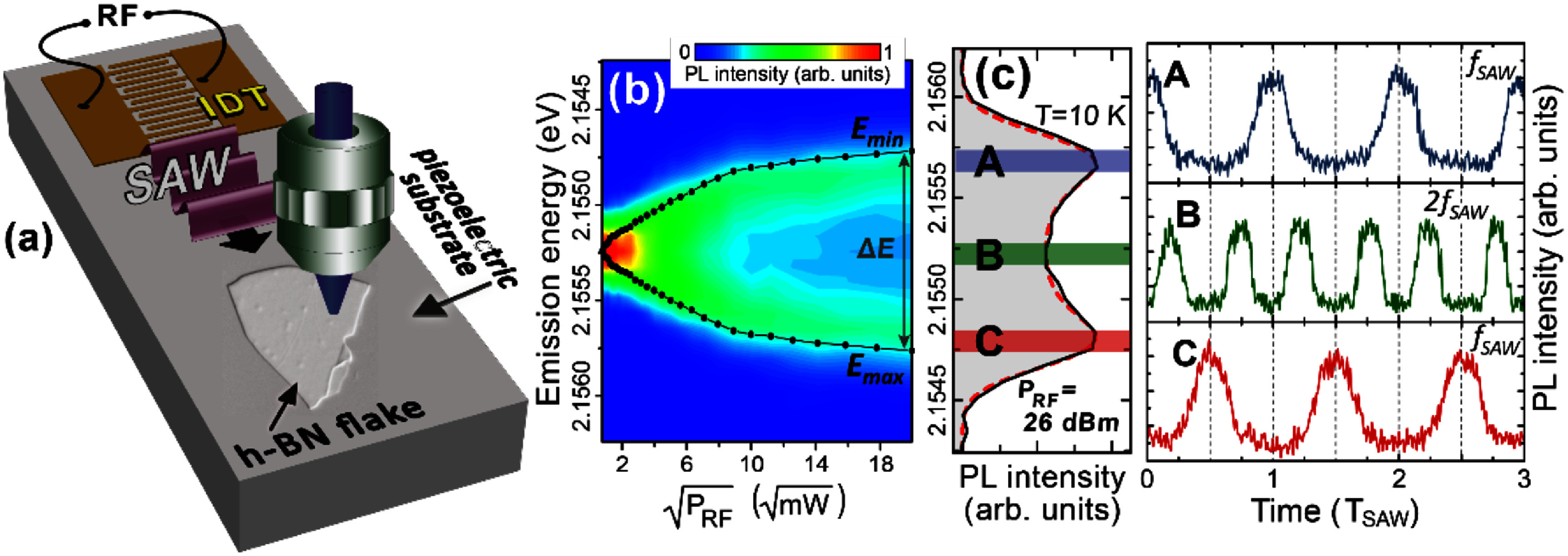
(a) Illustration of an acoustic experiment: A semiconducting material hosting quantum emitters is placed onto the SAW propagation path. The SAW is excited by applying a radio frequency (RF) signal to an interdigital transducer (IDT) patterned on a piezoelectric substrate. Dimensions are out of scale. (b) Acoustic tuning of a ZPL in h-BN, measured at 10 K. (c) Control of photon emission rate via acoustically mediated photon time-binning, measured at the maximum RF power applied to the IDT. The PL intensity scales of the time-resolved luminescence have been normalized for clarity. Reproduced from [81]. CC BY 4.0.

The tailoring of the electronic energy levels via SAW-driven oscillating Stark effect and/or elastic deformation requires luminescent defects with narrow zero-phonon lines (ZPLs). Their linewidth is primarily determined by the intrinsic lifetime of the excited electronic state and it is limited by interactions with the local environment such as thermal lattice vibrations. For this reason, the SAW modulation of the ZPL has been demonstrated at cryogenic temperatures in nitrogen-vacancy (NV) centres in diamond [80], defect centres in h-BN [81], as well as in a variety of 2D transition metal dichalcogenides [82]. Figure 10 shows the SAW-mediated spectral tuning of the ZPL for a single defect in h-BN, which has further been combined with spectral detection filtering for temporal control of the emitted photons [81]. In this way, both spectral tuning and on-demand single-photon emission are simultaneously achieved, thus providing an effective post-fabrication, *in-situ* tuning method for controlling and manipulating otherwise random properties of quantum light sources that severely limit practical applications in quantum photonics.

**Figure 11.**
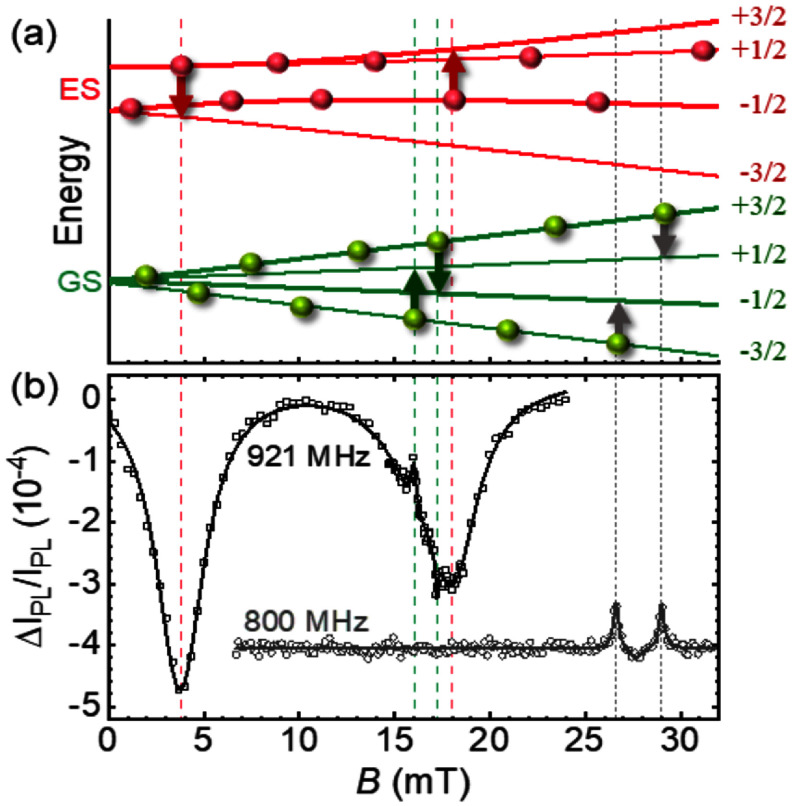
(a) Energy dependence of the ground (GS) and excited state (ES) 3/2-spin of the V_Si_ centre in an in-plane magnetic field, *B*. Green (red) vertical arrows denote SAW-driven GS (ES) spin transitions at 921 MHz. Grey arrows denote MW-driven spin transitions at 800 MHz. (c) Optically detected spin resonances driven by the SAW (black squares) and by the MW field (grey circles). Narrow (broad) resonances correspond to GS (ES) spin transitions. Reproduced from [86]. CC BY 4.0.

Regarding spin control, SAW-driven spin manipulation was first reported in diamond NV centres by exploiting the strong coupling of strain to their excited electronic states [83]. Ground state spin manipulation was then demonstrated in neutral silicon divacancies (VV) in 4H-SiC, where mechanically driven Autler-Townes splitting and magnetically forbidden Rabi oscillations were reported [84], and shortly afterwards in single SiV centres in diamond [59]. While all these spin centres require operation at cryogenic temperatures, the long spin coherence time of the negatively charged silicon vacancy (V_Si_) in 4H-SiC allows SAW-driven spin manipulation at room temperature [85]. In contrast to the 1-spin of the NV and VV centres, the 3/2-spin of the V_Si_ makes it possible to optically address magnetically forbidden spin transitions without the need of any additional MW field [85]. Moreover, by simultaneously driving spin transitions in the ground and excited states with the same SAW, see figure 11, novel phenomena such as the coherent trapping of the spin have been demonstrated [86].

### Current and future challenges

There are still several challenges to the use of spin-opto-mechanical processes in defect centres for quantum applications. First, all the proof of concepts discussed above have been realised using free-space optics. Large-scale quantum networks based on spin optomechanics will require the on-chip integration of both nanophotonic and nanophononic structures (see sections 10, 11 and 13) containing defects implanted at well-defined positions, similar to the QDs case discussed in section 5. Moreover, since SAWs can propagate over distances exceeding several millimetres with almost no dissipation, individual defects located at different positions within the acoustic path can be simultaneously and independently controlled by the same SAW field, thus allowing parallel schemes for quantum information processing. The deterministic integration of single NV centres with a SiO_2_/Si-based photonic platform has already been demonstrated [87], as well as the integration of V_Si_ centres into nanophotonic waveguides [88]. The next step should be the incorporation of nanophononic structures compatible with the nanophotonic ones for efficient acoustic manipulation.

So far, SAW-driven spin–phonon coupling in defect centres has been realised using strong, focused standing SAWs [59, 83,84,85]. However, the elliptically polarised strain fields of travelling SAWs can break time-reversal symmetry and thus excite chiral spin-acoustic resonances [85], which are essential for the realisation of unidirectional spin-OM networks. Spin control driven by chiral SAW fields requires the combination of spin centres with excellent optoelectronic properties and long coherence times, and piezoelectric materials with large electromechanical (EM) coupling coefficients for efficient SAW generation by acoustoelectric (AE) transducers, see section 14.

Finally, quantum devices based on the acoustic control of defects in 2D materials like h-BN are currently limited by the fact that flakes of these materials must be transferred to the surface of a piezoelectric substrate. This procedure severely limits scalability and is deleterious for the mechanical coupling between substrate and flake. Therefore, a future challenge will be the optimisation of the mechanical coupling at the interface between piezoelectric substrate and 2D material.

### Advances in science and technology to meet challenges

The monolithic integration of nanophotonic and nanophononic structures containing atom-like centres has recently been demonstrated for QDs in GaAs-based semiconductor platforms [89]. Similar realisations with defect centres will require the combination of their host materials (e.g. diamond or SiC) with insulating substrates exhibiting low refractive index for optical guiding and strong piezoelectricity for efficient SAW excitation. In addition, phonon–photon conversion mechanisms involving acoustic phonon sidebands, as discussed in sections 4 and 5, require acoustic frequencies exceeding the linewidths of the ZPLs, which are typically on the order of several GHz. The challenge here is that such SAW frequencies require AE transducers with dimensions at the limit of current fabrication technologies. These limitations can be overcome by using materials with higher sound velocities and stronger EM coupling coefficients, such as GaN, AlN and AlScN [90, 91], which have also recently been shown to host point defects with single-photon emission [92, 93]. Here, the main efforts will be to identify centres with non-zero spin [94] and to determine their crystal structure, energy levels, and coupling efficiency to the SAW fields. The realisation of such systems is beneficial because they are compatible with the upcoming 6G telecom technologies and can therefore be easily integrated.

With regard to defects in 2D materials, one possible strategy to enhance the mechanical coupling will be to avoid transfer processes by further developing the growth of 2D materials directly on substrates compatible with piezoelectric SAW excitation. An alternative approach could be to enhance the spin–phonon coupling by means of a third electronic excitation, such as magnons. SAW-driven spin waves (SWs) in the GHz frequency range can be efficiently excited in ferromagnetic thin films, see sections 8 and 9, and the coupling between magnons and the spin of NV centres in diamond has already been reported [95]. The scalable realisation of such magneto-acousto-optical coupling schemes in 2D materials will require the further development of material combinations exhibiting the appropriate mixture of magnetic, piezoelectric and optoelectronic properties.

### Concluding remarks

The proof-of-concept demonstrations showing coherent manipulation of several types of luminescent defects by SAW fields highlights the great potential and versatility of acoustically based control schemes for applications in quantum technologies. The next step should be the integration of these defect centres into hybrid nanophotonic and nanophononic platforms to combine the acoustic manipulation and optical addressing of the information with the transfer of such information between centres. These functionalities will require further advances in materials science and engineering to identify defects with excellent optoelectronic properties and long spin coherence times in host materials that are also compatible with on-chip photonic and phononic architectures.

## Acknowledgements

S L acknowledges financial support from the Spanish MICINN through a research project under Contract PID2020-113415RB-C21.

## 7. Gigahertz optomechanics with light–matter exciton–polaritons

### Alexander S Kuznetsov and Paulo V Santos

Paul-Drude-Institut für Festkörperelektronik, Leibniz-Institut im Forschungsverbund Berlin e. V. Hausvogteiplatz 5-7, 10117 Berlin, Germany

E-mail: kuznetsov@pdi-berlin.de and santos@pdi-berlin.de

### Status

**Figure 12.**
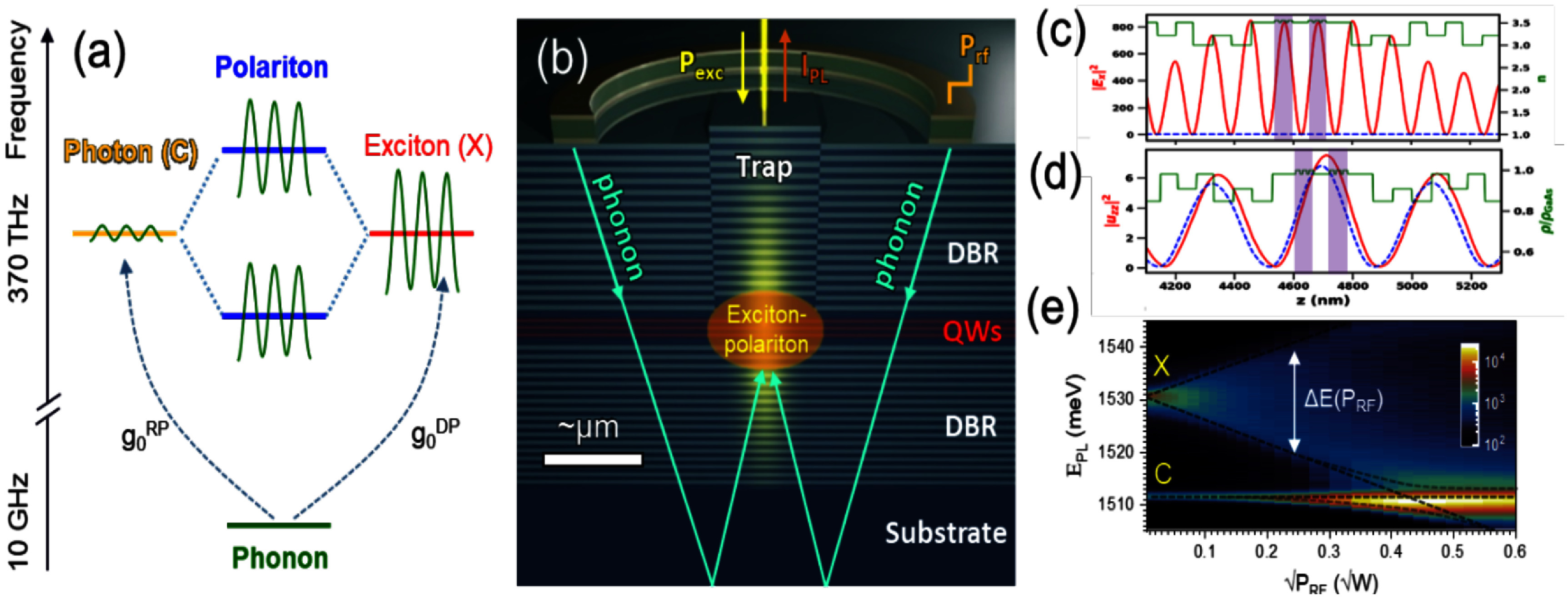
(a) Schematic energy diagram showing the polariton modes (in blue) emerging from the strong coupling between cavity photons and exciton resonances. A GHz phonon couples to the polariton via the radiation-pressure-like coupling and the deformation potential mechanism. The green wavy lines sketch phonon-induced energy modulation of the modes. (b) Sketch of the resonant cell in a structured microcavity (MC). A *μ*m-sized wider region created within the MC spacer forms a trap that laterally confines phonon and photon fields. Phonons in the form of longitudinal bulk acoustic waves are generated by RF-driving a ring-shaped bulk acoustic wave resonator on top of the MC. Phonons reach the centre of the trap via multiple reflections at the polished backside of the substrate. Simulated spatial distribution of the squared (c) optical field ${\left| {{E_x}} \right|^2}$ (at ∼1530 meV) and (d) phonon uniaxial strain ${\left| {{u_{zz}}} \right|^2}$ (at 7 GHz) around the MC spacer. The green lines in (c) and (d) show, respectively, the depth profiles of the refractive index (n) and the normalized mass density ($\rho /{\rho _{{\mathrm{GaAs}}}}$). The shaded regions designate the locations of GaAs QWs. Reproduced from [99]. CC BY 4.0. (e) Time-integrated photoluminescence colour map showing the evolution of the polariton resonances as a function of the RF power applied to the transducer at 10 K. The upper resonance (denoted as *X*) is exciton-like, while the lower one (denoted as *C*) is photon-like. The *X*-resonance is strongly modulated in energy via the deformation potential mechanism, with the energy modulation amplitude (Δ*E*) reaching 50 meV for the largest RF power. The dynamic anti-crossing around √*P*_RF_ ≈ 0.43 √*W* can be identified. The energy-modulation of the *C*-resonance is more than an order of magnitude smaller. Reproduced from [100]. CC BY 4.0. (c) An experimental photoluminescence spectrum of the emission of a confined polariton condensate at 10 K under acoustic modulation displaying well-resolved sidebands separated by the LBAW energy *ħω*_LBAW_.

The coherent coupling between light and mechanical motion is at the heart of optomechanics (OM) [96] and its applications, e.g. in photonics, quantum systems, as well as in GHz-to-optical conversion. Conventionally, OM setups are electronically passive with an external light source and an OM coupling based on the cavity-enhanced radiation pressure (RP) mechanism. A relatively new paradigm with promising prospects relies on the exciton-enhanced coupling to high-frequency (10–300 GHz) acoustic phonons in light–matter systems, where excitons and photons hybridize to form exciton–polaritons [97]. While the original idea has been discussed for superlattices of quantum wells (QWs) [98], there has been rapid progress enabled by the development of hybrid microcavities (MCs) for polaritons and GHz phonons conceptualized in figures 12(a) and (b). Such a hybrid MC consists of DBRs designed to co-localize and enhance the optical and acoustic fields within a thin spacer layer containing QWs. The latter are positioned close to the anti-nodes of the acoustic and optical modes to enhance the coupling to QW excitons.

The phonon–photon interaction becomes significantly enhanced because the phonon strain couples to exciton resonances via the efficient deformation potential (DP) mechanism (i.e. the strain-induced bandgap modulation), which benefits from the large values of the DP constants of semiconductors (of approx. 10 eV), thus implying in polariton energy shifts on the meV scale even for strain on the order of 10^−4^. One particularly attractive material platform is the (Al,Ga)As material system, where, due to the double ‘magic’ coincidence between photons and acoustic phonons [101], an (Al,Ga)As MC for near infra-red photons also co-localizes 20 GHz phonon modes, cf figures 12(c) and (d), yielding optical and acoustic quality factors reaching *Q* ≈ 10^4^ at 10 K. The strong light–matter coupling plays a crucial role in preserving high optical *Q*’s, which would otherwise suffer from the presence of highly absorbing QWs. It has recently been demonstrated that the (single-polariton) DP coupling strength can reach *g*_0_ = 20 MHz [102]: the later exceeds the effective RP one (due to the modulation of the refractive index and spacer interfaces) by several orders of magnitude and has led to the demonstration of sensing of attometre displacement [103]. The hybrid light–matter nature of polaritons also induces strong non-linearities and polariton lasing characterized by the sub-GHz optical linewidths. In combination with the large g_0_ values, very large multi-polariton cooperativities *C* ≈ 10^4^ can be achieved [100].

Another useful aspect of the MC platform is that its spacer region can be patterned on the *μ*m-lateral-scale to create intracavity traps zero-dimensionally confining both phonon and polariton states. Finally, monochromatic longitudinal acoustic phonons with frequencies up to 20 GHz can be injected into the spacer by a radio-frequency-driven piezoelectric BAW resonator (BAWR) fabricated on the sample surface. This has enabled the demonstration of GHz-modulation of polariton resonances [104] with energy modulation amplitudes reaching Δ*E* ≈ 50 meV [102], cf figure 12(e), which exceeds by an order of magnitude the typical light–matter coupling energy of approx. 3 meV.

Optical experiments on intra-cavity traps have revealed rich dynamic phenomena including self-driven oscillations/lasing [100], synchronisation [105], and spontaneous breaking of the time translation symmetry leading to time-crystals [106]. Furthermore, the piezoelectric phonon injection enabled the demonstration of polariton-based coherent optomechanics as well as strong polariton–phonon OM coupling [21]. These results consolidated a new area of GHz optomechanics with polaritons, termed polaromechanics [99]. Table 1 compares relevant the following parameters of various OM systems: typical mechanical (optical) frequencies ${v_{\mathrm{M}}}{ }$ (${v_{\mathrm{C}}}$) and dissipation rates ${\gamma _{\mathrm{M}}}$ (${\gamma _{\mathrm{C}}}$), single photon coupling rate (${g_0}$) and cooperativity (${C_0}$) and the type of the platform. In the case of the polariton platform, the two values for ${\gamma _{\mathrm{C}}}$ and ${C_0}$ correspond to the ones in the single-polariton (left) and polariton non-equilibrium condensate regimes (right).

**Table 1.** Properties of different optomechanical platforms. Reproduced from [100]. CC BY 4.0.

Platform	*ν*_M_ (GHz)	γ_M_ (Hz)	*ν*_C_ (THz)	γ_C_ (Hz)	*g*_0_ (Hz)	*C*_0_	Type
This work	7-200	1 × 10^6^	370	50 × 10^9^, 0.5 × 10^9^(+)	20 × 10^6^	0.02, 3.2	Active
Bulk Fabry–Perot [107]	12.7	86 × 10^3^	193	73 × 10^6^	24	4 × 10^−10^	Passive
Nanobeam [108]	3.7	3.5 × 10^4^	195	5 × 10^8^	9 × 10^5^	0.18	Passive
2D crystal cavity [109]	10.2	8.5(+)	192	5 × 10^8^	1.1 × 10^6^	∼1	Passive
QD in SAW cavity [110]	3.6	2 × 10^5^	324	1.6 × 10^9^	1.2 × 10^6^	0.018	Active
Ring resonator [111]	0.314	1.01 × 10^5^	196	∼1.3 × 10^10^	3.7 × 10^4^	1.6 × 10^−4^	Passive

### Current and future challenges

Exploitation of the previously outlined polaromechanical phenomena requires a polariton decoherence rate (*γ*_pol_) much smaller than the mechanical frequency (*ω*_M_), i.e. *γ*_pol_ ≪ *ω*_M_. In the few-polariton regime (i.e. for low optical excitation fluencies), *γ*_pol_ is determined by the cavity photon lifetime (typically, 5–10 ps) yielding *γ*_pol_/2*π* ≈ 50 GHz. So far, the fast modulation regime has only been achieved in the MC system by exploiting the long coherence time (2*π*/*γ*_pol_) of polaritons in the condensate. The latter arises from a balance between laser-induced particle creation and losses by photon escape from the MC and, thus, depends on the experiment conditions. Experimentally, decoherence rates down to *γ*_pol-BEC_/2π = 50 MHz have been reported for polariton condensate [112]. Presently, however, the upper limit for the coherence is unknown. Understanding the mechanisms limiting condensate coherence is, therefore, crucial for the field of polaromechanics and its potential applications. The main challenge will be to achieve linewidths *γ*_pol_ ≪ *ω*_M_ in the single-polariton regime, a key prerequisite for the quantum microwave-to-optical conversion using these particles, which requires cavity photon lifetime exceeding 150 ps, or *ω*_M_/2*π* > 50 GHz. The second challenge will be to reach above unit quantum cooperativity, *C*^q^ = *C*_0_/*n*_th_ ⩾ 1, which is paramount for quantum interactions between the polariton and mechanical modes, with a small thermal population of the mechanical mode *n*_th_ ∼ 1. The later presupposes *ħω*_M_ > *k*_B_*T*, which for 20 GHz phonons means operating below 1 K. It has been suggested in Zambon *et al* [113] that polaromechanical single-polariton cooperativity *C*_0_ could reach unit value. However, experimentally it is well below that: *C*_0_ ≈ 0.02 [102].

While considerable progress has been made towards the design of optimized BAWRs [114], the efficient electrical generation of phonons at frequencies above 20 GHz remains a technological challenge. For example, BAWRs for the 20 GHz range normally require very thin piezoelectric films (e.g. 80–100 nm-thick textured films for ZnO-based or AlN-based resonators), which must be well-textured and free of pinholes. Going beyond that limit requires new strategies and materials. Furthermore, these resonators generate predominantly longitudinal bulk acoustic phonons, which couple weakly to the spin degrees of freedom of polariton condensates. The efficient excitation of transverse or even chiral GHz acoustic waves (vortices) is an emerging field with important consequences also for other fields related to opto-acoustics [115].

Finally, most of the polaromechanical studies have so far been restricted to the (Al,Ga)As materials platform. One of the bigger application barriers for this materials platform lies in the very low operation temperature of about 10 K, which is intimately related to the low binding energy (and, thus, temperature stability) of excitons in GaAs as well as the increased phonon absorption at elevated temperatures. Progress has been made to extend the operation temperature for this platform [107]. As an example, the microwave-to-photon transduction in the many-particle regime can be carried out at temperatures of at least 77 K (liquid nitrogen), where cooling can be provided by inexpensive Sterling cryo-coolers. The temperature limitation of the (Al,Ga)As material platform provides a strong motivation to explore other material systems more suitable for room temperature operation.

### Advances in science and technology to meet challenges

The resolved sideband condition *γ*_pol_ ≪ *ω*_M_ can be fulfilled in (Al,Ga)As MCs in the few polariton regime by either employing ultra-high-*Q* MCs (*Q* > 10^6^) [108] or using phonon modes above 50 GHz. The first approach requires ultra-pure molecular beam epitaxy (MBE) reactors for sample growth with minimized optical absorption. The second approach can be realized with current MCs by properly designing the structure to optimize the coupling to these high frequency phonons. This approach requires piezoelectric excitation of tens of GHz phonons. In principle, this can be achieved by exciting overtones of the BWARs, albeit at a reduced acoustic amplitude. Alternatively, the generation of phonons above 20 GHz can be done using ps-long optical pulses. In order to reach *C*_0_ ⩾ 1, further enhancements of *g*_0_ and reduction of the *γ*_pol_ are required, which can potentially be realized by using ultra-high-*Q* MCs and polariton traps with dimensions below 1 *μ*m.

Increasing the generation frequency and reducing the size of bulk transducers are key developments towards the quantum polaromechanics in the single-polariton regime and realisation of on-chip arrays of single-particle microwave-to-optical converters. Here, transducers based on AlN (also ScAlN), which have higher acoustic velocity as compared to presently-employed ZnO films, could enable devices operating well above 20 GHz. Remarkably, the piezoelectric generation of acoustic waves up to 60 GHz has been demonstrated using Lamb waves in thin-film LiNbO_3_ resonators [109]. Finally, the presently used BAWRs have opaque metal contacts: they need to be made into a ring-shaped aperture for optical access to the polaromechanical cell at its centre, see figure 12(b). The acoustic amplitude then becomes limited by the multiple round trips through the MC and the substrate. The process is restricted to very low temperatures due to the acoustic absorption. One envisaged solution is to develop transducers based on transparent conductors, e.g. indium-tin oxide films.

Another anticipated development is the utilisation of QW materials with high exciton binding energies to enable strong photon–exciton coupling and condensation at room temperature. While the MBE process used to grow (Al,Ga)As MCs provides the best quality samples, it is restrictive in terms of material palette. A hybrid approach combining the MBE-grown lower DBR and spacer with QWs (or half cavity) with the deposition of the upper DBR using another process (e.g. sputtering) can be envisaged. The hybrid fabrication enables the use of materials with higher exciton binding energies (e.g. (Al,N)Ga alloys), non-crystalline (e.g. organic) semiconductors and 2D materials (e.g. transition-metal dichalcogenides (TMDCs)). Interestingly, it has recently been demonstrated that mesoporous materials can sustain GHz phonons [110]. Therefore, fabrication of DBRs based on such mesoporous layers could enable new types of compact on-chip hybrid opto-acousto-piezoelectric sensors.

### Concluding remarks

Polaromechanics is a new exciting area, which bridges GHz optomechanics and light–matter exciton–polaritons in hybrid phonon–photon semiconductor MCs. On the one hand, this emergent area enriches the field of GHz optomechanics by bringing intrinsic light sources and the exciton-boosted coupling to phonons and ns-long coherence time of polariton condensates. On the other hand, it enables the application of coherent interactions with GHz phonons as a tool to control light–matter interactions. So far, the work has been focused on understanding the basic physical and technological aspects of single or few coupled resonant cells defined in patterned (Al,Ga)As MCs. The natural next step will be to realize Floquet engineering (and Floquet topological states) in lattices of many coupled cells [111, 116]. In terms of polariton–phonon coupling, the polaromechanical platform compares favourably with most passive OM platforms, which makes it attractive to demonstrate microwave-to-optical conversion. The intrinsic scalability of the patterned MC platform is a key property towards polaromechanical chips. Several challenges, however, need to be overcome on the way towards applications, e.g. in polariton lasers, quantum converters, as well as for the extension of the polaromechanics concepts to QDs and 2D materials. New concepts are emerging including the condensation of THz-frequency vibrational modes interacting with polaritons [117] as well as optomechanics with disk resonators [118].

## Acknowledgements

We acknowledge the funding from German DFG Grants 359162958 and 426728819.

## 8. Nonreciprocal magnetoacoustics

### Matthias Küß^1^, Manfred Albrecht^1^ and Mathias Weiler^2^

^1^ Institute of Physics, University of Augsburg, 86135 Augsburg, Germany

^2^ Fachbereich Physik and Landesforschungszentrum OPTIMAS, Rheinland-Pfälzische Technische Universität Kaiserslautern-Landau, 67663 Kaiserslautern, Germany

E-mail: matthias.kuess@physik.uni-augsburg.de, manfred.albrecht@uni-a.de and weiler@physik.uni-kl.de

### Status

Nonreciprocal MW devices, such as isolators and circulators, are key components of modern communication technology. Isolators protect radio frequency (RF) devices from unwanted back reflections while circulators are three-port devices that channel signals exclusively in one circular direction, facilitating simultaneous transmission and reception at the same frequency. Isolators and circulators are also employed for low-noise qubit readout in superconducting quantum computers.

These nonreciprocal RF devices are based on the time-reversal symmetry breaking by the magnetisation of ferrite materials. Their typical dimensions are in the order of centimetres, corresponding to the RF wavelength at their operating frequency in the GHz regime.

**Figure 13.**
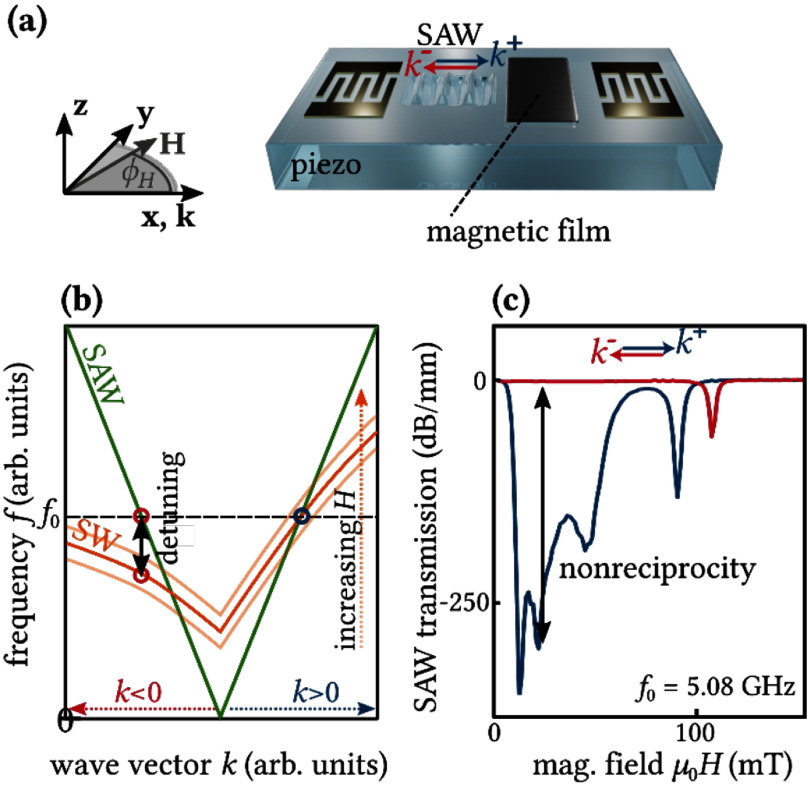
(a) Illustration of the investigated nonreciprocal piezoelectric/ferromagnetic hybrid. The SAWs can resonantly excite SWs in the magnetic film by magnetoacoustic interaction. (b) A large nonreciprocal SAW transmission can arise in magnetic systems with a nonreciprocal SW dispersion [$f\left( k \right) \ne f\left( { - k} \right)$] since resonant SAW-SW interaction is only possible for waves propagating in one direction (e.g. $k &gt; 0$), where the counter-propagating wave frequencies are detuned ($k &lt; 0$). The SW dispersion can be manipulated by an external magnetic field $H$. (c) The background-corrected SAW transmission of a CoFeB/Ru/CoFeB synthetic antiferromagnetic film shows a large nonreciprocity. Reprinted with permission from [119]. Copyright (2023) American Chemical Society.

Because of the greatly reduced wavelengths of SAWs compared to MW photons, SAWs are commercially used in miniaturized reciprocal MW components. The SAW lattice dynamics can interact with magnetic degrees of freedom by transfer of linear and angular momentum. The propagation of SAWs in magnetic media is therefore a promising avenue for developing a novel category of nonreciprocal RF devices, including acoustic isolators or circulators. A prototypical piezoelectric/magnetic hybrid device is illustrated in figure 13(a).

Nonreciprocal SAW transmission in piezoelectric/magnetic hybrids was initially observed in ZnO on yttrium iron garnet (YIG) crystals and later in thin nickel films on LiNbO_3_ [120]. The nonreciprocity was traced back to a directional SAW to spin wave (SW) coupling mechanism that is based on the relative helicities of SAWs and SWs. From a fundamental point of view, the magnetoacoustic wave is chiral and can be nonreciprocal because of a combined breaking of time- and space-reversal symmetry. In later experiments, it was found that the nonreciprocity is significantly larger than expected from pure magnetoelastic coupling [121, 122]. This was explained by the additionally present magneto-rotation coupling mechanism.

Another promising strategy for nonreciprocal SAW transmission in piezoelectric/magnetic hybrids is based on magnetic thin films with a nonreciprocal SW dispersion [123]. As depicted in figure 13(b), resonant SAW-SW interaction at the frequency *f*_0_ is only possible for waves with a wave vector *k >* 0 resulting in a pronounced SAW nonreciprocity. Magnetic thin films with interfacial Dzyaloshinskii–Moriya interaction [121, 122] and magnetic bilayers [124] have so far been discussed. Due to its large tuneability and large nonreciprocal SW dispersion, synthetic antiferromagnets (SAFs), which are made from two antiparallel coupled ferromagnetic layers and an ultrathin nonmagnetic spacer layer, are especially promising [123]. For a CoFeB/Ru/CoFeB SAF, a large nonreciprocal SAW transmission of more than 250 dB per mm length of the SAF along the SAW propagation direction was demonstrated [119], as shown in figure 13(c).

### Current and future challenges

**Figure 14.**
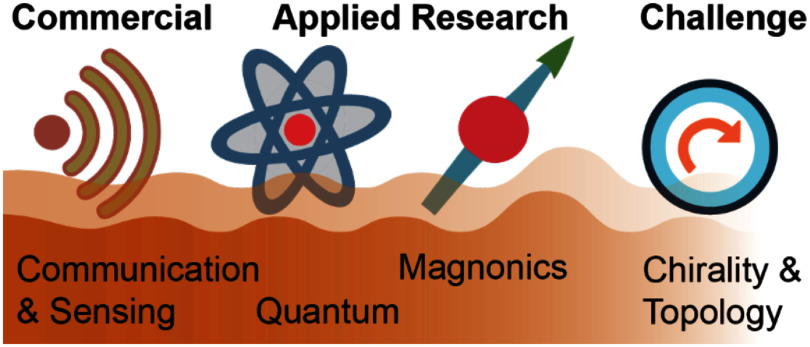
SAWs are commercially used for communication and sensing. Applied research in the condensed matter community includes the study of quantum effects and hybrid systems such as combining phononics and magnonics in magnetic media. Emerging challenges include finding ways to exploit the impact of chirality and topology in novel SAW-based devices.

There are several challenges that need to be addressed in the field of magnetoacoustics, as summarized in figure 14. On the technological side, to obtain commercially viable miniaturized RF isolators, the insertion loss needs to be brought on par with the widely available reciprocal SAW devices that are presently used. The current research-grade magnetoacoustic nonreciprocal devices described above are absorptive and thus fundamentally limited in power handling and efficiency. The magnetoelastic coupling results not only in SAWs with nonreciprocal amplitudes but also nonreciprocal phase shifts. Careful design of SAF magnetic layers should allow for low almost reciprocal SAW attenuation and large nonreciprocal SAW phase shift of *π* [125], which could pave the way to nonreciprocal SAW phase shifters and circulators. However, experimental verification of corresponding devices is so far missing.

On the more fundamental research side, we face the challenge of integrating magnetoacoustic elements into quantum-mechanical systems. The interest in addressing this challenge stems from the fact that SAWs can be controlled and sensed in the quantum regime [29]. If strong coupling of SAWs and SWs can be established in suitable systems, this would potentially open an avenue for the quantum-control of magnons. In this regard, the ideal magnetic materials should exhibit a large magnetoelastic coupling, low effective magnetic damping, and reasonable large saturation magnetisation and can be deposited in arbitrary thicknesses on diverse piezoelectric crystals. Another key fundamental challenge that should be addressed is the potential exploitation of SAW chirality for topological properties such as backscattering-free unidirectional SAW propagation. By taking advantage of the symmetry and chirality of SAWs in magnetic media, hybrid systems that combine phononics with photonics and magnonics may be established.

### Advances in science and technology to meet challenges

The operational frequency of nonreciprocal magnetoacoustic devices is currently restricted to the single-digit GHz range since the typical frequency ranges for SAWs (MHz to few GHz) and SWs (above 1 GHz) differ slightly. Because the nonreciprocal effects are in addition larger at higher frequencies, reaching a nonreciprocity contrast close to 100% is more difficult at low frequencies. This challenge could be addressed by using magnetic systems with tailored low frequency and low linewidth SW resonances. Moreover, the nonreciprocal magnetoacoustic SAW devices demonstrated so far are bandwidth-limited to a relatively narrow frequency range, defined by the slope of the wave dispersions and resonance linewidth. This restriction results in limitations, such as a limited operation band, and can be relieved by wide-band matching of the SAW and SW dispersions [123, 126] and appropriate IDT design. Novel approaches that allow for efficient SAW excitation over a broad frequency range would be needed for applications that require large RF bandwidths. This will require efforts to transfer established engineering knowledge from the field of commercial SAW devices to research-grade magnetoacoustic devices.

Going beyond the established magnetoelastic ferromagnets, such as CoFeB, FeCoSiB, and FeGaB, further improvements in material engineering are expected to leverage great potential for optimisation. In particular, hybrids that combine quantum acoustics and magnonics at mK temperatures will require suitable material combinations that allow on-chip integration of magnetic thin films and superconducting circuitry on piezoelectric substrates.

To further improve the coupling strength between SAWs and SWs [127], the electrical excitation and detection of SAWs in suitable low-damping magnetic media such as YIG needs to be established at GHz frequencies. Detection of single hybrid magnon–phonon excitations may be enabled by integrating ultrasensitive and nanoscale electromagnetic sensors, such as nitrogen vacancies in diamond, with magnetoacoustic devices. Full access to quantum magnetoacoustics will furthermore require operation at millikelvin temperatures.

### Concluding remarks

The addition of the magnetic degree of freedom and the concomitant breaking of time-reversal symmetry has opened a broad range of novel SAW-based functionalities. The field of nonreciprocal magnetoacoustics is moving towards active control and exploitation of chirality and topology, integration of quantum acoustics, and magnetic systems with strong coupling of SAWs and SWs. A better understanding of the underlying physical phenomena will potentially enable novel functionalities that combine the advantages of magnetoacoustics and quantum acoustics with the miniaturisation and flexibility offered by the established SAW platform.

## Acknowledgements

All authors acknowledge financial support by the German Research Foundation (DFG) via Project No. 492421737. M W acknowledges financial support by the European Research Council (ERC) via Grant No. 101044526 and DFG via TRR 173-268565370 (project B13).

## 9. Surface acoustic waves strongly coupled to spin waves

### Jorge Puebla^1,3^, Yunyoung Hwang^2,1^ and Yoshichika Otani^2,1^

^1^ Center for Emergent Matter Science, RIKEN, Wako 351–0198, Japan

^2^ Institute for Solid State Physics, University of Tokyo, Kashiwa 277-8581, Japan

^3^ Department of Electronic Science and Engineering, Kyoto University, Kyoto 615-8510, Japan

E-mail: puebla.jorge.8m@kyoto-u.ac.jp, ynyh24@gmail.com and yotani@issp.u-tokyo.ac.jp

### Status

The interaction between SAWs and spin waves (SWs) is gaining attention in spintronics and magnetism research [128]. SWs in ferromagnets, usually excited by MW photons, can also be triggered by phonons, as predicted by Kittel [129]. When SAWs and SWs reach strong coupling, their interaction surpasses the relaxation rates of both phonons and SWs. This leads to the formation of hybridized quasiparticles, combining features of both phonons and magnons [127]. Our recent experiments demonstrated this hybridisation at room temperature. We used acoustic wave reflectors to create an acoustic cavity, reducing phonon losses, and employed CoFeB layers with low magnetic damping. Additionally, our setup enhanced the coupling of SWs with a horizontal shear strain component [130, 131].

**Figure 15.**
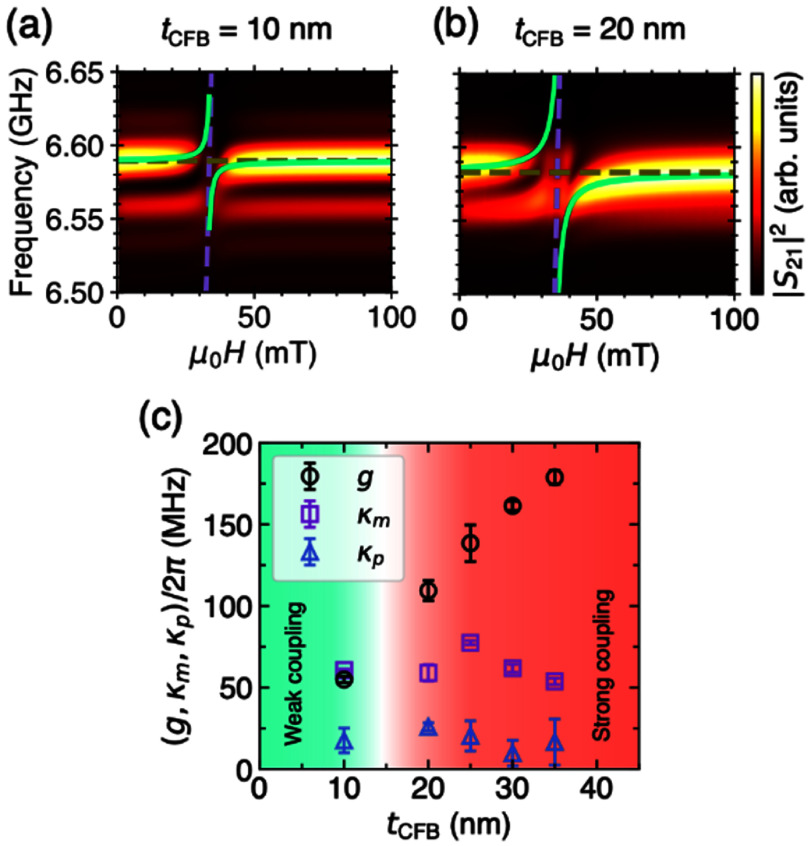
SAW transmissions when the external magnetic field is applied in the direction of SAW propagation; ${\phi _H} = {0^0}$. SAW transmission signal (${\left| {{S_{21}}} \right|^2}$) of the sample with CoFeB thickness of (a) ${t_{{\mathrm{CFB}}}} = 10$ nm and (b) ${t_{{\mathrm{CFB}}}} = 20$ nm under various amplitudes of the magnetic field ${\mu _0}H$. Purple and brown dashed curves in (a) and (b), indicate the calculated magnon (SWR) and phonon dispersions, respectively. The light green curve is the anticrossing calculation. For details on the theoretical model see ref [127]. (c) The coupling strength (*g*, the black circles) and magnon (${\kappa _m}$, the purple square) and phonon (${\kappa _p}$, the blue triangle) relaxation rates as a function of the thickness of the CoFeB layer. Reprinted (figure) with permission from [127], Copyright (2024) by the American Physical Society.

We investigated the avoided crossing or anticrossing between magnon and phonon dispersions as a strong coupling signature. In our experiments, SAW transmission data revealed a bending in the phonon dispersion when the externally applied magnetic field approached magnon resonance (see figure 15). With a CoFeB layer thickness of 10 nm, the phonon dispersion exhibited energy absorption at resonance around 30 mT, maintaining a constant and linear behaviour. However, when the thickness increased to 20 nm, the phonon dispersion not only absorbed energy at resonance but also showed noticeable bending, indicating strong magnon–phonon coupling. This coupling implies that phonons now exhibit magnon-like characteristics and can be influenced by magnetic fields, and vice versa, with implications for hybrid wave information and communication devices. The dashed curves in figure 15 represent calculated magnon (purple) and phonon (brown) dispersions, with the light green curve indicating anticrossing. Additionally, in the thin film limit where the acoustic wavelength exceeds the magnetic layer thickness, we observed a monotonic increase in coupling strength with CoFeB layer thickness (figure 15(c)), providing a route to achieve ultra-strong coupling.

### Current and future challenges

The anticipated shared properties of the hybrid magnon–phonon waves are indeed intriguing and require experimental verification. In magnonics, some outstanding challenges include relatively short propagation lengths and low propagation velocities. For ferromagnets, these parameters typically are the propagation length of a few micrometres, and the propagation velocity of a few tens of meters per second. In contrast, phonons exhibit longer propagation lengths (up to millimetres) and higher propagation velocities (in the range of thousands of meters per second). It is imperative to characterize the propagation length and velocity of the hybrid magnon–phonon waves. However, this task is not straightforward, particularly when SAWs couple with thin magnetic layers. SAWs excite multiple SWs as they traverse the magnetic layer, making it challenging to isolate a particular wave packet to study propagation lengths and velocities. Achieving this requires experimental schemes involving precise timing for excitation and detection. Coherence is another essential property of information and communication technologies. In the case of hybridized magnon–phonon waves, it is desirable to develop experimental techniques for reading out the phase without disturbing its coherence.

During the transition from weak to strong coupling regimes, the concept of the Purcell effect [132] also becomes relevant. This effect demonstrates that modifications in the density of the final states of an atom inside a resonant cavity can enhance spontaneous emission. An acoustic cavity device may induce a Purcell effect in the magnon–phonon excitations, potentially leading to lasing. The conditions under which this lasing can occur and how it may manifest in our hybrid magnon–phonon quasiparticles remain uncertain, but parallels to cavity electromagnetism provide grounds for optimism.

Beyond the strong coupling regime, increasing the magnetic volume of our hybrid devices could push us toward the ultra-strong coupling regime. In this regime, the coupling strength *g* is compared not with the energy losses in the system but with the excitation frequency ω, such that delineates the ultra-strong coupling regime. From a purely fundamental physics perspective, the ultra-strong coupling regime offers fertile ground and deserves a separate research category [133].

### Advances in science and technology to meet challenges

To address the challenges in studying hybrid magnon–phonon waves, we require experimental techniques capable of precisely assessing wave coherence with high spatial, temporal, and frequency resolutions. Recent advancements in time-resolved magneto-optical Kerr effect (t-MOKE) and micro-Brillouin light scattering (BLS) spectroscopy offer promising avenues in this regard.

An intriguing possibility lies in the emergence and rapid development of nitrogen vacancy (NV) centre magnetometry. NV centre magnetometry can potentially outperform t-MOKE and BLS in phase sensitivity. Bertelli *et al* [134] successfully demonstrated phase-sensitive magnetic imaging of coherent SWs by utilising a diamond chip with an ensemble of NV centres over the magnetic sample of interest. This phase resolution in the GHz frequency range, coupled with high spatial resolution, makes NV centre magnetometry highly attractive for characterising the coherence lengths and times of our hybridized magnon–phonon waves. Further developments in phase resolution with fast temporal detection can enable the study of phase coherence at the Rabi splitting of the magnon–phonon quasiparticles.

Ongoing developments of all-electronic MW detection schemes with high phase and frequency resolution [135] are also crucial for envisioned technology implementations of information processing circuits based on hybridized magnon–phonon waves.

Further improvements in magnon–phonon coupling strength can be achieved by employing magnetic materials with ultralow magnetic damping, such as YIG. However, a challenge lies in growing high-quality YIG thin films on top of piezoelectric substrates. A recent report demonstrated the epitaxial growth of multi-domain YIG film with low magnetic damping on top of a LiNbO_3_ substrate [136], and a more recent report demonstrated nonreciprocal resonant SAW absorption in an on-chip YIG-SAW device integrated using the focused ion beam technique [137].

Although the magnetic damping in our hybridized magnon–phonon wave experiments is currently larger than the phonon losses, improvements can still be made on the phonon side. Our acoustic cavity devices currently support two main phonon modes, potentially leading to undesirable crosstalk. Selectivity of a specific single phonon mode in the form of SAWs can be achieved using phononic crystals [138]. Engineering periodic potentials for SAWs not only results in mode selectivity but also induces nonreciprocal behaviours [139].

### Concluding remarks

We have provided a brief overview of the recent experimental demonstration of SAW hybridisation with SWs in the strong coupling regime, highlighting the anticipated implications for information and communication technologies. We anticipate that with advancements in experimental techniques offering enhanced phase, spatial, and temporal resolutions, the intriguing characteristics of this novel hybridized magnon–phonon wave will be unveiled in the short to medium term. Particularly intriguing would be the transition from the strong to the ultra-strong coupling regimes, and a transition from the classical to the quantum realm, most likely at ultra-low temperatures.

## Acknowledgements

We would like to acknowledge support of the Grants-in-Aid for Scientific Research (S) (No. 19H05629). J P acknowledges support of JSPS KAKENHI No. 24K00576 from MEXT, Japan.

## 10. Piezoelectric phononic integrated circuits^43^43An expanded version of the ideas outlined in this article was published in [146].

### Krishna C Balram

Quantum Engineering Technology Labs and Department of Electrical and Electronic Engineering, University of Bristol, Bristol BS8 1UB, United Kingdom

E-mail: Krishna.coimbatorebalram@bristol.ac.uk

### Status

Piezoelectric microresonators underpin modern wireless communication [140]. Despite billions of these devices being in widespread use everyday across a range of materials, device geometries and operation frequencies [141], every piezoelectric device in current commercial use shares one common characteristic: they all manipulate quasi-plane waves of sound, i.e. the transverse extent of the waves is large (10–20x) in comparison with the acoustic wavelength. The ideas of waveguiding sound waves (using slow-on-fast layer stacks) have been around for decades [142], but have not become mainstream due to a variety of challenges discussed in the next section. On the other hand, there has been a convergence of developments in the past decade [76] which makes it interesting to consider again the question of strong confinement and waveguiding of GHz acoustic waves in chipscale platforms. These developments include: the spectacular success of (silicon) integrated photonics in revolutionising telecommunications by exploiting strong confinement of light in low-loss platforms; hardware advances in quantum computing platforms (especially superconducting qubits) which opens up the need for networking remote systems [143] and the associated realizsation that acoustic waves can function as universal quantum transducers [144]; increased understanding of dissipation in nanomechanical systems and the development of techniques to mitigate the same culminating in nanomechanical systems with fQ (frequency quality factor product) figures of merit that far exceed bulk geometries [145]; and finally advances in semiconductor crystal growth (especially interface defect minimisation) driven by power electronics and high power MW amplifiers.

**Figure 16.**
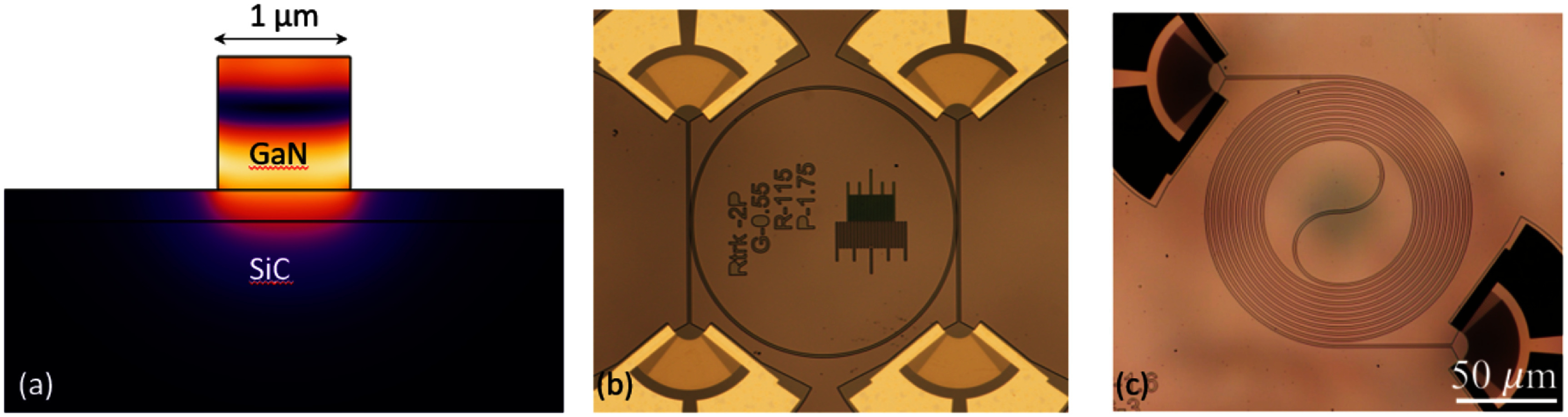
(a) Guided acoustic mode propagating in a 1 *µ*m wide gallium nitride waveguide on a silicon carbide substrate (b) Acoustic microring resonators with quality factors >10^4^ at 3.4 GHz (c) spiral delay lines with on-chip delays ∼2.5 *µ*s. © 2024 IEEE. Reprinted, with permission, from [22].

While the culmination of these trends makes it attractive to reconsider exploiting strong confinement of GHz acoustic waves in low-loss chipscale platforms, it is imperative to point out here that one really needs to move beyond replicating existing functionality in small form-factors. This is because the performance metrics for RF filters [141] are quite prohibitive and guided wave devices face some unique challenges in meeting them as discussed below. Therefore, the focus of this article is on effects that one can uniquely exploit in chipscale platforms [146]. These can be broadly classified according to whether the devices exploit the *low-loss* that chipscale guided wave platforms provide to realize high *Q* whispering gallery mode resonators and long spiral delay lines [22] (cf figure 16); *strong confinement* to realize sensors [147] that exploit the high perturbation sensitivity of guide wave platforms; or *field enhancement* to realize nonlinear effects such as four wave mixing and parametric amplification [148], acoustoelectric interactions [149] and efficient interfacing with nanoscale quantum systems [150].

### Current and future challenges

As alluded to above, the advantages of guided acoustic wave devices have been recognized from the very early days of ultrasonics research [142]. The main challenge that has prevented widespread adoption of these ideas are the excess insertion loss encountered when engineering guided wave systems. This insertion loss has two main components: *coupling loss* from transducers to waveguides and *propagation loss* within the waveguide itself. Waveguided platforms require efficient injection of acoustic waves from a transducer to a waveguide. Given impedance matching constraints [143], this boils down to a focusing problem of efficiently focusing acoustic waves to diffraction limited spot sizes and efficiently coupling this focused spot into an acoustic waveguide. In contrast to optical waves, the problem in the acoustic case is more complex as the slow acoustic waves are in general surface waves which makes implementing traditional (optical) focusing geometries like tapers inefficient. In addition, the acoustic waveguide system (for instance, a simple strip waveguide as shown in figure 16(a)) is in general a multimode platform and every interface (and surface roughness) provides a route for mode conversion. While these challenges are significant, there is no fundamental reason why acoustic fields cannot be injected into and out of wavelength scale cavities with near unity efficiency.

While the coupling loss is the main source of insertion loss in current devices, propagation loss is also critical. While whispering gallery mode acoustic wave resonators have shown record high *Q* factors [22], it is still unclear what the fundamental limit on acoustic dissipation is for waveguided geometries. A major challenge here is the lack of acoustic imaging modalities that can provide fast 3D acoustic field distributions. Without local imaging of acoustic wave scattering [151] and mode conversion, it is hard to optimize device performance based on only MW transmission measurements.

A final hurdle for widespread adoption of guided wave acoustic devices is the lack of dynamic control [152] on propagating acoustic fields in waveguide geometries. Dynamic control is necessary for active switching, routing and tuning of phononic integrated circuits. Traditional techniques that work reasonably well with bulk devices like thermal and electric field tuning need to be adapted to mesh well with the reduced footprints that guided wave platforms offer. More importantly, the device geometry (and material stack) needs to be carefully optimized to ensure dynamic control without incurring excess insertion loss and scattering.

### Advances in science and technology to meet challenges

To address the challenges in the previous section, one can broadly organize the advances need on three fronts: materials, devices and metrology. Given that strong acoustic confinement requires a slow-on-fast layer structure, the choice of fast substrates is limited to sapphire, silicon carbide and diamond. Given the need for strong acoustic confinement across all mode shapes and at all in-plane waveguide orientations, SiC [153] presents the best near-term choice accounting for acoustic velocities and commercial availability. This opens up the requirement for strongly piezoelectric crystalline thin films like LN and lithium tantalate (LT) [154] on SiC using wafer bonding and smart-cut [155]. In parallel, optimising the deposition of sputtered piezoelectric films like ScAlN [156] on SiC needs to be done to provide a viable near-term alternative to crystalline thin films for proof-of-principle devices. Waveguided devices require exquisite control of sidewall angles and surface roughness. A lot of work has already been done on optimising integrated photonic devices in LN and LT and many of these ideas can be ported over to acoustics. A viable alternative is to build on the developments in high power RF devices and exploit the gallium nitride on silicon carbide platform [22]. While the piezoelectric coefficient of GaN is lower than that of ScAlN, LN and LT, it is a piezoelectric semiconductor and provides some unique advantages in terms of engineering AE interactions in guided wave geometries [157].

In parallel, device geometries (both on the transducer and waveguide front) need to be optimized to reduce the excess insertion loss that usually accompanies guided wave devices. In particular, efficient unidirectional focusing transducers need to be developed which can efficiently focus GHz acoustic fields to *µ*m-scale dimensions. In parallel, phononic bandgap structures need to be optimized to ensure unwanted mode conversion at surfaces can be avoided. State-of-the-art guided wave devices currently have insertion loss exceeding 10 dB, even for short waveguide spans. This needs to be below 2 dB for these platforms to be attractive from an applications perspective.

Finally, metrology methods that can locally map acoustic wave scattering and mode conversion with nm spatial and ns temporal resolution need to be developed to understand the effects of nanofabrication on limiting acoustic loss. New metrology methods [158] that are sensitive to surface states need to be explored to understand the effects of surface dissipation in these geometries. Given the high surface to volume ratio of these devices, surface loss presents the ultimate limit on achievable performance and passivation techniques need to be developed similar to photonics.

### Concluding remarks

Strong transverse confinement of GHz acoustic fields in low-loss chip scale platforms provides a new degree of freedom that can be exploited to improve the performance of piezoelectric devices across a range of applications ranging from wireless communications to sensing to quantum information processing. To realize these benefits and to have an impact comparable to that of silicon integrated photonics on optical telecommunications, a number of challenges need to be addressed, with the two key ones being the excess insertion loss that usually accompanies guided wave geometries and the development of methods to exert effective dynamic control on propagating sound waves in chip-scale platforms.

## Acknowledgements

I would like to thank the members of my research group, in particular Mahmut Bicer, Stefano Valle, Fahad Malik, Jacob Brown and Ankur Khurana, for all their contributions to this research program over the years. I also gratefully acknowledge funding support from the UK’s Engineering and Physical Sciences Research Council (EP/V005286/1) and the European Research Council (758843).

## 11. Integrated acousto-optomechanics (IAOs)

### I-Tung Chen^1^, Keji Lai^3^ and Mo Li^1, 2^

^1^Department of Electrical and Computer Engineering, University of Washington, Seattle, WA 98195, United States of America

^2^Department of Physics, University of Washington, Seattle, WA 98195, United States of America

^3^Department of Physics, The University of Texas at Austin, Austin, TX 78712, United States of America

E-mail: itungc@uw.edu, kejilai@physics.utexas.edu and moli96@uw.edu

### Status

IAOs is a new paradigm of acousto-optics (AOs), in which acoustic wave devices and photonic devices are integrated on the same platform to engineer the OM interaction between light and sound. In these IAO systems, optical and acoustic waves co-propagate and interact within planar waveguide structures, enabling enhanced AO interactions compared to bulk counterparts due to the co-confinement.

IAO systems typically integrate photonic components such as waveguides, ring resonators, and Mach–Zehnder interferometers (MZIs) with EM transducers on piezoelectric materials, such as IDTs for generating SAWs and parallel plate transducers for generating BAWs. Commonly used piezoelectric materials in IAO systems (figures 18(a)–(e)) are LN, aluminium nitride (AlN), gallium nitride (GaN), zinc oxide (ZnO), and aluminium scandium nitride (AlScN). For a material to be efficient in AO interaction, it needs to have a high optical refractive index, low acoustic velocity, and a large photoelastic effect. These can be gauged with the AO figure of merit ${M_2} = {n^6}{p^2}/\rho {v^3}$, where $n$ is the refractive index, $p$ is the photoelastic constant, $\rho $ is the material density, and $v$ is the acoustic velocity. Figure 18(F) lists the ${M_2}$ values of selected AO materials. However, due to the high modal confinement in IAO, the moving boundary effects often dominate the photoelastic effect. In addition, to reduce acoustic loss, free-standing IAO devices are often fabricated to minimize such losses, making the boundary effect more prominent.

**Figure 17.**
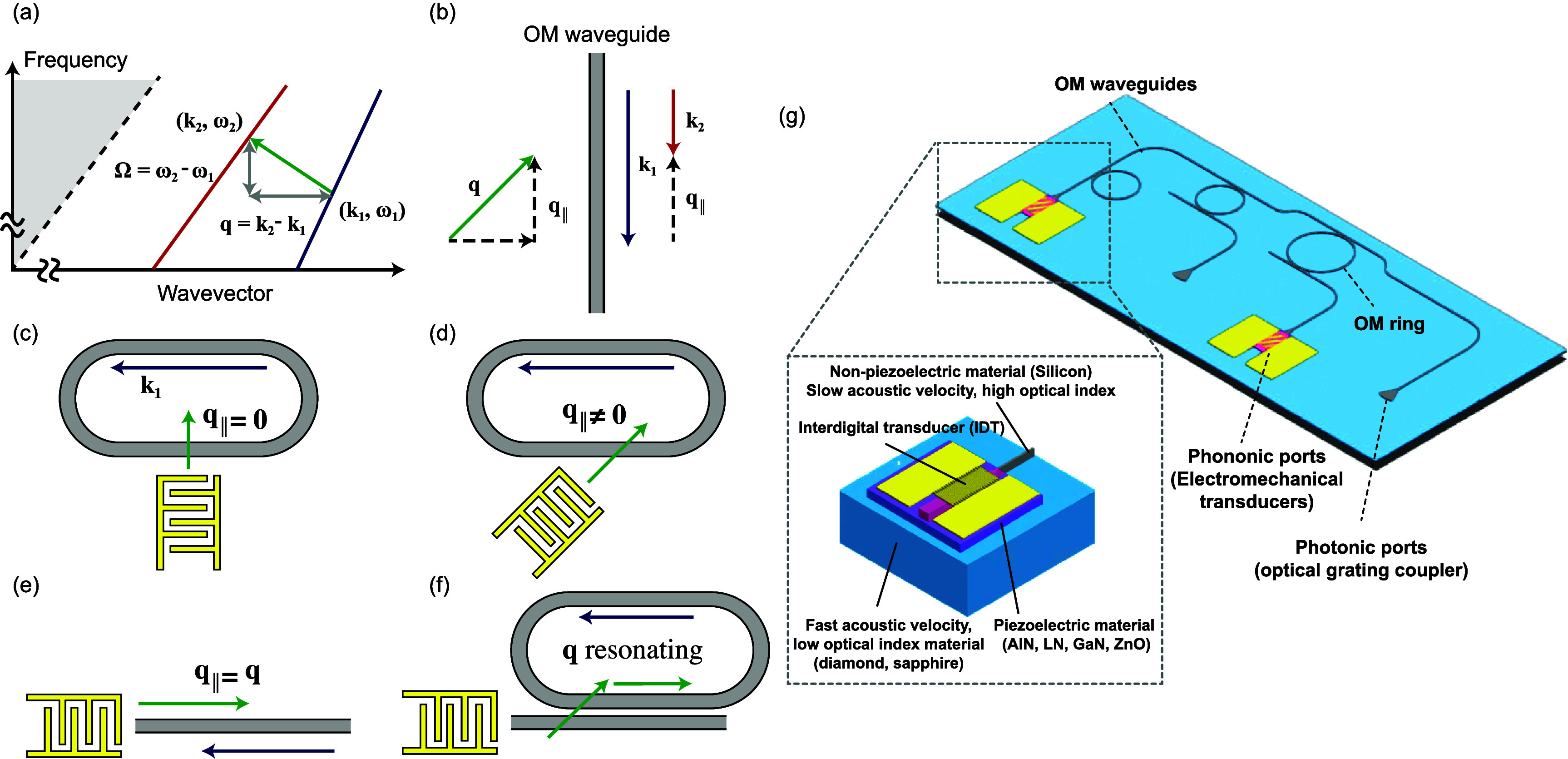
The principle intermodal scattering of guided optical modes and various types of integrated acousto-optomechanical device architecture. (a) Principle of inter-modal anti-stokes scattering in a waveguide. (b) The phase-matching diagram of an optomechanical waveguide. (c), (d) Categorisation of different acousto-optomechanical interaction by the value of parallel acoustic wavevector ${q_\parallel }$. (g) A schematic of an integrated acousto-optomechanical (IAO) device. Electromechanical transducers that are fabricated on piezoelectric material in which the acoustic and optical wave co-propagate. The optomechanical (OM) waveguides simultaneously guide acoustic and optical waves to OM ring resonators where the acousto-optomechanical interaction is enhanced.

Various IAO device architectures have been explored to enhance AO interactions. High-order acoustic Rayleigh modes have been used to modulate AlN photonic ring resonators [159], while suspended MZI and racetrack cavity integrated with IDTs have facilitated MW-to-optical conversion using LN [160]. These works are waveguides running perpendicular to the acoustic waves (figure 17(c)), which leads to a lower photon conversion efficiency <0.1%[160]. Sohn *et al* [161] tilted the IDTs relative to the waveguide to generate a parallel acoustic wavevector ${q_\parallel }$, which breaks the reciprocity of light propagation (with fixed acoustic direction) and realizes a magnetic-free optical isolator (figure 17(d)) and achieves an optical mode conversion efficiency of 17%. To push for even higher AO modulation strength, Liu *et al* [162] realized an OM waveguide enabling backward AO Brillouin scattering through a large ${q_\parallel }$ (figure 17(e)). Sarabalis *et al* [163] demonstrated an inter-modal Brillouin scattering (figure 17(a)) in an OM waveguide with co- or counter-propagating acoustic waves. An impressive near 100% optical mode conversion efficiency was also achieved with an OM waveguide by Zhang *et al* [164]. More recently, Chen *et al* [165] developed an OM ring resonator (OMR) in which acoustic and optical waves are co-confined and co-resonant, leveraging the large coupling strength of cavity optomechanics (figure 17(f)).

### Current and future challenges

*AO modulation efficiency and bandwidth.*

**Figure 18.**
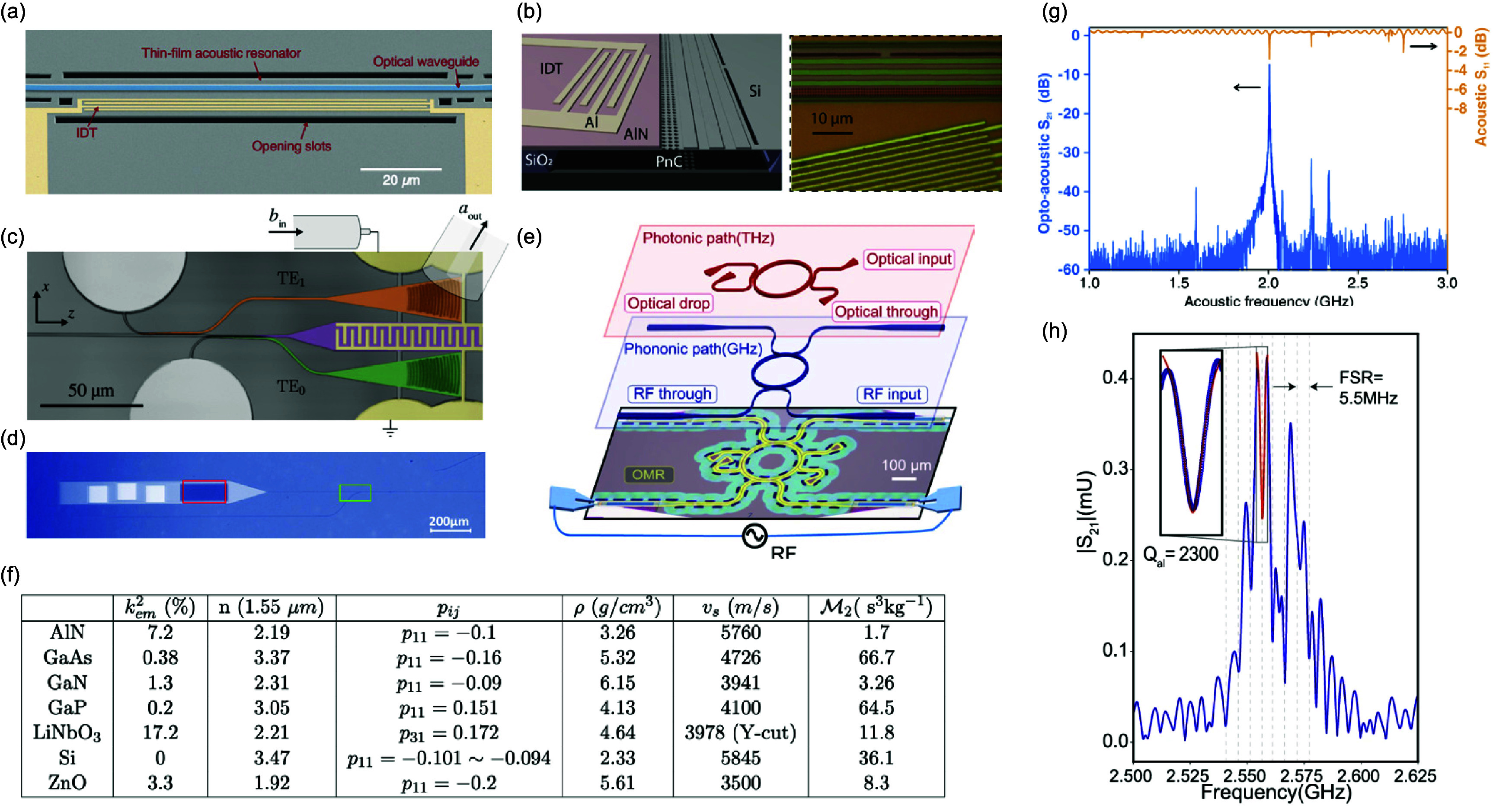
Representative integrated acousto-optomechanical (IAO) systems with different parallel acoustic wavevector ${q_\parallel }$ (a) A standing acoustic wave cavity integrated with suspended lithium niobate (LN) waveguides. Reproduced from [160]. CC BY 4.0. (b) A tilted interdigital transducer (IDT) on aluminium nitride (AlN) integrated with spiral silicon waveguides. Reproduced from [166]. CC BY 4.0. (c) A suspended LN optomechanical waveguide with co-traveling acoustic and optical waves. Reproduced from [163]. CC BY 4.0. (d) A non-suspended gallium nitride (GaN)-on-sapphire optomechanical waveguide. Reprinted with permission from [165] © The Optical Society. (e) A suspended gallium phosphide (GaP) optomechanical ring resonator (OMR) with co-resonating acoustic and optical modes. Reproduced from [164]. CC BY 4.0. (f) Acousto-optic figure-of-merit ${M_2}$ of selected materials. The electromechanical coupling coefficient $k_{{\mathrm{em}}}^{\mathrm{2}}$ is related to the ratio of mechanical power generated by RF driving power. Here the ${M_2}$ is calculated at telecom wavelength 1.55 $\mu {\mathrm{m}}$, and the definition is described in the main text. (g) A representative microwave-to-optical conversion efficiency measurement data that shows the microwave transmission coefficient (S21) measured from a high-speed photodetector. Reproduced from [160]. CC BY 4.0. (h) A microwave transmission spectrum, which shows the acoustic transmission power coefficient between a pair of IDT from a GaP optomechanical ring resonator. The extracted acoustic propagation loss is around 6 dB mm^−1^. Reproduced from [164]. CC BY 4.0.

A key challenge lies in enhancing the AO modulation efficiency, which is limited by two factors: (1) the EM efficiency to generate acoustic waves from RF driving power, and (2) the OM coupling strength, which determines the AO modulation efficiency. For optimal EM efficiency, the IDT must be impedance matched to the power source (50 ${{\Omega }}$). This can be achieved by designing the number of IDT finger pairs, N, and the aperture size. However, increasing N reduces the bandwidth, presenting an efficiency-bandwidth trade-off that needs to be considered carefully based on the applications. A representative MW photon to optical photon conversion efficiency measurement data is shown in figure 18(g). New transducer design ideas are needed to overcome this limitation. Conversely, the OM coupling strength depends on the confinement of modes and the interaction length. A solution to reduce the acoustic loss is to undercut the active area and suspend the thin film to eliminate the acoustic loss channel through the substrates. One such example using free-standing gallium phosphide (GaP) shows an acoustic propagation loss at 6.2 dB mm^−1^ (acoustic quality factor *Q* = 2,300) (figure 18(g)). However, this method reduces the device’s power handling capability, due to weakened heat dissipation, and less mechanical robustness.

*Scalability, fabrication challenges, and device footprint.*

The IAO modulation efficiency has been limited to a few percent until Zhang *et al* [164] recently showed a complete optical mode conversion on a non-suspended GaN-on-sapphire platform. The device is a 3 mm long OM waveguide with IDT driven at 0.56 W. The impressive near-unity conversion efficiency is achieved with the high RF driving power and long interaction length. The challenge here is to scale up the number of high-power, large footprint devices on chip to truly leverage the benefit of an integrated platform. To date, a variety of materials and substrates have been used in IAO devices, requiring complicated fabrication processes and making compatibility and integration with prevailing platforms such as superconducting qubits and silicon photonics a challenge. In addition, the thermal energy generated by the IDT could cause unintentional optical phase shift or resonance drifting in integrated photonic circuits, which is not ideal. A compact, power efficient solution is needed.

*Materials innovations.*

The intrinsic material properties play important roles in the AO figure of merit. Therefore, the selection of material may have different requirements based on the application. For high-efficiency, high-power devices in the classical applications (e.g. optical frequency shifter, isolator, beam-deflector), the power handling of the device is a challenge for the IAO since the high energy density in the IDT may damage the piezoelectric thin film. For quantum applications, the integration to a superconducting qubit using a high-overtone BAWR using AlN has been demonstrated, which shows a single phonon coherence time >10 $\mu {\mathrm{s}}$ [167]. On the other hand, for the MW-to-optical transduction, the challenge is that the stray electromagnetic field from the strongly piezoelectric material can cause noise and degrade the coherence of the quantum system, such as superconducting qubits. In addition to the noise from the MW photons, the thermal noise from the MW phonons at can also be reduced by phononic bandgap engineering using phononic shields [166].

### Advances in science and technology to meet challenges

*Direct acoustic imaging.*

Engineering the acoustic and optical modes to achieve optimal AO modal overlap is crucial to increase OM coupling strength. However, the excited acoustic mode profile in a complex device structure can differ considerably from the predictions by simulations such as finite element analysis. For example, direct visualisation of gigahertz acoustic waves using a novel transmission-mode MW impedance microscopy (TMIM) technique [168] has revealed the unintentional generation of high-order acoustic waves in a suspended waveguide, which reduces the AO modulation efficiency. TMIM can help identify the origin of acoustic wave scattering in devices and provide invaluable insights for better waveguide geometry design. Further advancement of TMIM in a cryogenic environment will also deepen the understanding of quantum acoustic and cryogenic AO modulations.

*Miniaturisation of the IAO devices.*

Miniaturising IAO devices is crucial for compatibility and scalability with integrated photonic circuits, yet it remains a significant challenge. Confining the acoustic mode in acoustic waveguides and cavities can enhance the interaction and reduce the device footprint by shortening the needed interaction length. For applications requiring strong OM coupling strength but only a small bandwidth (e.g. MW-to-optical transduction or filtering), an IAO cavity with co-resonating acoustic and optical modes is desirable. For the applications demanding large bandwidth (e.g. optical isolator and mode convertor), a spiral shape multi-pass OM waveguide is ideal [169]. However, achieving higher AO modulation without sacrificing bandwidth remains an open question in both cases.

*Heterogeneous integration.*

Wafer-scale heterogeneous integration of non-piezoelectric and piezoelectric materials can be advantageous for IAO devices to leverage the strengths of different materials. For instance, LN-on-silicon nitride (SiN) [170] combines LN’s excellent acoustic properties with SiN’s low optical loss. By integrating different materials, preferable acoustic and optical characteristics can be preserved. Exploring more material combinations can open new design spaces for high-efficiency AOMs. Pairing materials with high acoustic speed and high refractive index contrast enables high OM coupling and low acoustic loss without a suspending structure. Additionally, separating piezoelectric and non-piezoelectric regions can reduce unwanted stray electric fields and crosstalk.

### Concluding remarks

The technology evolution from conventional bulk modulators to planar and guided-wave IAO devices represents a crucial improvement in the AO technology. Despite significant progress, challenges persist in maximising AO modulation efficiency, scalability, and materials innovation. Addressing these challenges requires new device architectures, heterogeneous material platforms, and innovative designs. Tools like direct acoustic imaging offer insights into optimising device geometries, while miniaturisation and heterogeneous integration strategies hold promise in enhancing performance and compatibility. New opportunities for IAO research in optical signal modulation, communication, and quantum applications also require interdisciplinary effort to realize the full potential of IAO devices.

## Acknowledgements

I C and M L are supported by NSF Award Nos. EECS-2134345 and ITE-2134345. K L is supported by the NSF Electrical, Communications, and Cyber Systems Award ECCS-2221822.

## 12. 2D materials and van der Waals systems

### Geoff R Nash^1^ and Emeline D S Nysten^2^

^1^ Natural Sciences, The University of Exeter, Exeter, United Kingdom

^2^ Physikalisches Institut, Universität Münster, Wilhelm-Klemm-Str. 10, 48149 Münster, Germany

E-mail: emeline.nysten@uni-muenster.de and G.R.Nash@exeter.ac.uk

### Status

Since the publication of the 2019 surface acoustic waves roadmap [9], research into the interplay of 2D materials and SAWs has continued at a steady pace (for a more comprehensive review see [171]). These new materials, the first of which to be isolated was graphene in 2004, naturally lend themselves for integration with SAWs. Their relatively large surface area, and sensitivity to external stimuli, makes them an obvious route to develop higher sensitivity SAW sensors. In addition, the inherent thinness of these materials means that not only will they interact mechanically with SAWs, but also that the electric fields generated by a SAW propagating on a piezoelectric substrate can interact with any charge carriers present. The interactions between SAWs and 2D materials is thus an exciting testbed to study the properties of new materials but could also ultimately form the basis of new devices.

**Figure 19.**
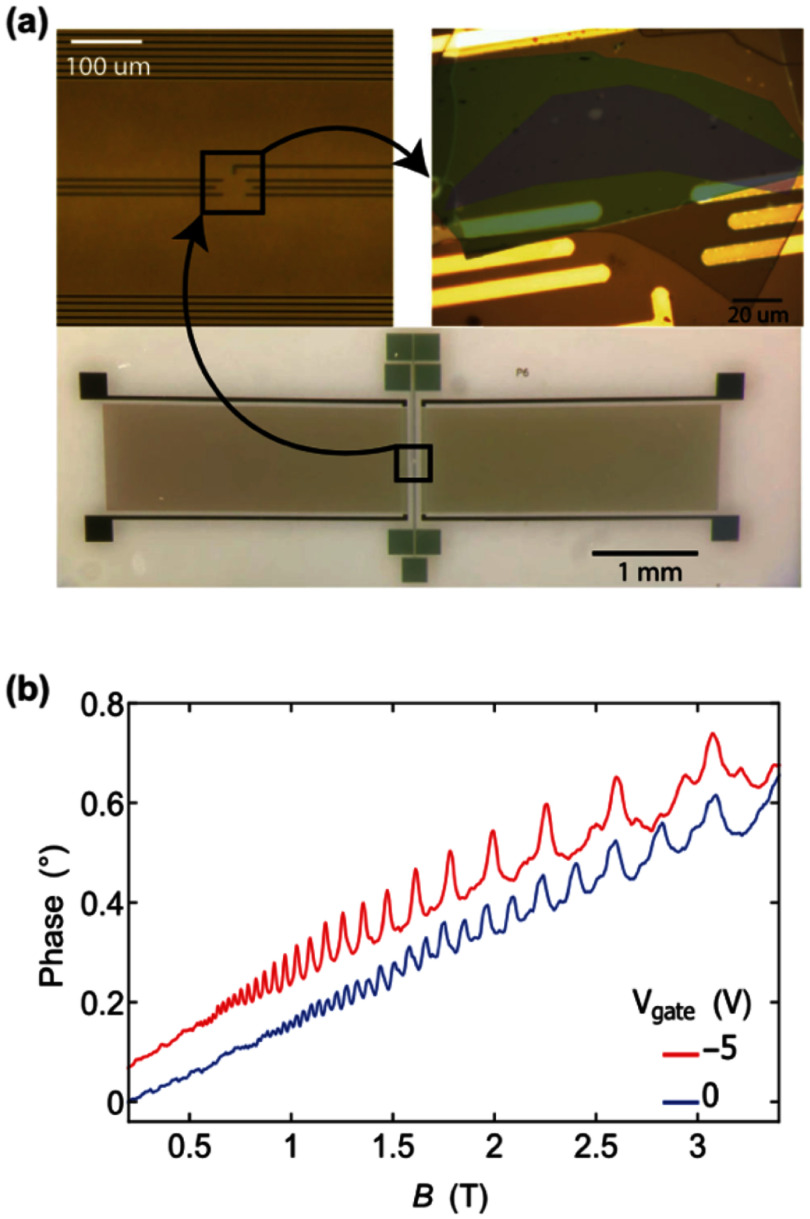
Observation of quantum transport in graphene using a surface acoustic wave resonator. (a) Optical microscope image of the device showing a view of the full SAW cavity in the lower image, an enlarged view of the of the centre of the cavity with the detail of the prepatterned gold electrodes without the graphene sample in the upper left image and a false-colour image showing the graphene device in detail in the upper right image. The monolayer graphene is coloured in light purple, and the graphite top gate is coloured in light green. (b) The change in the cavity phase at the fixed resonant frequency of the SAW resonator, as a function of magnetic field, at two different gate voltages, and at 1.7 K. The quantum oscillations are clearly visible above 0.6 T. The data have been offset for clarity. Reprinted (figure) with permission from [23], Copyright (2023) by the American Physical Society.

Although many new 2D materials are now available, interactions between graphene and SAWs are still the focus of much research, and graphene’s potential as an extraordinarily responsive sensing material has continued to be exploited for the development of a wide range of SAW sensors [172] and microfluidic devices. Acoustic charge transport, where the piezoelectric fields associated with a propagating SAW can be used to trap and transport charge is also an area of much interest. Uniquely to graphene in acousto-electric devices, the AE current in the same device can be reversed, and switched off, using an applied gate voltage to dope the graphene and change the majority charge carrier. In addition to this classical transport regime, recent research, which has used 2D hexagonal boron nitride (h-BN) as a gate material [23, 173], have demonstrated AE current in the quantum regime, with the observation of Shubnikov de Haas oscillations in large magnetic fields demonstrating quantised 2D subbands, and mirroring early work on the use of SAWs to probe the properties of 2D electron gases in AlGaAs/GaAs heterostructures (cf figure 19). Additionally, SAWs were used to artificially induce gauge fields in the absence of external magnetic fields [174].

**Figure 20.**
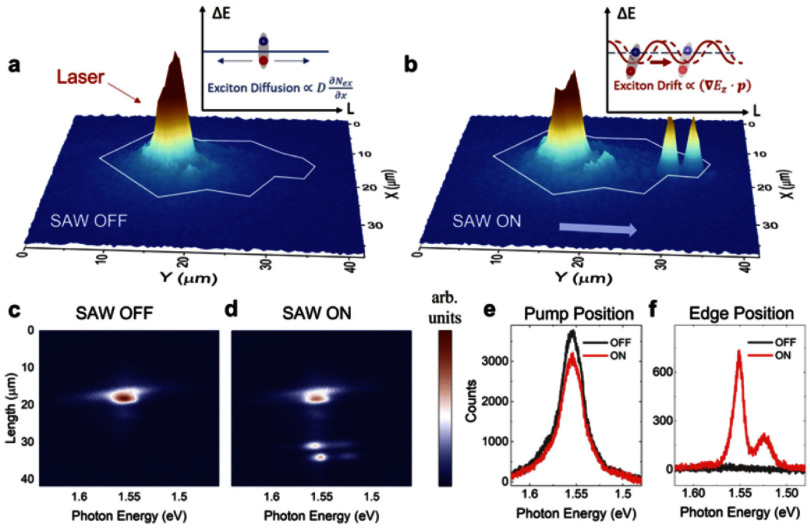
SAW-driven transport of interlayer excitons (IXs) at 100 K. Real-space photoluminescence (PL) mapping (a), when SAW is off, and (b) when SAW is on with 6 mW power. Two bright emission spots appear at the edge of the flake and the focal point of the acoustic wave due to the SAW-driven transport of IXs. Insets: Illustration of the free diffusion and SAW-driven drift of IXs. (c), (d) The spectral PL image in the same experimental conditions as for (a) and (b), respectively. The non-local exciton emission at the flake edge is clearly attributed to the IXs in WSe_2_, which have an emission peak at 1.56 eV. (e) The emission spectrum at the pump position slightly decreases when the SAW is turned on. (f) The emission spectrum at the flake edge position drastically increases when SAW is turned on. Reproduced from [176]. CC BY 4.0.

The other most studied class of 2D material has been that of TMDCs, including materials such as molybdenum- and tin-disulphide. In contrast to graphene, these materials have intrinsic bandgaps, leading themselves to the study of excitonic effects, but also the development of novel optoelectronic devices, with higher performance and new device functionality. For example, Datta *et al* [175], Scolfaro *et al* [82] and Peng *et al* [176] have all demonstrated successful transport of excitons in TMDC monolayers and interlayer excitons in bilayers over long ranges (limited by flake size, see figure 20), whereas Alijani *et al* [177] and Zhao *et al* [178] demonstrated optoelectronic device enhancement using 2D materials combined with SAWs and Nysten *et al* [179] showed that SAW-based scanning acousto-electric spectroscopy provides a highly sensitive and local contact-free probe to uncover distinct local features in TMDCs monolayers such as local changes in the dielectric and strain environment, i.e. tears, folds etc, induced by the exfoliation and transfer process.

### Current and future challenges

A continuing challenge is the ability to prepare large area, high quality, samples of 2D materials. Chemical vapour deposition techniques for the growth of materials such as graphene and h-BN have continued to advance, with growth on metal substrates being the predominant approach. However, thus still necessitates transfer of the 2D material of interest onto the preferred substrate, which for SAW applications is likely to be quartz or LN. The transfer process itself can often introduce imperfections, such as wrinkles, which not only limit the carrier mobility, but also make the process somewhat irreproducible. There have been very few reports of direct epitaxial growth of 2D materials onto SAW substrates [180, 181], and a key technological challenge is how to reproducibly obtain large area, high quality 2D materials on SAW substrates.

As predicted in the 2019 surface acoustic waves roadmap [9], the use of hBN as an encapsulating dielectric has proven to be a good solution to improve the quality of the transferred 2D materials on rough substrates. However, while the piezoelectric field of the SAW can be correctly transmitted through the dielectric, the mechanical adhesion of the hBN on the SAW substrate is weak and can lead to the loss of the SAW strain inside the 2D material under study. This fact was highlighted by the study of the emission of defect centres in hBN under acoustic strain [182].

For less mature 2D materials, such as (TMDCs), most of the reported work integrating with SAWs have been based on the use of mechanically exfoliated single crystal flakes, which tend to be high quality (low numbers of defects) and therefore, for example, have high electron mobility. Although applications will require scalable device architectures that are cost effective, in some senses SAWs provide an excellent tool for studying the properties of these flakes, which only a few tens of micrometres in size, with less rigorous contacting and positioning requirements compared to all electrical approaches. Recently, SAW driven magnon–phonon coupling [183] was demonstrated using a flake of chromium trichloride, a material consisting of layers, of alternating magnetisation, weakly bound together by Van der Waals attraction. This shows that advances in materials technology will underpin exciting new research avenues that integration of SAWs and 2D materials offer.

### Advances in science and technology to meet challenges

While there are very few reports on the CVD growth of 2D materials directly on SAW substrates such as LiNbO_3_, other techniques could be applied to resolve such an issue. For example, Xu *et al* have developed a technique for the direct fabrication of few-layer graphene on LiNbO_3_ by implanting Carbon ions in a Copper covering layer, which is later removed [181]. Alternatively, non-piezoelectric substrates adapted for the CVD growth of 2D materials, i.e. silicon, can still sustain SAW modes, which can be excited by the deposition of thin film piezoelectric materials such as Zinc Oxide, LiNbO_3_, etc. A lot of progress has been made for the fabrication of such hybrid substrates. Additionally, the requirement for higher frequencies in the several GHz to tens of GHz regime for the investigation of new emergent 2D Materials, such as the excitation of SWs in ferromagnetic 2D materials, i.e. CrI_3_, requires this type of thin film structures, i.e. LiNbO_3_ on Sapphire or on Silicon and have already been investigated for the excitation of high frequency SAW devices [184, 185].

In parallel to the possible direct growth of 2D materials on SAW substrates, advances in synthesis technique have also opened a large range of possible new 2D materials and possible properties. It is possible that, as the range of materials available to research increases, the possibility of easier incorporation in SAW devices becomes more probable. The recent use of artificial intelligence and machine learning methods for materials discovery has enabled an even quicker expansion of this research field [186].

Advances in phononic metamaterials [187] are also likely to have an increasing impact on this research. The ability to control and manipulate the propagation of SAWs, as with photonics, is likely to lead to enable new fundamental studies and device concepts for which the integration of 2D materials is extremely well suited. For example, the ability to confine the acoustic energy in a very small volume [188], or acoustic cavity, would increase the interaction of a SAW with a flake of 2D material, further increasing the ability of the SAW to probe the properties of the material, but also allowing the development of new sensors. The unique properties of 2D materials, including their mechanical flexibility, may also allow new approaches for realisation of dynamically tuneable phononic metamaterial arrays. Additionally, van der Waals materials are very sensitive to strain [189] and these new phononic tools would present an excellent platform to probe these effects at MHz and GHz frequencies.

### Concluding remarks

Since the last roadmap article, the field of acoustic waves and 2D materials has been flourishing. The research on graphene has expanded and includes now the study of charge carrier dynamics in the quantum regime and the fabrication of highly sensitive sensor and microfluidic devices. The study of TMDCs integration in SAW devices has also increased and exciton transport and improved optoelectronic properties have been proven. This increase in the successful fabrication of efficient and effective electronic and optoelectronic devices with an increasing range of 2D materials demonstrates the technological potential of 2D materials-SAW hybrid devices but also the great potential of SAWs for the contactless study of many fascinating fundamental properties such as charge carrier dynamics, exciton transport, SWs excitation, etc in new 2D materials. The direct growth of high quality 2D materials on SAW substrate remains the main challenge to be addressed, but the fast-paced improvement in growth and synthesis techniques for 2D materials as wells as SAW thin film substrates has the potential to overcome this problem, especially with the addition of machine learning and AI for materials discovery.

## Acknowledgements

G R N acknowledges financial support from the Engineering and Physical Sciences Research Council (EPSRC) of the United Kingdom (A-Meta: A UK-US Collaboration for Active Metamaterials Research, Grant No. EP/W003341/1).

E D S Nysten acknowledges funding from the Deutsche Forschungsgemeinschaft (DFG) through the priority program SPP 2244 (Grant No. 535379969) and support from the Alexander von Humboldt Foundation in the framework of the Research Group Linkage Program funded by the German Federal Ministry of Education and Research.

## 13. Coupling between surface acoustic waves and organic semiconductors

### Paromita Bhattacharjee^1^, Himakshi Mishra^2^, Parameswar K Iyer^3^ and Harshal B Nemade^4^

^1^ Physikalisches Institut, Universität Münster, 48149 Münster, Germany

^2^ Department of Electronics and Communication Engineering, Manipal Institute of Technology, MAHE, 560064 Bangalore, India

^3^ Department of Chemistry, Indian Institute of Technology Guwahati, 781039 Amingaon, India

^4^ Department of Electronics and Electrical Engineering, Indian Institute of Technology Guwahati, 781039 Amingaon, India

E-mail: paromita.b@uni-muenster.de, himakshi.mishra@manipal.edu, pki@iitg.ac.in and harshal@iitg.ac.in

### Status

**Figure 21.**
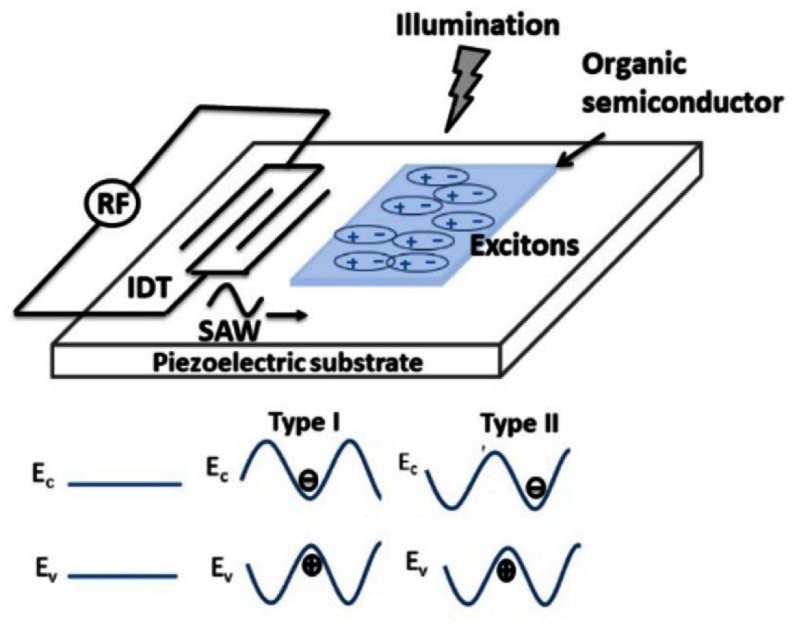
Schematic of SAW-OS device structure describing the energy band modulations. Reproduced from [16]. © IOP Publishing Ltd. All rights reserved.

The interaction between SAWs and inorganic semiconductors is a well-established research area. The strain and piezoelectric fields of SAW alter the electrical characteristics of a semiconductor via type-I and type-II band-edge modulations, respectively, as shown in figure 21 [190]. These modulations instigate spatial separation of charge carriers in an exciton, leading to acousto-optoelectric (AOE) effect. Additionally, AE effect—where the momentum of SAW exerts a dragging force on charge carriers—offers promising applications in charge transport, demonstrating increase in conductivity and current in various inorganic semiconductor-based devices including III–V and 2D materials [180, 191, 192]. Nonlinear interactions between counter-propagating SAWs give their convolution which is widely used in communication systems, and inorganic semiconductors incorporated in air-gap and monolithic convolvers [193, 194] enhance the otherwise weak non-linearity of typical piezoelectric substrates.

Conversely, use of organic semiconductors (OSs), known for low-cost solution-processing fabrication, mechanical flexibility and biocompatibility, is a comparatively new concept in the exploration of acoustic interactions [12]. Although OS offer prospective sustainable and flexible device applications, like wearable sensors, RFID tags, etc, they are not crystalline and suffer from trap states. Overcoming potential barrier between traps and grain-boundaries limits efficient charge transport and causes instability. Acoustic influence on poly(3-hexylthiophene-2,5-diyl) (P3HT) shows that momentum from SAW can reduce effective energy difference between these trap states [195]. AE effect remains directional showing large increase in current when charges are biased in the direction of SAW. Similar effect predominates in P3HT transistors tested with variable channel widths indicating evenly distributed charge transport pathways across the thin film [196]. This key experiment showed improved stability by compensating long-term charge trapping effects. Additionally with photoexcitation, OS such as P3HT and poly[2-methoxy-5-(2-ethylhexyloxy)-1,4-phenylenvinylen] (MEH-PPV) show quenching in photoluminescence intensity wherein SAW fields help overcome the exciton binding energy, *C_BE_* [16]. The maximum charge transport capacity, *N*_max_ shows dependency on SAW power and wavelength, though independency of light intensity. However, at high intensities reaching *N*_max_ limits the exciton ionisation which drops the overall efficiency. In addition to these fascinating inter-dependencies, use of orthogonal SAWs shows possibility of information routing via diagonal movement of charges in MEH-PPV [197]. As a step forward towards all-optical devices, device engineering approach such as inclusion of metal–insulator-OS layers in SAW path enables electrical control of exciton flux leading to excitonic transistor characteristics [198]. Furthermore, low intrinsic charges in P3HT aided in recording the highest efficiency achieved in a SAW convolver [199].

### Current and future challenges

*Fabrication complexities:* Larger section of OS degrade over time upon exposure to air, moisture and light, often leading to device instability, which is one of the primary challenges in the study of SAW interactions with OS. Encapsulation methods that do not interfere with SAW propagation or the intrinsic properties of OS are crucial, and developing advanced strategies such as multilayered barrier materials transparent to SAWs, is essential for device longevity and reliability. Another significant challenge lies in the fabrication of multilayered devices, hindered by the chemical incompatibility between conventional photoresists and OS. During the fabrication process, photoresists can react with the OS, altering or damaging the underlying layers, making it difficult to construct stable, multilayered structures necessary for advanced device applications. Alternative patterning techniques can be developed to avoid the use of traditional photoresists. Methods such as soft lithography [200] or nanoimprint lithography [201] can be employed with innovative approaches to layered fabrication, such as additive manufacturing techniques, to preserve the integrity of OS layers. Hybrid material systems that combine the beneficial attributes of OS with the stability of inorganic materials can be imperative for creating more robust devices. Additionally, interface engineering, such as surface treatments and the use of buffer layers, is critical to ensure good adhesion and minimize degradation at the interfaces between different materials. A holistic approach to device design, integrating comprehensive consideration of the required device performance and the establishment of standardized testing protocols for long-term stability and performance evaluation, is essential for developing devices that can reliably operate under variable environments.

*Experimental conditions:* High strain levels in materials commonly used for SAW devices, such as LN and tantalate, pose significant challenges in the precise characterisation of organic device performance. Managing strain becomes crucial during operational analysis, as it may lead to stress induced morphological changes in some softer materials. Understanding and mitigating the effects of strain on these materials is essential for ensuring the reliability and practical applicability of OS integrated SAW devices. Future challenges include engineering devices with flexible substrates as the piezoelectric layer, to enhance mechanical durability for SAW applications in flexible electronics. Additionally, technique for improving charge transport within OS like achieving chain alignment during the thin film formation via application of direct current electric field as promisingly observed in MEH-PPV [202] can also be opted.

### Advances in science and technology to meet challenges

**Figure 22.**
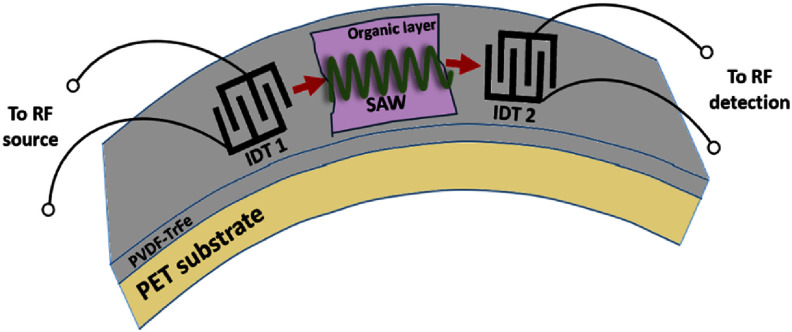
Schematic of a prospective polymeric flexible SAW-OS device.

*Flexible SAW:* The main yield of coupling OS with SAW lies in fabricating flexible devices like shown in figure 22 which can harness the potential of the underlying interaction along with substantial reduction of the total production cost. Till date, experiment studying the propagation of SAW in polymeric and flexible piezoelectric substrates remains confined to materials such as poly(vinylidenefluoride-trifluoroethylene) (PVDF-TrFe) [203], ZnO [204] and single crystalline LiNbO_3_ thin film [205]. These devices show inconsequential degradation in SAW characteristics under bending and deformation conditions suggesting prospects of utilising flexible substrates with OS. Although, the restricted use of the low-cost polymeric piezoelectric materials has been mainly because of their low piezoelectric coupling coefficient leading to low operating frequency. To counter-act, with polymeric piezoelectric materials, engineering of the traditional SAW device structure can enhance the piezoelectric response, like using parallel double layers of IDTs [203] enabling efficient poling of the thin film by aligning dipoles with applied electric field, wherein the PVDF-TrFe based polymer SAW devices have also shown air stability with no perceptible loss of poling. Proper solvent selection [206] and electrospinning [207] are other simple and effective methods which have shown to improve piezoelectricity in PVDF and its copolymer PVDF-TrFe, showing wide usage in self-powered sensors to smart skin and electronic textiles. The frequency range till which these materials can sustain bending and deformations is another interesting aspect yet to be addressed.

*Hybrid material system:* Since the study of the interaction between an OS and SAW is very new [12] and has been established with only few polymers such as P3HT [195] and MEH-PPV [16], AE and AOE effects need to be studied in more variety of OS and materials like perovskites to bring together a larger database exploring the underlying phenomenon caused by the coupling with SAW. For example, band aligned hybrid and heterostructure layers of these materials can lower the effective *C*_BE_, instead of higher *C*_BE_ often seen in the individual materials, and offer efficient charge transport via AOE effect. Heterostructure layers with tuned capacitive properties attributing to stronger depletion can help further enhance efficiency of SAW convolvers.

### Concluding remarks

The coupling of SAW with OS offers an exciting field for research as it is yet in the early stage and delivers stimulating underlying phenomenon leading to modulation of charge and exciton transport. The biggest advantage of using OS lies within the ease of low-cost solution processing fabrication and its applicability in flexible devices. Utilising these with existing SAW technology can help devise highly efficient signal processing units, charge transfer devices, optoelectronic components, wearable and disposable sensors. Resolving of some interesting challenges with process and device engineering can further ensure wider reliability and practical applicability of these systems.

## Acknowledgements

The authors acknowledge the Women in Research (WiRe) postdoctoral fellowship received from Universität Münster, Germany for supporting this work. Part of this work has received financial support from the Ministry of Electronics and Information Technology (MeiTY) Grant 5(1)/2021-NANO, 5(1)/2022-NANO, the Department of Science and Technology (DST) Grant DST/CRG/2019/002614, the Indian Council of Medical Research (ICMR) Grant 5/3/8/20/2019-ITR, Max-Planck-Gesellschaft IGSTC/MPG/PG(PKI)/2011A/48, and the Ministry of Human Resources and Development (MHRD), India. The authors also acknowledge the Centre for Nanotechnology, Central Instruments Facility and DST- sponsored Organic Electronics Laboratory, at the Indian Institute of Technology (IIT) Guwahati for help and support.

## 14. Spatial confinement of elastic energy in phononic crystals and metamaterials: advances, challenges, and future directions

### Abdelkrim Khelif^1,2^, Sarah Benchabane^1^, Gao Feng^3^ and Yabin Jin^4^

^1^ Institut Femto-st, UMR6174 CNRS, Université de Franche-Comté, Besançon, France

^2^ College of Science and Engineering, Hamad Bin Khalifa University, Doha, Qatar

^3^ ZJU-HangZhou Global Scientific and Technological Innovation Center, Zhejiang University, Hangzhou, People’s Republic of China

^4^ Institute of Computational Mechanics × AI & College of Intelligent Robotics and Advanced Manufacturing, Fudan University, Shanghai, 200433, People’s Republic of China

E-mail: abkhelif@hbku.edu.qa, gao.feng@zju.edu.cn and yabinjin@fudan.edu.cn

### Status

**Figure 23.**
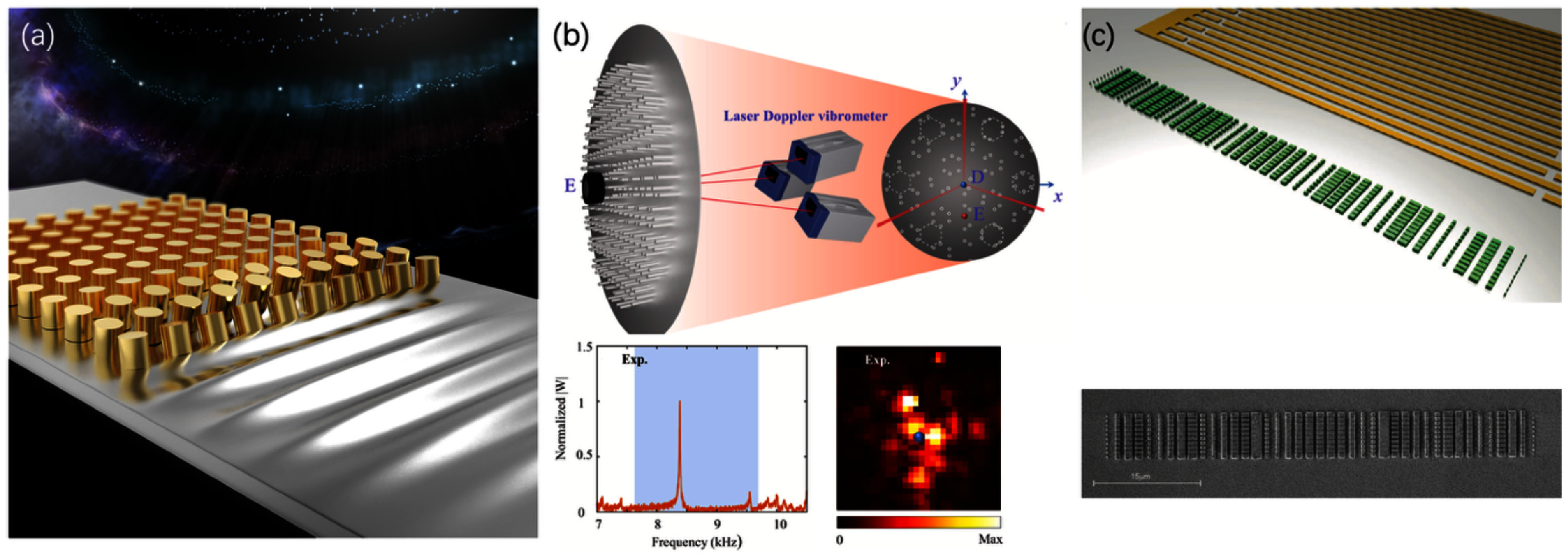
(a) Illustration of phononic crystals/acoustic metamaterials consisting of periodic pillars deposited on top of a plate which interact with Lamb waves. (b) Geometric structure of topological vortex mode for antisymmetric Lamb wave with Jackiw–Rossi binding mechanism. The lower panel shows experimentally measured response peak and out-of-plane displacement field of the topological vortex mode. Reproduced with permission from [208]. CC BY-NC 4.0. (c) Illustration of SAW metasurfaces marked as green (upper panel) and fabricated sample (lower panel). Reproduced from [209]. CC BY 4.0.

Phononic crystals and acoustic metamaterials constitute an emergent field with immense potential for spatial confinement and waveguiding of acoustic or elastic energy. These materials, characterized by periodic structures or locally resonant elements, offer novel avenues for manipulating acoustic or elastic waves as seen the illustration in figure 23(a), akin to the control exerted by photonic crystals, metamaterials, and metasurfaces over light. This manipulation spans various wave types, including SAWs, Lamb waves, and beam waves offering potential breakthroughs in fields ranging from telecom technology to healthcare.

A key advantage of phononic crystals lies in their capacity to regulate wave propagation by introducing specific patterns or altering the composition of materials [210]. This capability holds promise for diverse applications, such as sensing [211], energy harvesting [212], telecommunication filters [213], and quantum technology [214]. Especially for energy harvesting, phononic crystals and acoustic metamaterials are able to improve the power density, bandwidth and robustness of acoustic/elastic energy for energy harvesting, based on various mechanisms such as compact cavity/defect modes, topological states, localized resonant vibrations, etc.

Researchers have pursued various designs and configurations of phononic crystals and metamaterials, aiming for efficient waveguiding, particularly at high frequencies. The focus of optimisation has primarily centred on the geometric characteristics of the crystal lattice including the size and shape of constituent unit cells, alongside material properties to attain the widest and all-direction band gaps in the ideal crystal. Different defects inside the perfect crystal were considered in order to control the wave dispersion including the phase and the group velocities of the guided modes.

Recent explorations have extended to alternative guiding mechanisms like non-reciprocal, topological, and time-dependent metamaterials. Non-reciprocal metamaterials [215], with their asymmetric wave transmission properties, are crucial for applications such as isolators, circulators, and directional waveguides. Recent research endeavours have focused on developing non-reciprocal acoustic and elastic metamaterials employing materials like magnetoelastic, piezoelectric, or active substances with broken time-reversal symmetry. Topological metamaterials, drawing from topological physics, engender robust and protected wave transport phenomena, offering unique properties like immunity to defects and disorder [216]. For example, topological vortex mode is designed in pillared phononic crystal plate for antisymmetric Lamb wave with Jackiw–Rossi binding mechanism [208], as shown in figures 23(b) and (c). Efforts in this domain have aimed at designing structures with non-trivial band topologies, exploiting geometric and topological features to achieve desired wave behaviours.

### Current and future challenges

**Figure 24.**
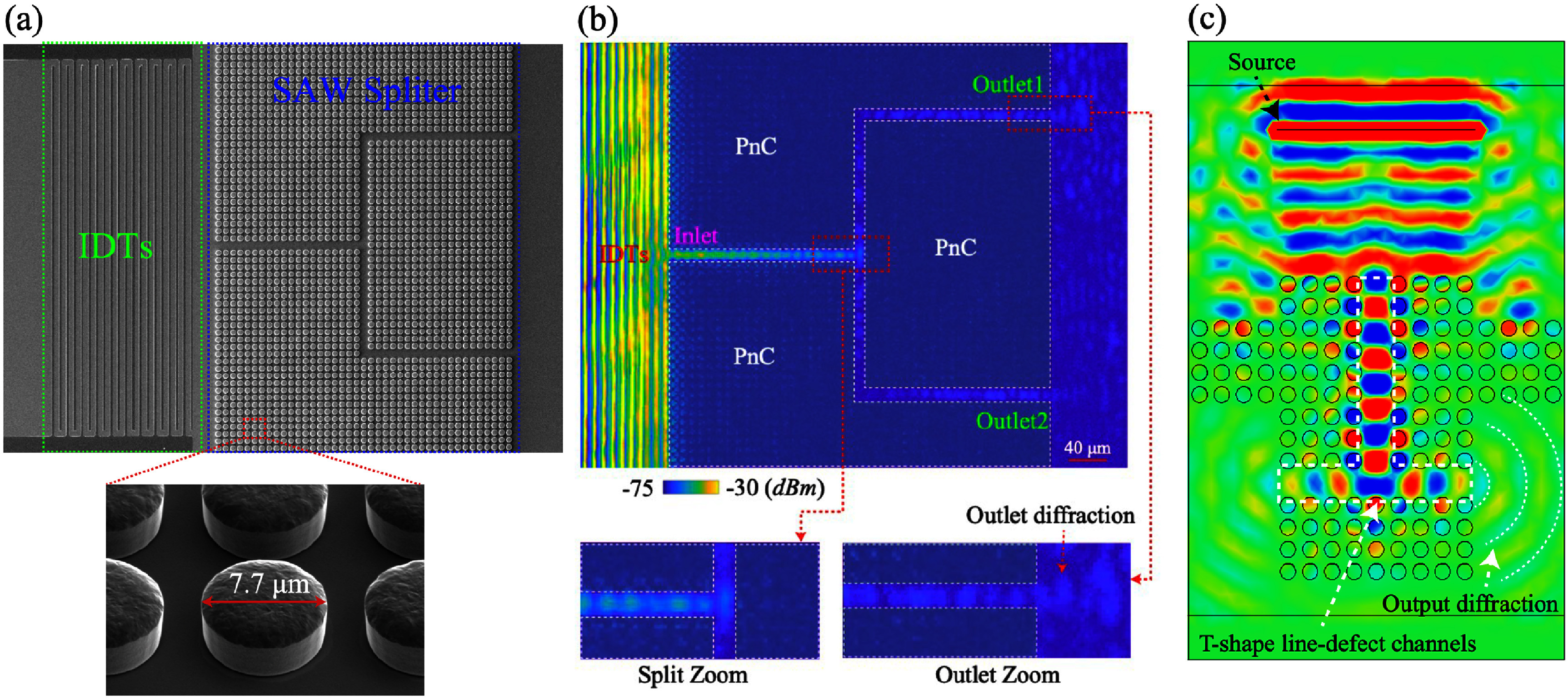
(a) A line-defect phononic waveguide of 20 *μ*m wavelength fabricated by the electroplating process. (b) Surface displacement showing the SAW splitting of the waveguide. (c) Simulation of the line-defect phononic waveguide by finite element method.

Despite significant advancements, challenges persist, particularly in implementing and scaling phononic crystals and metamaterial structures for practical use. For instance, coupling phononic waveguides with photonic devices, at submicron scale, enables novel functionalities in integrated photonics, facilitating efficient interactions between light and sound in OM systems. Refinement of fabrication techniques is crucial to achieve the necessary precision, especially for high-frequency applications. Additionally, integrating phononic waveguides with other technologies, such as photonic, plasmonic, and electronic devices, as well as hybrid systems like sensors or communication devices, requires further development. In addition to traditional fabrication methods such as CNC machining, laser cutting, advanced fabrication techniques, especially additive manufacturing and micro/nanofabrication, are accelerating the development of these materials with customizable designs. However, there are three main future challenges that need to be addressed:

1. The challenge of size minimisation. The fundamental properties of the phononic crystals and acoustic metamaterials originate from their dispersion curves that showing the matter-wave interaction. In another word, it normally requires sufficient number of unit cells resulting in bulky size of the entire structure. It urgently needs to minimize the geometric size of the phononic crystals and acoustic metamaterials while conserving their versatile wave functions in order to improve the application flexibility. Recent advances reported by Feng *et al* such as the fabrication of a line-defect phononic waveguide with a 20 *μ*m wavelength using electroplating, demonstrate progress in this area (figure 24(a)). The waveguide demonstrates the capability of guiding, bending and splitting of SAW (figure 24(b)).
2. The challenge lies in advancing structural fabrication technology to meet the growing demands for the wave functions of phononic crystals and acoustic metamaterials. With increasingly complex geometry configurations and the use of multiple materials, a delicate balance between available fabrication precision and structural requirements must be struck. There is a pressing need for more sophisticated fabrication techniques capable of realising desired structural configurations at various scales with multiple composition of materials.
3. The challenge of intelligent design and functional application. There are strong nonlinear mapping relationships between the structural parameters and designed function properties. Currently, a lot of efforts are cost in trial-and-error test. Finite element method is a common tool for modelling phononic waveguides with relatively good precision (figure 24(c)), but the computation complexity is high. Intelligent algorithms, such as AI-driven, data-driven or environment interaction, are urgently needed to predict functions in forward design and efficiently determine structural parameters in inverse design with the requisite precision. Furthermore, identifying potential applications for phononic crystals and acoustic metamaterials across different industries with real-world working conditions is imperative for their widespread adoption and practical implementation.

### Advances in science and technology to meet challenges

Elastic metasurfaces [217], rapidly developed in recent years, significantly improve the minimisation of structural size while possessing rich and power wave functions. It takes the advantage of the nonlinear wave response of subwavelength-thickness structures and manipulates the wave functions efficiently. The downsizing of phononic crystals and acoustic metamaterials or even elastic metasurfaces is also commonly achieved through microfabrication techniques, notably photolithography. To be specifically, SAW metasurfaces show promising potential in high frequency applications such as NEMS, sensing, communications, quantum processing. This method facilitates the precise patterning of photoresist with resolutions reaching a few hundred nanometres, subsequently translating into the desired structures via either etching or electroplating. While electroplating is favoured for metallic structures, etching predominates for dielectric materials, offering the advantage of uniform structure heights across the device. To further shrink the structures, e-beam lithography is often employed, yet its costliness and time-intensive nature pose constraints, particularly for large array production. To mitigate this challenge, nano-imprinting has emerged as a viable alternative, extensively utilized in nano-scale photonic crystal manufacturing. Nano-imprinting entails the initial fabrication of an imprinting mold from a durable material such as silicon, followed by transferring the pattern from the mold to a desired substrate through the imprinting process.

Advancements in fabrication techniques and substrate preparation methods have facilitated the creation of phononic crystals and acoustic metamaterials with intricate spatial structures and material compositions. Additive manufacturing, particularly 3D printing, has played a significant role in this advancement. 3D printing allows for the fabrication of phononic crystals with complex 3D unit cells, departing from traditional structures like holes or cylinders. However, conventional 3D printing is limited to structures with critical dimensions in the tens of microns range. Recent progress in 3D printing, specifically through 2-photon polymerisation, enables the fabrication of complex 3D unit cells at the nanoscale, albeit restricted to polymer materials. Integrating 2-photon polymerisation with electroplating could potentially extend its applicability to metallic unit cells. In terms of composite material stacks, thin-film piezoelectric substrates prepared via smart-cut techniques have been explored, including LN and LT films on insulator substrates. This approach opens avenues for fabricating phononic crystals with intricate material compositions. The development of machine learning as well as other optimisation methods such as topology optimisation and genetic algorithm can help to accelerate the efficiency in the forward prediction and the inverse design for phononic crystals and acoustic metamaterials [218]. Considering the fabrication constrains in the current technologies in the design process, the development of phononic crystals and acoustic metamaterials will adapt to practical needs and industrial applications.

### Concluding remarks

In conclusion, phononic crystals and acoustic metamaterials represent a growing field with vast potential applications in various industries, from technology to healthcare. These materials offer innovative avenues for manipulating elastic and vibrations with highly spatial confinement. Despite notable progress, significant challenges persist, particularly in implementing these materials for practical applications, especially at high frequency. This includes the imperative to minimize structural size while preserving versatile wave functions by controlling the elastic absorption phenomena that often occur at high frequency in deposited materials. The need for advancements in structural fabrication technology to meet evolving demands, and the necessity for intelligent design and functional application to overcome nonlinear mapping relationships between structural parameters and desired properties. Addressing these challenges requires interdisciplinary collaboration and innovative approaches. Advanced fabrication techniques, such as 3D printing and nano-imprinting, coupled with machine learning algorithms, hold promise for overcoming these obstacles.

## Acknowledgements

This work is supported by the Region of Franche-Comté (ref. 2012C-08901), the French RE-NATECH network, and its FEMTO-ST technological facility, as well as by various Chinese funding sources, including the National Natural Science Foundation of China (12272267), the Young Elite Scientists Sponsorship Program by CAST (2021QNRC001), the Shanghai Science and Technology Committee (Grant No. 22JC1404100), the National Key R&D Program of China (No. 2022YFB3604500), and several Zhejiang Province Key R&D Programs (Nos. 2021C05004, 2023C03076, 2023C01192, 2023R01011, and 2024SSYS0042).

## 15. Piezoelectric thin films for novel microacoustic devices

### Ausrine Bartasyte^1,2,3^ and Samuel Margueron^1^

^1^ Université Marie et Louis Pasteur, ENSMM, FEMTO-ST Institute 26 rue de l’Epitaphe, 25030 Besançon, France

^2^ Université Paris-Saclay, CNRS, Centre de Nanosciences et de Nanotechnologies, 10 Bd Thomas Gobert, 91120 Palaiseau, France

^3^ Institut Universitaire de France 103 boulevard Saint Michel, 75005 Paris, France

E-mail: ausrine.bartasyte@femto-st.fr and samuel.margueron@femto-st.fr

### Status

**Figure 25.**
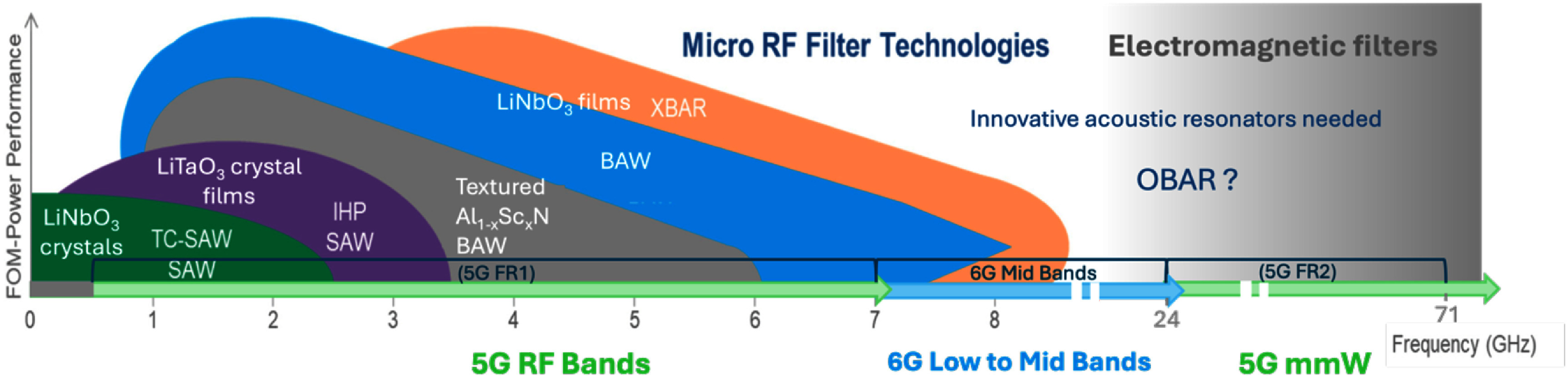
5 G and 6 G band allocation in the frequency range from sub-GHz to 71 GHz, along with the RF and mm-wave (mmW) filter technologies considered. Reproduced from [220]. CC BY 4.0.

The 5th and 6th generation communication networks (5 G and 6 G) promises to revolutionize modern society. These advancements will unlock a new era of possibilities in various fields, including the internet of things (IoT), a rapid transmission of massive amounts of information, an extreme user and device density for smart cities and connected ecosystems, the intelligent transportation (autonomous vehicles), smart manufacturing, etc [219]. To respond to these needs, the electromagnetic spectrum needs to be expanded and number of RF bands, dedicated to data transmission, have to be increased in line with the need of increased bandwidths and high frequencies of the acoustic filters, based on piezoelectric materials, which is a significant technological challenge [220] (figure 25). RF filter technology, based on SAW devices using piezoelectric materials such as LiNbO_3_ and LiTaO_3_ single crystal wafers are limited to 2.5 GHz due to limitations in terms of quality factor and heat dissipation, while incredibly high performance SAW filters, based on LiTaO_3_ single-crystal 1–2 *μ*m thick films, fabricated by polishing, mounted on a multilayer structure to confine the energy to the surface have made it possible to extend the frequencies of SAW components up to 3.5 GHz [220, 221]. BAW devices using sputtered piezoelectric AlN and Al_1−*x*_Sc*_x_*N thin films on electrode/(Bragg mirror or membrane)/Si structure enables operational frequencies up to 4 GHz and 8 GHz, respectively [222], responding to the RF communication needs of frequency bands up to 6 GHz for 5G technology. The number of filters in the smart phones already approaches 100 requiring significant space therefore tunable and reconfigurable filters are highly demanded. The future 6G communications will require RF spectrum in the range of 10–26 GHz [220, 221]. Small size rf filters, operating at 30–50 GHz are also of special interest as an alternative for bulky electromagnetic filters in the extreme density circuits [221].

Furthermore, piezoelectric effect and acoustic waves are also explored for numerous functionalities in the integrated photonic or phononic circuits: for AO modulators, piezo optomechanic components, and low loss acoustic wave routing [223, 224]. AO operation offers low-power consumption and low-noise operation contrary to standard electro-optic approach or thermo-optic components. The strain field generated by acoustic waves offers also a complementary method to optical and MW techniques for manipulating the spins, which holds significant promise for applications in quantum communication, sensing or tunable/nonreciprocal RF devices [225, 226]. Furthermore, highly coupled piezoelectric thin films can be considered also for the nanoscale positioning/alignment, and for generation of controlled perturbations and stimuli.

### Current and future challenges

Although acoustic devices operating up to 50 GHz were reported, it is still highly challenging to attain the required performance of acoustic devices for filter design in terms of quality factor (*Q* > 1000), EM coupling (*K*^2^ = 10%–20%), impedance matching (50 Ω), and high-power handling capability at these frequencies [221]. The frequency of BAWRs can be easily increased by reducing the thickness of the piezoelectric thin film. However, a reduction in the effective *K*^2^ of BAWRs due to the presence of electrodes, temperature compensation layers, and mirror becomes significant in the case of very thin piezoelectric layers (typically <300 nm thick). Reducing the thickness of electrodes increases (for example, 100 nm thick Al layer sheet resistivity is around 1–2 Ω sq^−1^, while 10 nm thick Al film presents resistivity >150 Ω sq^−1^) their resistance which consequently deteriorates the quality factor. According to Mason equivalent circuit model, at resonance frequency, *f*_0_, the quality factor, *Q*, can be defined by $Q = \frac{{2\pi {f_0}{L_{\mathrm{m}}}}}{{{R_{\mathrm{s}}} + {R_{\mathrm{m}}}}}$, where *L*_m_ is a motional inductance, *R*_m_—motional resistance representing mechanical/acoustic losses, and *R*_s_*—*series resistance from electrodes, contacts, etc. LiNbO_3_, is available in the form of thin films with thicknesses >300 nm, fabricated by ion slicing or polishing processes, and offers one of the highest *K*^2^ of 25% for longitudinal BAW in comparison to other available thin films available at the industrial scale. This enabled the demonstration of TFBAR (thin film bulk acoustic resonator) and SMR (solidly mounted resonator) devices based on LiNbO_3_ films with acceptable effective *K*^2^ and operating at 6–8 GHz but limited *Q* factor [227, 228]. However, at high frequencies, acoustic material losses and power dissipation tend to increase considerably, and manufacturing tolerances to variations in film thickness and properties become very low. The polishing/trimming methods used for the fabrication of LiNbO_3_ films faces their limits in terms of thickness control.

Although the performance of SAW devices cannot compete with that of BAW filters at frequencies >4 GHz so far. New technological platforms, based on coupled acoustic waves with optic or SWs require extreme performances with respect to those of classical approaches: ultra-low-loss processing, precision control of temporal and spectral profiles of photons, phonons, electrons and spins, fast switches, etc (see other sections). To attain a high coupling of different modes, the waves have to be well confined thus suspended acoustic waveguides are frequently considered [229] even structure mechanical robustness and power handling (due to due to inefficient heat dissipation, primarily caused by limited thermal conduction through air and the absence of effective heat sinking to the substrate) are compromised. Frequently used co-planar configuration of SAW interdigital electrodes (such as delay lines), operating from 100s MHz to 3 GHz in addition present high propagation losses and cross-talk. To attain higher frequencies higher overtone modes are frequently considered although their *K*^2^ is lower than that of fundamental mode. Moreover, the footprint of present AO devices is too large. AlN films, broadly used for BAW filters at industrial scale has attracted a considerable attention for development of piezo optomechanic functions thanks to its CMOS compatibility, wide bandgap, absence of refractive effect and reasonable photoelastic properties (*p*_11_ = 0.1) [229] although it presents several times smaller photoelastic and *K*^2^ coefficients in comparison to LiNbO_3_. The key challenges for piezo-MEMS actuators are operation at high frequencies (100 MHz) and to reduce further the driving voltages.

### Advances in science and technology to meet challenges

To increase further the frequency of acoustic filters, new resonator concepts are needed (figure 25) and innovative approaches such as XBARs (X-laterally excited) [230], OBARs (O-overmoded, using second overtone of thickness mode, exited in the structure composed piezoelectric film sandwiched between two electrodes with thicknesses of ½ acoustic wavelength) [231], polarisation inverted multilayered film resonators [232] which reduces constraints of using very thin piezoelectric and electrode films (although their thickness still has to be controlled with precision) in line with innovative power management strategies for the membrane structures such as inverted structures [233] were reported. OBARs enables not only to attain higher quality factor and reduced loss at high frequencies (>30 GHz) than standard BAW due to reduced resistive loading of electrodes but also preserved 2/3 of *K*^2^ of the fundamental mode [234]. High-frequency guided SAW in highly coupled piezoelectric thin films like LiNbO_3_ on substrates or layers with higher acoustic velocity (such as SiC, WC, diamond, sapphire, BN, AlN, ZrB_2_, TiB_2_) enable to attain better wave confinement to the surface and to increase considerably SAW velocity/frequency [221, 235] which are essential for development of acoustic waveguides and devices based on the elastic wave coupling. Guided SAWs are dispersive and thus their characteristics are highly dependent on the piezoelectric layer thickness. Moreover, the guiding efficiency is attained at piezoelectric layer thickness below ¼ of SAW wavelength (<200 nm for smallest industrial IDT pitch). BAW devices could be also an efficient way to confine the acoustic energy but only high overtone BARs with poor energy confinement and low coupled AlN layers were tested [224].

Nevertheless, further advances in new piezoelectric thin films and their fabrication techniques are required. In the case of the top-down fabrication, further efforts are needed to offer LiNbO_3_ layers with better controlled and more homogeneous thicknesses over large surfaces including thin films with thicknesses <300 nm. Direct growth of high quality single crystalline LiNbO_3_ on sapphire substrates and high-performance guided SAW waves at 5.4 GHz [236] and possibility to grow highly coupled textured Y33° LiNbO_3_ orientation with physical properties close to single crystal films on bottom electrode & Bragg mirror [237] have been already demonstrated. Direct liquid injection chemical vapour deposition enables LiNbO_3_ thin film growth with homogeneous and controlled composition and thickness over large surfaces (up to 8–12 inches). Film growth opens possibilities for further optimisation of device performance in terms of Li stoichiometry and LiNbO_3_-LiTaO_3_ solid solution composition (such crystals are not available at industrial scale) to reduce photorefractive effect, to ameliorate thermal conductivity, reduce temperature coefficient of frequency (TCF) keeping reasonable K^2^, increase power handling capability by increasing the coercive field, etc. The techniques enabling *in-situ* polarisation reversal during the film growth would be a big step forward in BAW technology. Furthermore, to integrate single-crystalline quality deposited LiNbO_3_ films with SiC, diamond, or SiO_2_ containing structures for guided acoustic or optic waves, layer transfer techniques have to be developed. In the case of heterogeneous integration of LiNbO_3_ with high acoustic velocity layers/substrates, the bonding layers such SiO_2_ or Au should be replaced by direct molecular bonding, which is not straightforward due to issues related to high thermal stresses in LiNbO_3_ films. The advent of the 2D materials promises new approaches for the elimination of electrode mass effect on thin piezoelectric thin film EM coupling. Particular effort has to be given for the development of K_1−*x*_Na*_x_*Nb_1−*y*_Ta*_y_*O_3_ family thin films—materials with the highest known experimentally measured piezoelectric (*K*^2^ of SAW & BAW 53% & 88%, respectively [238]), elasto-optic (*p*_11_ in the range 0.5–0.8 [239]), electro-optic (*r*_51_ = 11 000 pm V^−1^ and *r*_33_ = 440 pm V^−1^ [240]), and nonlinear Kerr coefficients, but not available in the form of big crystals like LiNbO_3_, and their integration with photonic and acoustic devices for advanced semiconductor platforms to face future technology performance requirements.

### Concluding remarks

To summarize, the future acoustic device development requires to achieve extreme performances (high frequences, high *Q* factors, high *K*^2^, low losses, dispersion engineering, durability, etc). So far the LNOI platform seems to be the structure of choice for the acoustic devices operating at high frequencies although the control and homogeneity of thickness needs to be further ameliorated. New resonator concepts are developed to respond to the performance requirements of high-frequency acoustic filters and to offer higher efficiencies of AO coupling, spin manipulation, etc. Further maturity amelioration of LiNbO_3_ thin film growth and layer transfer techniques might help not only to reduce the fabrication costs, to overcome difficulties of thickness control but also to enable amelioration of performance by tuning stoichiometry and solid solution composition. Further advances in piezoelectric thin films such as KNbO_3_ family materials and their fabrication and integration techniques would open new avenues for highest performances at high frequencies and would offer the CMOS compatibility (Li containing materials are not compatible) with a possibility to take profit from maturity and advantages of semiconductor platforms.

## Acknowledgements

This work was supported by the French RENATECH network and its FEMTO-ST technological facility, C2N micro nanotechnologies platform, Bourgogne Franche-Comté region, the French national ANR Projects LINKS ANR20-CE08-0025, FIESTA ANR-20-CE05-0026, ANTARES ANR-24-CE51-0541, European HORIZON MSCA project HINA (Grant Agreement No. 101169557), and the graduate school EUR EIPHI Contract ANR-17-EURE-0002.

## 16. Surface acoustic waves for magnetic field sensing

### Massimiliano Marangolo^1^, Laura Thevenard^1^, Pauline Rovillain^1^, Catherine Gourdon^1^, Sami Hage-Ali^2^ and Omar Elmazria^2^

^1^ Sorbonne Université, CNRS, Institut des NanoSciences de Paris, INSP, 4 place Jussieu, F-75005 Paris, France

^2^ Université de Lorraine, CNRS, IJL, F-54000 Nancy, France

E-mail: marangolo@insp.jussieu.fr, thevenard@insp.jussieu.fr, rovillain@insp.jussieu.fr, gourdon@insp.jussieu.fr, sami.hage-ali@univ-lorraine.fr and omar.elmazria@univ-lorraine.fr

### Status

**Figure 26.**
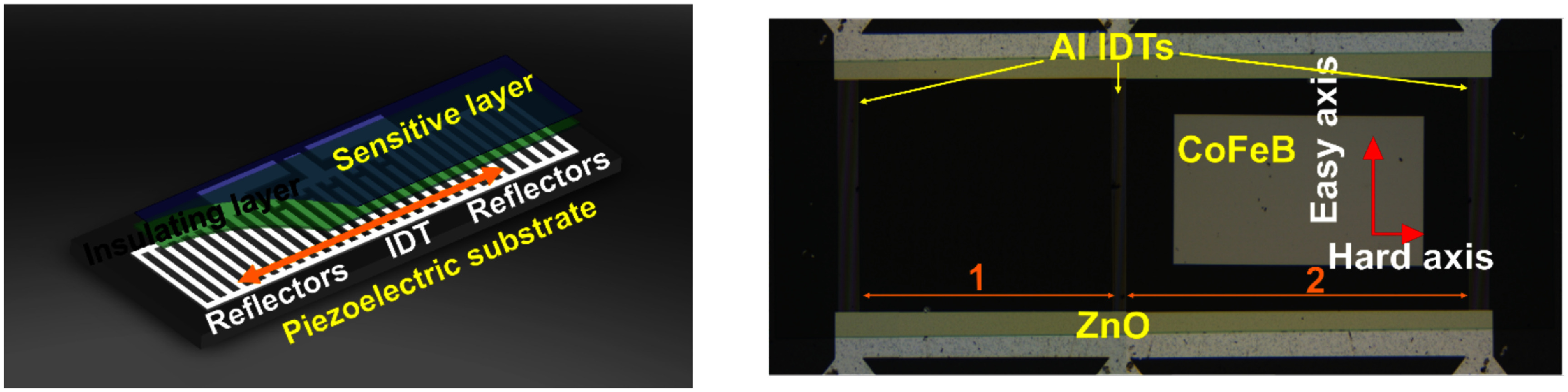
MSAW sensors. (a) Schematic view of a resonator configuration, with an insulating layer and a full film magnetoelastic layer. The SAW propagation direction is indicated in orange, (b) top view of a sample in delay line configuration with a CoFeB magnetoelastic layer between IDTs (path 2) and temperature compensation (path 1). Reproduced from [241]. CC BY 4.0.

SAW sensors are gaining prominence in various sectors including automotive, aeronautics, medical industries, and the IoT. SAW sensors are very sensitive to a large variety of physical parameters including magnetic fields when combined with a magnetoelastic layer (figure 26). Thanks to their wireless, battery-free and identification capabilities, SAW sensors offer significant advantages over conventional magnetic sensors such as magneto-resistive or inductive devices and are particularly suited for industrial monitoring, remote sensing in harsh environments, and biomedical applications.

Magnetic field-sensitive SAW devices were first introduced by Ganguly *et al* [242] as phase shifters. Magnetic field SAW sensors (MSAW) are particularly suitable for detecting weak (>1 nT), low-frequency (>1 Hz) magnetic fields and serving as current sensors due to their wide dynamic range and bandwidth. Based on magnetoelastic materials, they leverage non-linear alterations in the material’s elastic parameters caused by magnetostrictive strain, which in turn modify the SAW velocity, impacting resonance frequencies or propagation delays [243–247], and more rarely the SAW amplitude [248, 249]. The variations of each elastic constant under magnetic field, sometimes summarized using the ‘Δ*E*/Δ*G* effect’ denomination, impact differently each type of mode, for example Rayleigh or Love waves.

There are two main geometries of MSAW sensors: resonator [243] and delay line [241]. Both configurations allow for the detection of subtle magnetic field changes (figure 26). Resonators are very compact, while delay lines can yield a high absolute phase sensitivity, separate acoustic wave generation and sensing parts, and can provide identification, using reflector banks with variable delays to generate a code. The magnetoelastic material can constitute the IDTs, or be used in full film form on top of resonators [244, 245] or within delay lines [241, 246, 247, 250]. Alternatively, magnetic sensing is achievable by monitoring the amplitude changes in the SAW reflected signal, influenced by impedance changes in an externally coupled GMI sensor [248].

The applications of MSAW sensors span a broad spectrum. They can detect fields exceeding the Earth’s magnetic field (EMF) at 1 mT and higher, critical for remote magnetic field and current sensing (ex. power lines), or sensing within motors. These sensors also perform well sensing EMF disturbances ranging from 0.1 to 10 *μ*T (ex. autonomous sensors for smart parking or traffic control). Furthermore, they can achieve detection limits as low as 1 nT and below, essential for critical biomedical applications.

### Current and future challenges

Since the earlier breakthroughs [243], the field of MSAW sensors has experienced remarkable advancements in sensitivity and lower limit of detection, as evidenced in table 2. Notably, researchers from the University of Kiel have made significant contributions [241, 247]. These advances stem from intensive studies focused on engineering the properties of both the magnetoelastic layer and the piezoelectric substrate.

**Table 2.** MSAW sensors structures and performances. R: resonators, DL: delay lines, QST: quartz ST-cut.

Substrate & structures	Sensitive layer	Freq.	Max. sensitivity	Limit of detection	References
FeCoSiB/ZnO (R)	FeCoSiB	1.6 GHz	around 11 500 ppm mT^−1^	/	[243]
QST X + 90 (DL)	[TbCo_2_/FeCo]x25	1.2 GHz	2500 ppm mT^−1^	/	[246]
QST X + 90/ZnO/SiO_2_	CoFeB	1 GHz	620 ppm mT^−1^	/	[244]
(R)	FeCoSiB	450 MHz	14 000 ppm mT^−1^	/	[245]
QST X + 90/SiO_2_ (R)	FeCoSiB/NiFe/MnIr (x2)		∼45–49 000 ppm mT^−1^ @25 dBm*	∼50 pT/$\surd $ Hz at 10 Hz @25 dBm	[247]
QST X + 90/SiO_2_ (DL)		142.5 MHz	∼28 000 ppm mT^−1^ @5 dBm*	28 pT/$\surd $ Hz at 10 Hz @5 dBm	
			*assuming a delay of 1.389 ns		

Regarding the piezoelectric substrate, studies have shown that Shear and Love waves based on ST-cut quartz (QST) X + 90° (refer to table 2) or LN Y-X cut [250] typically outperform Rayleigh waves in terms of MSAW device sensitivity. This superiority is often attributed to the substantial variation of the elastic stiffness constant *C*_66_ under field [246]. Concerning the magnetoelastic overlayer, research has highlighted the critical role played by the relative angles between the acoustic wave vector, the anisotropy direction, and the magnetic field orientation [245, 246].

Moreover, to detect low magnetic fields and obtain high sensitivity sensors a balance must be found between relatively high magnetostriction and low anisotropy, with FeCoSiB appearing as the leading candidate so far. Additionally, to achieve low limits of detection, mastering the magnetic domain and domain wall configuration is essential to minimize the associated noise [251]. Moreover, the thickness and quality of the magnetoelastic layer have a significant influence on sensor performance [242, 252, 253], as magnetic sensitivity tends to amplify with these attributes.

However, numerous challenges persist in the realm of MSAW sensors [254]. Optimising operational conditions often requires applying a bias field, which can increase size and power consumption. Solutions entail engineering intrinsic bias fields in order to have high sensitivity at zero field, aligning the external field along the hard axis to have a reproductible continuous rotation of the magnetic moments and a stable sensor behaviour, always taking into consideration the occurrence of hysteretic behaviours that can hinder field determination.

Moreover, temperature sensitivity poses a significant challenge frequently overlooked in literature. This issue encompasses both the temperature coefficient of frequency (TCF) values, defined as TCF = Δ*f*/(*f*Δ*T*), and the influence of temperature fluctuations on the magnetic anisotropy within the sensitive layer. Addressing this challenge can be approached in two ways: integrating materials in multilayers to achieve nearly zero TCF and maintain magnetic stability [244], or utilising a magnetically insensitive acoustic path for temperature compensation [250]. In such cases, SAW devices become multifunctional, enabling the simultaneous sensing of multiple parameters. Additionally, due to acoustic and magnetic losses, as well as often poor impedance matching, MSAW devices frequently exhibit mediocre electrical properties for wireless operation. Exploring novel approaches such as implementing electromagnetic resonator geometries or employing insulating magnetostrictive ferromagnets could potentially solve some of these issues.

### Advances in science and technology to meet challenges

Building on recent advancements in magnonics, SAW-ferromagnetic resonance (FMR) technology holds the potential to develop sensors with significantly enhanced sensitivity and directionality due to the resonant nature of the magnetoacoustic interaction [255]. These conditions are achievable by employing low damping magnetostrictive materials with SAW-frequencies close to the FMR, known as SAW-FMR [256, 257]. The spin current emitted from the induced magnetisation precession can moreover be detected in the DC regime [128, 258], making it potentially more readily implementable, although the low signal-to-noise ratio needs to be improved.

Experimental demonstrations of SAW-FMR magnetic field sensing include Rayleigh waves traversing an epitaxial Iron film on GaAs [255] and shear horizontal-type SAWs excited in a FeCoSiB layer on LiTaO_3_ [252]. Future SAW-FMR developments target 1–10 GHz. High frequencies demand electron beam lithography for IDT fabrication, advanced signal processing, and wireless addressing. Challenges can be tackled by selecting high-phase-velocity piezoelectric substrates or adjusting the field direction to lower spin wave (SW) frequencies [255]. The integration of magnetorotational coupling notably expands the range of magnetic materials suitable for SAW-FMR devices, accommodating both magnetostrictive and non-magnetostrictive materials, such as softer, non-magnetostrictive permalloy. Additionally, the non-reciprocal nature of these interactions could enable to sense not only the field axis but also its direction as discussed in contribution 8.

Achieving a three-axis determination of the magnetic field orientation remains a challenge with MSAWs. Furthermore, the literature often overlooks the issue of very low capability to sense the out-of-plane component of the magnetic field, despite its necessity in certain space-constrained applications. Thicker magnetoelastic layers present a promising solution to address this concern.

In the context of remote magnetic sensing in harsh environments such as in the gas, aerospace, steel and automotive industries, selecting materials capable of withstanding extreme conditions is essential as detailed in contribution 17. In this context, piezoelectric substrates like Langasite can withstand temperatures of up to 1000 °C. However, their effectiveness in such harsh environments depends on their compatibility with magnetostrictive materials that preserve their magnetic properties even under such extreme temperatures.

In order to achieve extremely low detection limits, and to mitigate low-frequency noise arising from magnetic domains interacting with propagating SAWs strategies such as leveraging exchange bias with antiferromagnetic materials have been explored. For instance, Schell *et al* showcased exceptional detection capabilities, achieving remarkably low detection limits of 28 pT/Hz^1/2^ at 10 Hz and 10 pT/Hz^1/2^ at 100 Hz [247]. These results represent a significant advancement towards biomedical applications demanding the detection of extremely weak biomagnetic fields. If Magnetocardiography and very low field Magnetic Anomaly Detection applications now seem within grasp, exploring new paradigms is essential to achieve femtotesla range magnetoencephalography with MSAW technology.

### Concluding Remarks

Magnetic SAW sensors present an appealing option for various industrial uses, yet further research is necessary, especially for magnetic field sensing involving harsh environments. When compared to existing magnetic sensors on the market, MSAW sensors offer several distinct advantages. These include their capability to probe magnetic fields across a very broad spectrum of amplitudes and frequencies, the integration of wireless technology, the combination of SAW sensitivity to various physical parameters (e.g. magnetic field, temperature, strain, gases) within a single device, and the relatively low cost of fabrication. The potential applications of MSAW sensors could be further explored and require additional research, particularly in areas such as flexible and wearable devices, local 3D magnetometry for precise spatial measurements, as well as space applications where radiation resilience is critical. Finally, we believe that the recent advancements in the use of SAWs for magnonics and spintronics, particularly through the interaction between SAW and SWs in the GHz regime [**see contribution 8**], could yield significant benefits for enhanced sensing capabilities. For a deeper understanding of this topic, the reader is encouraged to consult [128]. It is essential that the magnonics and SAW communities engage in dialogue to fully realize this potential.

## Acknowledgements

M M and P R acknowledge support from the Agence Nationale de la Recherche Française (ANR) Grant No. ANR-22-CE24-0015 (SACOUMAD) and from the European Union within the HORIZON-CL4-2021-DIGITAL-EMERGING-01 Grant No. 101070536 (MandMEMS). S H A and O E acknowledge support from the ANR Grant No. ANR-20-CE42-0009 (WISSTITWIN), and the France 2030 program ‘Lorraine Initiative of Excellence’, reference ANR-15-IDEX-04-LUE. L T and C G acknowledge support from the ANR, Grant No. ANR-20-CE24-0025 (MAXSAW) and from France 2030 plan Project SWING PEPR SPIN 22-EXSP-0004.

## 17. Surface acoustic wave sensors in harsh environments

### Hagen Schmidt

Leibniz Institute for Solid State and Materials Research, Helmholtzstr. 20, 01069 Dresden, Germany

E-mail: h.schmidt@ifw-dresden.de

### Status

Almost 60 years after the first SAWs were excited by IDT structures directly on piezoelectric crystals, they have led to three very different generations of SAW applications. While frequency-selective SAW devices as the first generation are indispensable for practically every modern telecommunication system and SAW devices in the form of micro-acoustofluidic actuators (third generation) are mostly still at the research level, SAW sensors, which represent the second generation of SAW applications, are already proving their special properties in very demanding real-life scenarios.

SAW sensors are suitable for measuring physical variables such as temperature, force, torque, pressure and viscosity, but they can also be used as chemical- and biosensors. They are battery-free passive components that can be interrogated both by wire and radio. In addition to their technologically basic design, they have another decisive advantage over their semiconductor-based competitors. Due to the materials used, they can also work in harsh environments, e.g. with high radiation exposure, high electrical and magnetic field strengths, and especially at very high temperatures. These advantages, in combination with their passive mode of operation and contact-free wireless interrogation, make SAW sensors particularly attractive for a range of industrial applications.

Various robust SAW sensor solutions are currently available for monitoring industrial systems and processes under harsh environmental conditions, e.g. inside marine diesel engines directly on the crosshead bearing, on boilers of power plants, in the combustion chamber of waste power plants, inside large industrial furnaces, vacuum and plasma systems and directly on the rotors of turbine engines or embedded in additive-manufactured steel components [259–261]. In addition, the use of wireless SAW sensors for direct measurement of the food core temperature in industrial ovens as well as in consumer household ovens has proven itself for over a decade.

### Current and future challenges

The greatest challenge for SAW sensors in harsh environments is ensuring component integrity under high-temperature conditions. This applies in particular to the temperature durability of all materials and components used [259].

Although the melting temperatures of most piezoelectric crystals are well above 1000 °C, the use of standard substrates from telecommunications is limited to much lower temperatures, e.g. 573 °C for quartz due a structural phase transformation. Degradation effects have already been reported in the 300 °C–400 °C range for LiNbO_3_, yet recently investigated members of the langasite family, in particular langasite LGS itself and catangasite CTGS, do not exhibit limiting phase transitions below their melting points of over 1300 °C [262]. In addition, the successful deployment of a high-temperature resistant piezoelectric AlN thin film system on SiC substrates supporting high SAW phase velocity was recently reported [263].

In general, the material parameters of the piezoelectric substrate significantly determine the SAW properties such as phase velocity, piezoelectric coupling etc. and thus also the frequency and design principle of the sensor, i.e. either resonator (SAWR) or reflective delay line (rDL). Moreover, wireless devices, which are usually exposed to multiple signals, also have to deal with signal interference problems. SAW sensors in the preferred 2.4 GHz ISM band require high phase velocities, which can currently only be achieved with LiNbO_3_, which also enables rDLs with ID functionality. In contrast, the other substrate materials can only be used for SAWRs in the 433 MHz band due to their relatively low velocities and small coupling coefficients.

For temperature measurements, changes of the sensor frequency or of the SAW propagation time caused by the temperature dependence of the material parameters in combination with thermal expansion are currently evaluated. For an effective sensor design, it is therefore necessary to know all acoustically relevant material properties (complete elastic, dielectric and piezoelectric tensors as well as mass density) including the corresponding temperature dependencies and thermal expansion coefficients. For new crystals such as LGS and CTGS, this leads to major challenges in their precise experimental determination [264, 265].

Another challenge is the temperature durability of the electrode metallisation. In principle, refractory precious metals such as Pt and Ir can be deposited as thin films, whose melting temperatures are even far higher than those of the piezoelectric substrates. However, the main problem for such films with a thickness of only a few hundred nanometres are oxidation and diffusion-related degradation effects such as de-wetting which seriously jeopardize the integrity of the electrodes even at temperatures below 1000 °C within a few hours. The stabilisation of the electrodes therefore requires the development of dedicated material systems and technologies, e.g. for encapsulation and passivation.

### Advances in science and technology to meet challenges

**Figure 27.**
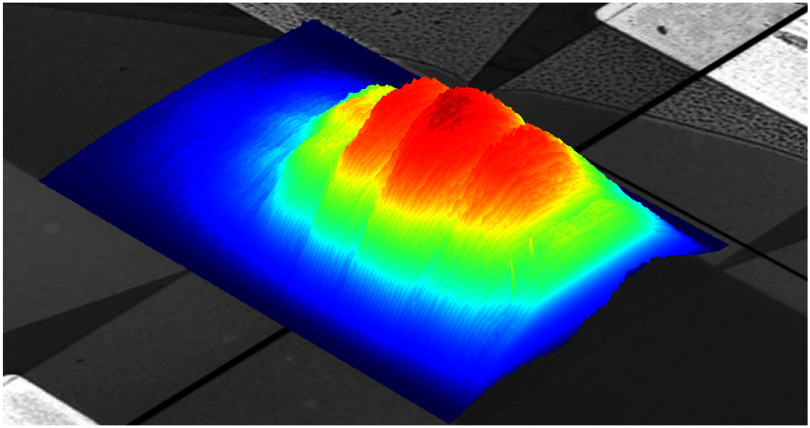
SAW wavefield measured *in-situ* at high temperature operation. Partial view of the amplitude distribution (shown in colour) of a CTGS-based 433 MHz resonator at 450 °C with local degradation of the electrode metallisation at the IDT-reflector transition.

In recent years, significant progress has been made both in terms of technology and material improvement. The size and quality of commercially-available piezoelectric wafers have been increased improving conditions for industrial deployment of SAW sensors. Furthermore, the analytical possibilities have also been improved including *in-situ* analysis of SAW wave fields under high-temperature conditions (see figure 27) allowing identification of hidden effects at device level and supporting high-performance designs. Moreover, the extensive use of advanced techniques for material analysis using SEM, FIB and TEM has increasingly contributed to the clarification of the fundamental mechanisms of temperature-induced material degradation.

Following a pragmatic approach, research on high-temperature sensors is currently pursuing several directions. One focus area are sensors for intermediate high temperatures between 300 °C and 600 °C. For this range there is a great need for cost-effective wireless sensors to monitor temperatures inside industrial and automotive systems that cannot be realized on the base of semiconductors. In contrast, SAW sensors can be manufactured to some extent using slightly modified standard manufacturing technologies and materials well-known from filter technology. For example, electrodes based on a NiAl alloy deposited on congruent-grown high-quality LiNbO_3_ already enable stable long-term operation of rDL tags at 500 °C in air for several days without significant degradation [266]. Other newly developed aluminium-based electrode systems with AlNO encapsulation realized by controlled thermal formation of either the intended AlTi phase or the refractory AlRu phase demonstrate in resonators on CTGS long-term stable operation in air up to 570 °C (AlTi [267]) or with AlRu at more than 700 °C, in vacuum are even 900 °C expected for the latter [268].

Operation at very high temperatures up to 1000 °C or even at ultra-high temperatures >1000 °C can so far only be realized with cost-intensive substrates and metallisation systems based on Pt. For example, resonators with Al_2_O_3_-encapsulated Pt electrodes on CTGS were operated between −100 °C and 1100 °C [269]. For SAWRs with Pt–Ni/Pt–Zr multilayers on LGS long-term operation at 1000 °C has been demonstrated [259]. Resonators made of Pt/Cr on LGS, completely covered with an AlN barrier layer, were frequency-stable up to 1100 °C, even 1300 °C could be reached for a few cycles [270].

Following a completely different approach, stable cycling up to 1000 °C for SAWR on LGS was realized, even without any barrier layers. Here, the electrode metallisation was deposited with high crystalline quality using low-rate magnetron sputtering of pure Pt at 630 °C and was subsequently structured by dry etching [271].

### Concluding remarks

The current trend towards increasing global interconnectivity and a bulk-collection of measurement data with small, autonomous sensors as part of the IoT and of Industry 4.0 will continue to grow in the future. Here, battery-free and wireless SAW sensors have a unique role to play. Wherever the environmental conditions are too harsh for semiconductor-based systems and conventional wired or fibre-based sensors cannot be used due to their functional principle or require too much effort for installation and operation, SAW sensors will make a reliable contribution. In order to drive this development forward, the search for even more resilient material systems and innovative sensor solutions will further enhance performance and also open up higher frequency ranges.

## Acknowledgements

Naturally, this opinion piece can only provide a very limited overview of the diverse research activities on SAW sensors for harsh environments. It is also not the outcome of the work of one individual, but is based on the results and experience gained by all colleagues at SAWLab Saxony over many years of work in cooperation with partners from research and industry. The author would like to take this opportunity to thank all participants and the funding bodies, in particular the German Research Foundation DFG and the Federal Ministry of Education and Research BMBF, for their support over more than 15 years.

## 18. Sonomechanobiology

### Leslie Y Yeo^1^, Lizebona A Ambattu^2^, Jessie S Jeon^3^ and Daesik Kwak^3^

^1^ Micro/Nanophysics Research Laboratory, RMIT University, Melbourne, VIC 3001, Australia

^2^ Micro/Nanophysics Research Laboratory, RMIT University, Melbourne, VIC 3001, Australia

^3^ KAIST, Daejeon, Republic of Korea

E-mail: leslie.yeo@rmit.edu.au, lizebona.august@rmit.edu.au, jsjeon@kaist.ac.kr and dsbout@kaist.ac.kr

### Status

While there has been extensive work to date on the use of guided acoustic waves for physically manipulating cells (see, for example, section 19 on acoustic tweezers), it was only recently discovered that such waves can also influence their internal biological machinery [272]. This possibility, in itself, is quite intriguing, not only because their wavelengths—*O*(10–100 *μ*m)—are comparable or larger than the characteristic dimension of the cell (*O*(1–10 *μ*m)), but also because cells would not normally be expected to appreciably respond to mechanical cues at frequencies considerably beyond the typical Hz-order frequencies associated with physiological motion.

A distinct advantage of high frequency (MHz-order) mechanostimulation compared to static or low frequency (mainly several Hz) mechanostimulation in conventional mechanobiology is the considerably higher cellular viabilities (typically >90%) that can be retained. This is because of the increasing difficulty in driving cavitation at high frequencies [273] given that the mechanical or cavitation index scales as the inverse of the square root of frequency or the inverse of the frequency, respectively [274], and because only very short, repeated bursts of stimuli are required (several minutes daily) to achieve similar, but more persistent and longer-term effects compared to that obtained with its static or low frequency counterpart, in which continuous treatments over days at much higher intensities are typically administered.

**Figure 28.**
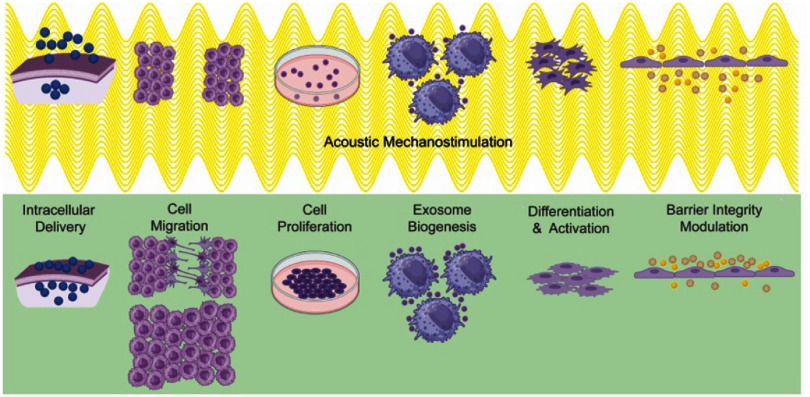
Applications stemming from the downstream cell fates following high frequency mechanostimulation of cells.

As such, we expect this discovery to spawn an entire new field of sonomechanobiology, given its ability to direct various cell fates for a number of applications (figure 28), including cytosolic intracellular delivery, cell migration and proliferation, exosome biogenesis, stem cell differentiation, endothelial barrier modulation, immune cell activation, and sperm/oocyte activation, among others [20, 272, 275–282]—in many cases without the need for additional chemical or biochemical stimuli.

Much, however, remains to be understood regarding the underlying mechanotransductive mechanisms by which the cells sense the high frequency mechanical forcing, relay these cues through the cell and nucleus [283], and how this leads to the complex transcriptomic and signalling events that result in the cell and nuclear [283] responses that eventuate. While it has been reported that Piezo channels, for example, can be activated by high frequency guided waves [276, 278, 284, 285], and that these allow the modulation of Ca^2+^ ion influx into the cell that, in turn, acts as a second messenger that triggers a host of downstream signalling cascades [272], the mechanosensory apparatus that facilitates the cellular perception of the high frequency forcing, for example, has yet to be completely elucidated. Equally, beyond the few applications that have been demonstrated, we believe the full potential of the high frequency mechanostimulation has yet to be explored, both in terms of its use as a tool for probing and understanding cellular, tissue and organ biological function, and its implications on physiological and pathological events at these different scales, together with the therapeutic applications that follow.

### Current and future challenges

Given the infancy of the field, there is thus an immediate need to conduct systematic investigations that provide a more comprehensive understanding of the phenomena in order for its potential to be fully realised. A significant challenge with such studies is the complexity of the mechanostimulation applied to the cells, which comprises a dynamic combination of compression, tension or shear. In addition, the acoustic forcing can also give rise to a number of other effects felt by the cells, in particular, acoustic RP, acoustic streaming (i.e. convective flow) in the extracellular milieu—which, in turn, can induce secondary shear effects on the cell, as well as viscous heating. It may also not be expedient to neglect the influence of the evanescent electric field associated with the EM coupling of the acoustic wave depending on the proximity of the cells to the piezoelectric substrate on which the acoustic wave propagates. Isolating each effect to ascertain which is responsible for a specific cellular mechanoresponse can therefore be especially challenging, particularly given that this typically varies across cell types. Moreover, the nonlinearity of the system is now well known, particularly from our work to date in understanding the fundamental fluid–structure coupling arising from high frequency microfluidic acoustic wave excitation, making modelling the phenomena considerably more difficult.

Future challenges are anticipated to centre around defining a clear pathway for practical realisation of the technology towards translational impact. This will largely depend on the ability to

1. Demonstrate the platform as an analytical or diagnostic tool;
2. Upscale the current microfluidic chip based technology into an efficient means for autologous *ex vivo* cell engineering or industrial scale cell bioprocessing; or,
3. Develop a method for enabling *in vivo* application.

Achieving these will require addressing current limitations associated with the miniaturised chipscale footprint of the acoustic devices, which pose constraints to direct scale-up of the technology, and the short attenuation length of the high frequency sound waves, which do not easily allow for non-invasive *in vivo* treatment that does not involve device implantation.

### Advances in science and technology to meet challenges

The ability to probe cellular responses at spatial and temporal scales commensurate with the external forcing is critical to unpacking the complex interactions associated with the high frequency mechanostimulation. While high resolution metrology and microscopy has progressed significantly to date, particularly with advances in super-resolution microscopy (such as stimulated emission depletion, photoactivated localisation microscopy and stochastic optical reconstruction microscopy) and high-speed atomic force microscopy, a means for capturing dynamical mechanobiological phenomena at high frequencies is still needed. Recent advances such as digital image correlation techniques to quantify cell deformation under ultrasonic excitation [286], for example, offer potential for carrying out such analyses, although the current limitation in temporal resolution of several kHz needs to be circumvented.

Moreover, advances in sonogenetics additionally has the potential to offer valuable insight in the understanding of sonomechanobiology. That sonogenetic responses are also observed at high MHz frequencies [287] is perhaps unsurprising given that mechanosensitive ion channels, such as transient receptor potential, TWIK (tandem of pore domains in a weak inward rectifying K^+^ channel) related K^+^ (TREK) and Piezo channels, for example, have also been found to be activated at these frequencies.

Finally, translation of the technology for *in vivo* therapeutic applications will require development of both synthetic and/or *in silico* models that recapitulate the physiological context as well as devices that can be implanted safely and efficiently. While a number of conceptual designs of chipscale SAW devices, for example, have been proposed, particularly as *in vivo* sensors for patient monitoring, we are not aware of successful demonstrations of implanted devices in clinical translation. Moreover, we note that most, if not all, sensing applications only require very low powers. By contrast, an acoustic wave actuation device would require far more power, for which a strategy for wirelessly powering the device *in vivo* would be required.

### Concluding remarks

The new and emerging field of sonomechanobiology offers exciting new possibilities to engineer cells for a many different applications, including regenerative, cancer and even cell-free (e.g. exosome) therapeutics [272]. Mechanostimulating cells at high MHz frequencies not only allows directing specific downstream cell fates similar to that observed with static and low frequency mechanostimulation, but affords far greater control over modulation of second messenger signalling whilst maintaining very high levels of cell viability, therefore facilitating effects that persist over longer terms. For the platform to be translated for cell processing or into clinical practice, nevertheless, requires further research and development to scale up the technology into a bioreactor platform that can handle throughputs commensurate with industrial scale bioprocesses, or to integrate it into an implantable device for *in vivo* therapeutics, in addition to a rigorous understanding of the fundamental mechanisms that underpin the cellular mechanoresponse to high frequency vibrational excitation.

## Acknowledgements

J J and D K acknowledge support from the National Research Foundation of Korea (RS-2025-00553725) and the Brain Korea 21 Plus project.

## 19. A new wave of precision: the rise and promise of acoustic tweezers

### Joseph Rufo, Shujie Yang and Tony Jun Huang*

Department of Mechanical Engineering and Materials Science, Duke University, Durham, NC 27708, United States of America

*E-mail: tony.huang@duke.edu

### Status

**Figure 29.**
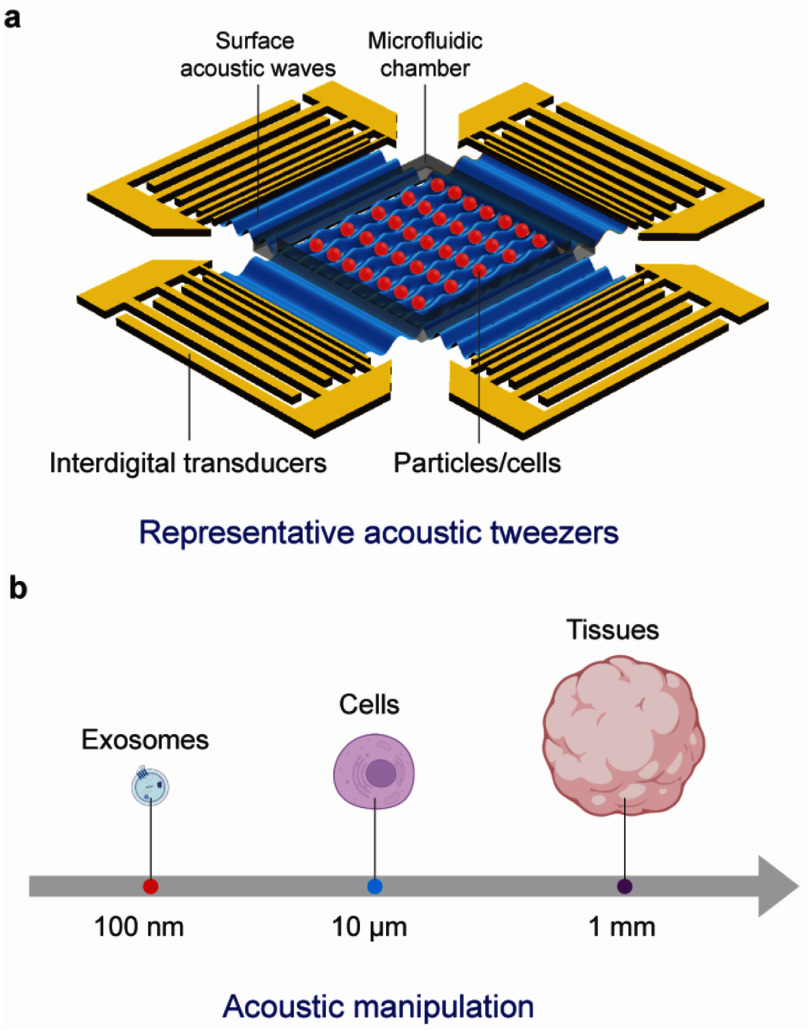
(a) Schematic representation of an acoustic tweezer device, highlighting its ability to trap single particles by applying standing surface acoustic waves. (b) Scale of objects that can be manipulated using acoustic tweezers, ranging from nanometre-sized exosomes, through larger cells at approximately 10 *µ*m, up to tissue samples >1 mm in size.

Acoustic tweezers, in their various implementations, have advanced research capabilities by enabling the precise manipulation of particles and fluids in a contact-free manner. Tracing back to the mid-19th century, scientists observed that particles aggregate at the nodes of standing acoustic waves. However, it was not until about thirty years ago that the first modern acoustic tweezers were developed, showcasing their ability to trap and manoeuvre relatively large (∼200 *µ*m) latex spheres and clusters of frog eggs [288]. Since these early, rudimentary applications, the technology behind acoustic tweezers has undergone significant refinement. Acoustic tweezers devices are now capable of performing complex, selective manipulations, including trapping, translation, and rotation, of a wide range of materials [289–293]. Their scope of operation spans from nanometre-sized bioparticles to millimetre-sized model organisms (figure 29), and they function effectively in various mediums including air, water, and even biological tissues, illustrating their versatility and increasing sophistication in modern research [294].

Recent advancements in acoustic tweezers have broadened their impact across various scientific fields. Their precision manipulation capabilities have led to innovative techniques and groundbreaking discoveries, particularly in biomedical research. The potential for non-invasive surgical applications and improved patient care is significant, extending the boundaries of minimally invasive procedures and offering new treatment options for challenging conditions. Looking to the future, the anticipated improvement in the resolution of acoustic tweezers is expected to transform cellular and molecular biology. Advancements in transducer design and wave-guide technology are set to enhance the resolution of acoustic tweezers to the nanometre scale. This breakthrough will enable highly precise applications like the stimulation of individual transmembrane proteins, paving the way for more profound insights into cellular mechanics and signalling pathways in mechanobiology studies. Furthermore, in the realm of tissue engineering, acoustic tweezers are anticipated to play a critical role in the development of advanced tissue models. These future models aim to surpass the limitations of simple single-cell-type aggregations by incorporating complex assemblies of multiple cell types, thus more accurately replicating natural tissue dynamics.

As this article delves into the innovative applications of acoustic tweezers, it becomes clear that we are on the cusp of a new wave of precision in scientific research and medical practice, driven by the continued development and application of this versatile technology. The envisioned diverse applications, from detailed biological manipulations to advanced tissue engineering, underscore their growing importance and vast potential for future scientific breakthroughs.

### Current and future challenges

Acoustic tweezers, despite their advancements, face several key challenges that limit their broad adoption in research. One of the biggest challenges is the ability to selectively manipulate a large number of particles without influencing neighbouring particles. Early demonstrations of acoustic tweezers primarily relied on standing waves to trap and move particles in grid-like patterns. However, this method was limited in its ability to manipulate individual particles. To overcome the challenge of selectively manipulating particles, several innovative strategies have been developed, including phased array transducers [290, 291], acoustic holography [292], and single beam trapping [293].

Phased array transducers are at the forefront of manipulating acoustic wavefronts. They control each pixel independently, allowing dynamic and real-time adjustments [290, 291]. However, their complexity and cost escalate with larger arrays, necessitating individual control and synchronisation of each transducer. Acoustic holography offers an alternative with a monolithic acoustic hologram. This technique, akin to an optical kinoform, shapes the output of a single transducer to create precise 3D sound fields [292]. The process achieves diffraction-limited resolution but lacks flexibility in dynamic scenarios. Single beam trapping with acoustic vortices is another notable advancement, achieving precise localisation by adding angular momentum to the field [293]. However, these vortices form a node line, not a point, along the axial direction, limiting their use to primarily 2D manipulation and reducing their effectiveness in 3D. Currently, no single technique stands out as the definitive solution for the selective manipulation of particles. In future applications, a synergistic approach, combining these techniques, may be the most effective path.

Another primary obstacle is the gap in commercialisation and seamless integration with existing research tools. To date, the most striking demonstrations of acoustic tweezers have largely been confined to the controlled environments of research laboratories, limiting their interdisciplinary significance and practical utility. While there have been recent advancements, such as the development of Lumicks’ Z-Movi for cell avidity measurements, acoustic tweezers still lag behind more established technologies like optical tweezers, which boast a variety of commercialized instruments. Recent demonstrations of acoustic tweezers inside of Petri dishes represent a promising first step [295]; however, the journey towards widespread, practical application is still underway. To bridge this gap, further innovation and collaboration between researchers and industry are essential. Efforts must focus on simplifying the technology for user-friendly operation, increasing compatibility with existing tools, and scaling production for commercial availability.

### Advances in science and technology to meet challenges

One significant area of progress in acoustic tweezers is their enhanced capacity for selective manipulation, evolving from manipulating particles in fixed grid patterns to dynamically controlling individual particles. Historically, acoustic streaming, a fluid flow induced by the acoustic field, was often considered a drawback in acoustic tweezers, as it could lead to unintended particle movements, disrupting precise manipulation. However, recent advancements have turned this perceived limitation into an asset. By exploiting acoustic streaming, either independently or in combination with acoustic radiation force, researchers have enabled selective 3D manipulation of particles and droplets [296], facilitating high-throughput laboratory automation while preventing cross-contamination.

Recent years have seen remarkable progress in the development of *in vivo* acoustic tweezers, with applications ranging from non-invasive surgery to targeted drug delivery. One notable advancement involves using ultrasound beams, shaped and steered by a phased array, to manipulate solid objects within tissue [290]. This technology has demonstrated the ability to control objects, such as guiding ingestible cameras or aiding in kidney stone expulsion, showing a deviation of less than 10% from intended paths in live animal models without causing tissue damage. Additionally, acoustic vortex tweezers have been developed to trap and concentrate microbubbles or engineered bacteria in the bloodstream. Advancements in genetic engineering have led to bacteria capable of producing gas vesicles, increasing their acoustic sensitivity. These engineered bacteria can be clustered and steered *in vivo* using acoustic tweezers [291], showing potential in targeted therapies, such as improving drug aggregation in tumours. Collectively, these developments in acoustic tweezers technology mark a significant leap forward in medical applications, paving the way for more precise, non-invasive treatments and diagnostics.

**Figure 30.**
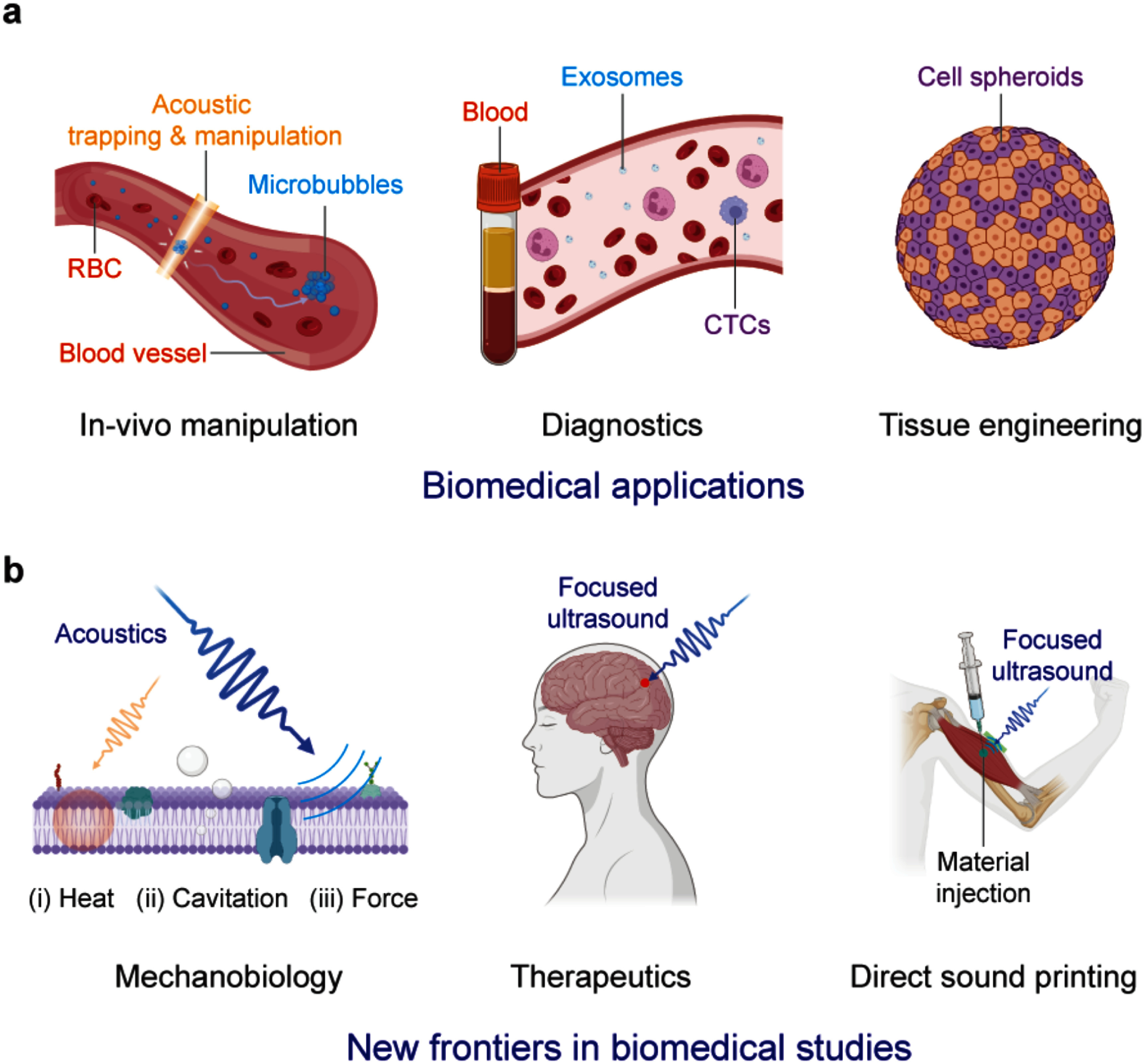
(a) Applications of acoustic tweezers across different biomedical domains: targeted drug delivery via the trapping of microbubbles; diagnostics through the isolation of circulating tumour cells (CTCs) and exosomes from blood samples; and the arrangement of cell spheroids in tissue engineering to create organized structures. (b) Illustration of emerging applications for acoustic tweezers in the advancement of multimodal biomedical tools: mechanobiology, where acoustic energy is used to generate heat, cavitation, or radiation forces to affect cellular behaviour; therapeutic applications, where focused ultrasound facilitates targeted treatment and stimulation; and direct sound printing, where focused ultrasound enables the precise placement and assembly of materials for tissue engineering and regenerative medicine. These innovative approaches illustrate the potential of acoustic tweezers to not only manipulate cells and particles but also to influence biological processes and outcomes, paving the way for the development of multimodal acoustic tweezer technologies.

The evolution of acoustic tweezers is charting a new course in biomedical research, blending refined manipulation with insights into cellular response to acoustic stimulation (figure 30). High-frequency ultrasound, a focal point in mechanobiology studies, is emerging as a powerful tool for applications ranging from selective cell stimulation [295] to *in vivo* 3D printing [297]. This union of precise manipulation with functional modulation heralds the era of multimodal acoustic tweezers, capable of not just precise positioning but also altering cellular activity. Such advancements are setting the stage for acoustic tweezers to revolutionize medical treatments and diagnostics, offering a future where targeted, non-invasive interventions at the cellular level become a mainstay in personalized medicine.

### Concluding remarks

Acoustic tweezers have made significant strides, evolving from basic particle manipulation to advanced tools capable of precise control over a broad range of materials. This is particularly evident in the realm of biomedical research, where they have expanded into critical areas like non-invasive surgery and targeted drug delivery. Their capability to manipulate objects within the body and concentrate microbubbles for drug delivery highlights their importance in modern medicine. Looking ahead, acoustic tweezers are on track to become multifunctional tools, capable of not just precise positioning but also altering cellular functionality. This evolution heralds a new era in biomedical research, focusing on targeted, non-invasive treatments integral to personalized medicine. Moreover, there are still many exciting areas to explore. As the resolution of acoustic tweezers improves and the operational frequency extends into the GHz range, we anticipate the emergence of entirely new fields of study. One such promising area is the influence of GHz acoustics on biological systems, a realm barely explored but ripe with potential. These advancements suggest that the journey of acoustic tweezers is far from complete, with their full capabilities yet to be fully realized and harnessed.

## Acknowledgements

The authors acknowledge support from the National Institutes of Health National Institutes of Health (R01HD103727, UH3TR002978, U18TR003778, R01GM132603, R01GM141055, R01GM143439, R01GM135486, R21HD102790, R01GM145960, R01GM144417, R01AG084098, R44OD024963, R44HL140800, and R44AG063643) and National Science Foundation (CMMI-2104295). The authors also extend acknowledgement to BioRender.com for creation of images.

## Data Availability

No new data were created or analysed in this study.
